# Deep residual learning for molecular force fields

**DOI:** 10.1038/s41467-026-74983-0

**Published:** 2026-06-30

**Authors:** Xinyu Jiang, Mingan Chen, Chuanlong Zeng, Duanhua Cao, Jie Yu, Runze Zhang, Zunyun Fu, Zhehuan Fan, Jiacheng Xiong, Xutong Li, Xiaomin Luo, Dan Teng, Mingyue Zheng

**Affiliations:** 1 Drug Discovery and Design Center, State Key Laboratory of Drug Research, Shanghai Institute of Materia Medica, Chinese Academy of Sciences, Shanghai, China; 2 University of Chinese Academy of Sciences, Beijing, China; 3 School of Physical Science and Technology, ShanghaiTech University, Shanghai, China; 4 Lingang Laboratory, Shanghai, China; 5 Innovation Institute for Artificial Intelligence in Medicine of Zhejiang University, College of Pharmaceutical Sciences, Zhejiang University, Hangzhou, Zhejiang China; 6 School of Information Science and Technology, ShanghaiTech University, Shanghai, China; 7 Science for Life Laboratory, Department of Cell and Molecular Biology, Uppsala University, BMC, Uppsala, Sweden; 8 iHuman Institute, ShanghaiTech University, Shanghai, China

**Keywords:** Computational chemistry, Molecular dynamics, Computational models

## Abstract

Accurate descriptions of interactions between atoms are essential for molecular simulations used to study biology and support drug discovery. Existing force fields often face a trade-off between physical reliability, computational efficiency, and accuracy across unfamiliar molecules. Here we show that Residual Learning Force Field, a hybrid machine learning force field, can reduce this trade-off by combining simple physics-based descriptions of bonded interactions with learned corrections for remaining energetic effects. The two components are trained together through a three-step strategy so that each contributes complementary information. In tests covering drug-like molecules, molecular dimers, torsional energy profiles, energy-minimum structures, and biomolecular simulations, Residual Learning Force Field gives accurate and stable predictions across diverse systems. These results suggest that combining physical constraints with data-driven corrections can provide a practical route toward more reliable and efficient molecular simulation for biological research and drug discovery.

## Abstract (web summary)

Molecular force fields underpin biomolecular simulation but often trade accuracy for generality. This study presents ResFF, a residual-learning model that combines physical rules with neural corrections to improve accuracy while retaining generality.

## Introduction

Fundamental biological phenomena, ranging from molecular recognition to allosteric modulation of protein functions, are inherently governed by complex interatomic interactions that dictate biomolecular dynamics. For decades, large-scale molecular simulations aiming to unravel these processes have relied on Molecular Mechanics Force Fields (MMFFs) as their computational foundation^1–3^. Prototypical examples, including the Open Force Field (OpenFF)^4,5^, General Amber Force Field (GAFF)^6^, and Merck Molecular Force Field (MMFF94s)^7^, among others^8–10^, employ analytical expressions for covalent and noncovalent interactions to enable efficient characterization of conformational energies^11–13^. Concurrently, recent breakthroughs in deep learning^14,15^, coupled with the exponential growth of open-source quantum mechanics (QM) datasets^16–21^, have spurred the development of neural network force fields (NNFFs) as a transformative alternative for molecular modeling. In contrast to MMFFs’ reliance on predefined analytical forms, NNFFs directly learn intricate interatomic interactions from QM data using only atomic elements and coordinates^22–28^. Advanced implementations such as NequIP^29^, TorchMD-Net^30,31^, ViSNet^32^, and MACE^33,34^ leverage geometric equivariant neural networks to achieve notable accuracy across diverse benchmark datasets.

Despite their notable advancements, both classes of force fields face critical limitations in accuracy and generalizability^35^. MMFFs have enabled large-scale simulations by providing computationally tractable approximations of quantum mechanical interactions^36,37^. However, they struggle to accurately characterize subtle noncovalent interactions, such as hydrogen bonding and dispersion forces, which are fundamental to molecular recognition and function despite their weaker magnitude relative to covalent bonds^38–40^. Efforts to enhance MMFFs with complex functional forms (e.g., polarizability corrections)^41^ have improved performance, yet they still cannot match the accuracy of quantum chemical calculations^42^.

In contrast, NNFFs can achieve near QM accuracy within the training data distribution. However, their generalization to unseen molecular systems remains unproven, with multiple studies reporting suboptimal performance in realistic molecular dynamics applications^2,43–45^. Moreover, these NNFFs typically require extensive computational resources due to their complex architectures and heavy parameterization, thus limiting their application for large-scale simulations compared to the efficiency of MMFFs^12,46,47^. This dichotomy underscores the need for a hybrid approach that merges the physical interpretability of MMFFs with the learning capacity of neural networks.

To address the trade-off between accuracy and generalizability, several hybrid approaches integrating MMFFs and NNFFs have been developed. Wang et al.^48^ originally introduced the Espaloma framework and Takaba et al. subsequently developed an improved version^49^. This framework uses graph neural networks (GNNs) to learn parameters for traditional covalent terms directly from QM data, significantly enhancing the accuracy of covalent energy calculations. However, Espaloma still relies on OpenFF’s pre-existing parameters for noncovalent interactions. AI^2^BMD^3^, a biomolecular dynamics framework for accurately simulating full-atom large biomolecules, also employs conventional MMFF parameters to describe noncovalent interactions between protein fragments, while using ViSNet^32^ to model atomic interactions within individual protein fragments. Chen et al.^50^ subsequently developed GB-FFs GAFF, which reparameterizes GAFF while maintaining its original functional forms. Similarly, Zheng et al.^51^ proposed the ByteFF, which incorporates the QM Hessian matrix as an additional learning objective and employs advanced training strategies and datasets to optimize GAFF parameters. Although GB-FFs GAFF and ByteFF offer more end-to-end solutions than Espaloma, their rigid molecular mechanics functional forms inherently restrict the model’s ability to capture the full potential energy landscape, as neural networks struggle to assign optimal parameter distributions across different energy terms during training. Other hybrid methods, such as FENNIX^52^, ANA2B^53^ and SO3LR^54^, separate molecular interactions into an intramolecular component modeled by a neural network and an intermolecular component described using classical descriptions. However, classical descriptions often fall short in accurately approximating the intricate nature of intermolecular interactions at QM accuracy. To overcome this limitation, Illarionov et al.^55^ employed a delta-learning approach called ARROW-NN^56,57^, where neural networks are applied to augment the intermolecular interactions. ARROW-NN directly utilizes neural networks to model the residual errors between QM energies and those predicted by the ARROW force field^56,57^. While this method exhibits strong performance on specific systems, it suffers from two key limitations that restrict its broader applicability. First, the ARROW force field serves as a fixed baseline that does not participate in training. This fixed baseline prevents ARROW-NN from achieving synergistic refinement between analytical and neural network components that could enhance overall performance. Second, the ARROW force field already includes both covalent and noncovalent interaction terms, creating an overlap with the noncovalent corrections learned by the neural network. This overlap complicates the effective decoupling of covalent and noncovalent contributions, thereby limiting the method’s generalizability across diverse molecular systems.

The integration of deep learning with force fields has inspired fruitful advancements, yet it also leads to our reflection on whether a more efficient and balanced approach might exist. While covalent interactions often constitute a major component of intramolecular energetics^58,59^, the accurate parameterization of noncovalent terms remains challenging due to their inherently weak, anisotropic nature and the limited availability of high-resolution experimental or high-level QM data^5,39–41,60,61^. Building on these insights, we hypothesize that a hybrid strategy that retains learned analytical molecular mechanics (MM) covalent terms and employs a lightweight equivariant neural network for residual energy correction would offer an efficient pathway to construct a generalizable force field. To facilitate synergistic coupling between the two parts, we introduce a three-stage training paradigm: (1) initial optimization of MM covalent terms to capture dominant energetic contributions; (2) fixed MM terms with a neural network learning residual energy discrepancy; (3) joint fine-tuning of all parameters for end-to-end optimization. This approach mirrors the residual learning framework of ResNet^62^, where the MM module serves as an identity mapping baseline, and the neural network learns residual corrections to augment this baseline. It is worth noting that the two components of our model are trained in a complementary manner to enhance the representation power. This approach differs from the delta-learning method we mentioned before, where the model’s performance is inherently limited by the fixed baseline component. By explicitly separating energy prediction into physics-informed MM terms and data-driven residual interactions, we propose the Residual Learning Force Field (ResFF), which integrates physical constraints with neural network expressivity, enabling both generalization and accuracy. The residual learning mechanism ensures that the equivariant neural network component directly models complex noncovalent interactions from interatomic distances without being constrained by traditional functional forms, while preserved MM terms enhance physical interpretability and extrapolation capability.

In this work, we conduct comprehensive experiments to validate ResFF’s performance, highlighting the critical role of its residual learning architecture. By integrating explicit physics-informed MM terms with lightweight neural network residual corrections, ResFF demonstrates exceptional generalization on the Gen2-Optimization dataset^63^ (~ 200,000 drug-like molecules) and DES370K dataset^64^ (~ 300,000 simple dimers), outperforming classical and neural network force fields in energy prediction for unseen structures. This performance stems from the residual learning paradigm, where the MM module provides a physical baseline, and the neural network captures residual but complex interatomic interactions. Further trained on the SPICE^21^ dataset, ResFF is evaluated across diverse molecular simulation tasks: torsional profiles using TorsionNet-500^65^ and Torsion Scan^66^, intermolecular interactions via S66×8^67^, energy minima reproduction on the OpenFF Industry Benchmark^68^, and molecular dynamics of biological systems. In all benchmarks, ResFF’s residual learning framework enables consistent accuracy and generalizability by decomposing energy prediction into MM-dominated baselines and data-driven residual corrections, it balances physical interpretability with learning capacity. These results establish ResFF as a robust tool for molecular simulation, leveraging residual learning to enhance both accuracy and practical applicability in drug discovery.

## Results

### The ResFF architecture

**Fig. 1:**
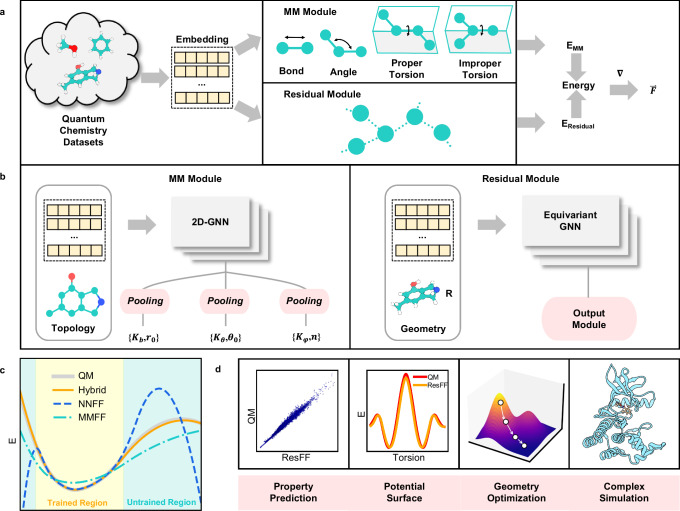
Architecture of ResFF: integrating physics-informed molecular mechanics (MM) terms and data-driven residual corrections. **a** Schematic pipeline of ResFF. The model consists of two core modules: an MM module that predicts energies from analytical covalent functions, and a residual module that captures additional energy contributions. The total molecular energy is the sum of outputs from both modules. **b** Detailed architecture of the MM module and residual module. The MM module estimates force field parameters for bonds {${K}_{r}$,${r}_{0}$}, angles {${K}_{\theta }$,${\theta }_{0}$}, and proper/improper torsions {${K}_{\varphi }$,n}, and calculates covalent energy using classical analytical functions based on atomic coordinates (R). The residual module employs an equivariant neural network to map atomic embeddings and molecular geometry to residual energies, primarily correcting for noncovalent interactions. **c** Schematic comparison of potential energy profiles predicted by different force fields. NNFFs achieve high accuracy within the training configurational space but may exhibit performance degradation when extrapolating to unseen regions. Classical MMFFs generalize better to untrained regions but lack accuracy. A hybrid approach, like ResFF, aims to combine these strengths: closely matching QM references across all configurational spaces while avoiding extrapolation failures. **d** Key downstream applications enabled by ResFF, including molecular dynamics simulations, conformational energy prediction, and modeling intermolecular interactions.

As illustrated in Fig. 1a, b, ResFF predicts energy in an end-to-end manner, integrating both MM and residual modules.

Since the MM covalent terms are well established to capture the dominant energetic contributions, as demonstrated in refs. ^48,49^, and can be effectively fitted to large amounts of QM training data, we adopted them for the MM module. In this module, 2D graph neural networks (2D-GNN) are used to generate parameters of analytical functions for covalent terms (bond, angle, torsion), which depend solely on atomic types and molecular topology. These parameters are fixed during simulations and serve as a physical baseline analogous to the identity mapping in ResNet. The MM module in ResFF thus preserves essential molecular information and provides a stable foundation for energy prediction. We also performed an energy decomposition on the inference results of ResFF, and the findings were consistent with our expectation that covalent energy contributes the most of intramolecular energetics, as detailed in Methods.

Complementing this, the residual module deploys Equivariant Transformer (ET)^30,31^, an equivariant GNN (3D-GNN) to incorporate geometric features, learning subtle interatomic interactions not captured by MM terms. This module refines predictions by modeling residual energy from the MM baseline, akin to residual mappings in ResNet that correct for discrepancies between initial and target outputs. By summing MM and residual energies, ResFF derives total molecular energy, with atomic forces calculated as the negative gradient of this total energy. Although electrostatics and dispersion possess well-established analytical forms, the residual module in ResFF is not intended to replace those physically motivated treatments. The residual term should be interpreted as a short-range, environment-dependent correction that compensates for systematic deficiencies of the analytical MM backbone in the near-equilibrium interaction regime. This design philosophy contrasts with approaches that explicitly embed long-range electrostatics into local neural network potentials; rather than reformulating analytical long-range physics, ResFF prioritizes to enhance near-equilibrium energetic fidelity while preserving a clear separation of roles within a hybrid framework.

This residual learning architecture decomposes energy prediction into physics-informed baselines and data-driven corrections, enabling ResFF to leverage neural networks for subtle interatomic interaction modeling while maintaining interpretable MM term representations. As illustrated in Fig. 1c, this hybrid approach, which integrates the strengths of both NNFFs and MMFFs, is expected to closely track the QM accuracy and perform well on unseen data. Consequently, after evaluating on several downstream tasks (Fig. 1d), this design is demonstrated to enhance both accuracy and generalizability across conformational energy prediction, modeling intermolecular interactions, geometry optimization and stable molecular dynamics simulations. More details about our model can be found in Methods.

### ResFF demonstrates robust extrapolation on Gen2-Opt and DES370K benchmarks

To evaluate ResFF’s capacity to predict total energies for unseen monomers and dimers, which is critical for real-world applications across extensive chemical space, we compared ResFF against established MMFFs, including OpenFF and GAFF, and NNFFs, such as NequIP, MACE-OFF(S), and Espaloma-0.3. Each dataset (Gen2-Opt monomers and DES370K dimers) was partitioned into training, validation, and test sets at an 8:1:1 ratio, ensuring test molecules remained unseen during training of NequIP, MACE-OFF(S), and ResFF. This data split differs from the common practice on the QM9 and MD17 datasets, where molecular conformations are randomly divided into training, validation, and test sets. Instead, it ensures that the model’s test performance cannot be attributed to memorizing molecular graphs already present in the training data. Espaloma-0.3 was utilized in its published form ref. ^49^ without retraining for these extrapolation experiments. It should be noted that MMFFs and Espaloma-0.3 were parameterized using QM data at different levels of theory compared to ResFF.

**Fig. 2:**
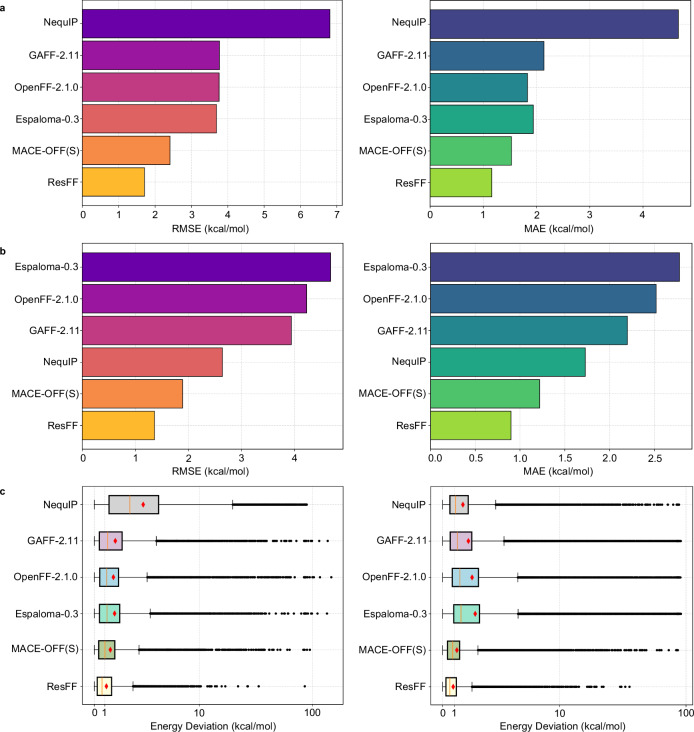
Performance of different force fields on the Gen2-Opt test set and DES370K test set. **a**, **b** RMSE and MAE for the Gen2-Opt test set (**a**) and DES370K test set (**b**). **c** Energy deviation distributions for the Gen2-Opt test set (left) and DES370K test set (right). The mean energy deviation is indicated by a red diamond, and the median is represented in an orange solid line. Source data are provided as a Source Data file.

The performance of ResFF and other force fields on Gen2-Opt and DES370K test sets reveals the distinct advantages of our hybrid architecture. As summarized in Table 1 and Fig. 2, ResFF consistently delivers strong results, with a notable mean absolute error (MAE) of 1.16 kcal/mol and Pearson’s correlation coefficient r of 0.893 on Gen2-Opt test set, and the highest r of 0.987 on DES370K test set, which evaluates noncovalent interactions within the hybrid force field framework. Classical MMFFs like OpenFF and GAFF exhibit moderate results, with *r* ≈ 0.6 on Gen2-Opt and *r* ≈ 0.8 on DES370K. Despite not being trained on these datasets, their physics-based functional forms enable generalizability across tasks with sacrificed accuracy. Espaloma-0.3 shows results comparable to MMFFs, likely because its neural network only learns covalent interactions while relying on OpenFF parameters for noncovalent terms. Their comparatively lower accuracy on DES370K gas-phase interaction energies should be interpreted in light of their original parameterization philosophy. Because the parameterization targets macroscopic thermodynamic behavior, complex intermolecular effects present in the condensed phase are implicitly absorbed into the effective nonbonded terms of classical fixed-charge force fields. However, it also implies that isolated gas-phase dimer interaction energies are not necessarily reproduced with the same fidelity.

**Table 1:** Performance of different methods on the Gen2-Opt test set and DES370K test set

Dataset	Methods	RMSE (kcal/mol)	MAE (kcal/mol)	Pearson’s r	R^2^
Gen2-Opt	OpenFF-2.1.0	3.76	1.83	0.630	− 0.088
	GAFF-2.11	3.77	2.14	0.609	/
	NequIP	6.81	4.66	0.473	− 2.574
	MACE-OFF(S)	2.41	1.53	0.778	0.551
	Espaloma-0.3	3.69	1.94	0.644	− 0.051
	ResFF	**1.71**	**1.16**	**0.893**	**0.774**
DES370K	OpenFF-2.1.0	4.23	2.52	0.826	/
	GAFF-2.11	3.94	2.20	0.837	/
	NequIP	2.64	1.73	0.957	0.913
	MACE-OFF(S)	1.89	1.22	0.977	0.955
	Espaloma-0.3	4.68	2.78	0.768	/
	ResFF	**1.36**	**0.90**	**0.987**	**0.973**

Metrics include root mean squared error (RMSE), mean absolute error (MAE), Pearson correlation coefficient r, and coefficient of determination R^2^. All metrics were computed using per-molecule mean-centered relative energies, where both predicted and reference energies were shifted by their corresponding molecular means before evaluation. Best results are in bold, and second-best results are underlined. NequIP and MACE-OFF(S) results are derived from models trained using the settings provided in Methods.

Notably, although NequIP and MACE-OFF(S) were specifically trained on Gen2-Opt, their performance remains suboptimal (*r* = 0.473 and 0.778, respectively). NequIP demonstrates a large MAE of 4.66 kcal/mol, indicating significant overfitting to the training set. This contrast with the reported in-distribution performance of NNFFs on datasets like QM9 and MD17^22^, underscoring the challenges in extrapolating to previously unseen chemical space. While NequIP and MACE-OFF(S) improved on DES370K—likely due to overlap between training set monomers and test set dimers—their results still lag behind those of ResFF.

These findings validate the superiority of the ResFF’s design. By preserving analytical MM terms, it inherits MMFFs’ physical robustness and generalizability, while the residual component leverages NNFFs’ learning capacity to model complex noncovalent interactions that MMFFs struggle to describe. This synergy enables ResFF to balance accuracy and generalizability effectively, leading to the best performance on Gen2-Opt and DES370K datasets.

Motivated by this robust extrapolation, we further trained it on the large-scale QM dataset SPICE to explore its utility in more realistic applications. The corresponding test set results are summarized in Supplementary Table 1. In this section, all baseline NNFF models are trained using energy labels as small-scale tests to provide a consistent and conservative evaluation of generalization performance. These results served controlled reference baselines trained under the same data availability and supervision setting as ResFF. For applications targeting production-quality force fields, training with both energy and force labels is generally a more appropriate and effective strategy, which is consistent with the following observations from models trained on the SPICE dataset. Subsequent references to “ResFF” denote the SPICE-trained model.

### ResFF accurately reproduces torsional profiles

Accurate prediction of torsional potential energy profiles is fundamental to molecular simulations and drug discovery, as small molecules often undergo substantial conformational changes during protein binding^69–71^. MMFFs face challenges in capturing torsional strain precisely, requiring extensive parameter fitting for torsion terms and often underrepresenting noncovalent interactions during torsional motions^69^. We compared ResFF’s performance in reproducing torsional profiles against prevalent MMFFs and NNFFs on the TorsionNet-500 and Torsion Scan benchmarks. To mitigate systematic errors from QM level differences, we recomputed both benchmarks using ωB97M-D3(BJ)/def2-TZVPPD, updating the original B3LYP/6-31G(d) calculations.

**Fig. 3:**
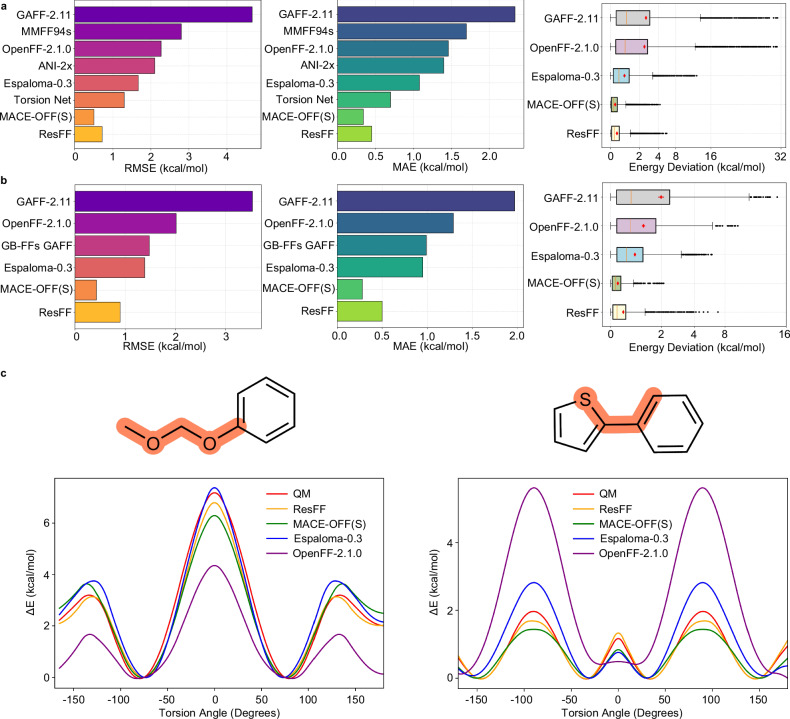
Torsional profile prediction performance across benchmark datasets. **a** Performance metrics (RMSE, MAE, and energy deviation) on the TorsionNet-500 dataset. **b** Corresponding metrics on the Torsion Scan dataset. The mean energy deviation is marked by a red diamond, and the median is represented in an orange solid line. **c** Examples of predicted torsional potential energy surfaces from the TorsionNet-500 and Torsion Scan datasets, compared with QM references. The vertical axis shows relative energies normalized to each method’s minimum energy point; the torsion angle of interest in each molecule is highlighted in red. Source data are provided as a Source Data file.

As shown in Table 2 and Fig. 3a, b, ResFF demonstrates remarkable accuracy with an MAE of 0.45 kcal/mol on the TorsionNet-500 benchmark and 0.50 kcal/mol on the Torsion Scan benchmark, outperforming most of the contemporary models and approaching the accuracy of MACE-OFF(S). As the top-performing NNFF, MACE-OFF(S) achieves the lowest MAE, surpassing ANI-2x and Torsion Net. This result validates the efficacy of the equivariant representation for learning nuanced interactions, underscoring the rationale behind our choice of an equivariant neural network for ResFF’s residual module. Importantly, the primary goal of ResFF is not to outperform the largest or most expressive neural network force fields in absolute accuracy. Rather, ResFF is designed as a hybrid force field that targets a balanced trade-off between generalization, accuracy, and computational efficiency for drug-like molecular systems. The small MAE difference between ResFF and MACE-OFF(S) aligns with our design principle of prioritizing a robust balance between predictive accuracy and extrapolation capability. ResFF’s analytical MM terms, while ensuring generalizability to unseen systems, inherently limit overfitting to training data—this explains its slightly higher MAE on these benchmarks.

**Table 2:** Performances of different methods on the TorsionNet-500 dataset and the Torsion Scan dataset

Dataset	Methods	RMSE (kcal/mol)	MAE (kcal/mol)	Pearson’s r	R^2^
TorsionNet-500	OpenFF-2.1.0	4.66	2.34	0.582	/
	GAFF-2.11	4.74	2.45	0.568	/
	MMFF94s	2.8	1.7	0.71	/
	ANI-2x	2.1	1.4	0.75	/
	Torsion Net	1.3	0.7	0.91	/
	Espaloma-0.3	1.67	1.08	0.879	0.697
	SO3LR	1.51	1.03	0.86	/
	MACE-OFF(S)	**0.51**	**0.34**	**0.982**	**0.965**
	ResFF	0.72	0.45	0.965	0.930
Torsion Scan	OpenFF-2.1.0	2.02	1.29	0.862	/
	GAFF-2.11	3.54	1.97	/	/
	GB-FFs GAFF	1.48	0.99	/	/
	Espaloma-0.3	1.39	0.95	0.915	0.806
	MACE-OFF(S)	**0.43**	**0.28**	**0.988**	**0.976**
	ResFF	0.90	0.50	0.953	0.896

Metrics include RMSE, MAE, Pearson correlation coefficient r, and coefficient of determination R^2^. Best results are in bold, and second-best results are underlined. MMFF94s, ANI-2x and Torsion Net results on TorsionNet-500 dataset are adopted from Rai et al.^65^, while GAFF-2.11 and GB-FFs GAFF results on Torsion Scan dataset are from Sellers et al.^50^. SO3LR result is collected from Kabylda et al.^54^. Espaloma-0.3 and MACE results are generated using their published model weights, detailed in the Methods.

In ResFF, the physically motivated MM module provides a stable baseline with fixed atom-type parameters assigned once at the beginning of the simulation. Classical MM force fields, however, are known to exhibit an intrinsic accuracy ceiling due to harmonic bonded approximations and simplified nonbonded forms. To mitigate these systematic deficiencies without sacrificing physical interpretability or simulation stability, we introduce a compact residual neural network (~320k parameters) that corrects the MM energy locally while preserving its inductive bias. In this context, MACE-OFF(S) (~690k parameters) serves as the most appropriate scale-matched comparison for the residual module of ResFF, as both operate within a similar parameter regime. As an exploratory comparison, we also evaluated MACELES-OFF, which incorporates explicit physics-based long-range electrostatics, as well as larger MACE-OFF variants, with the results summarized in Supplementary Table 2. As expected, increasing model capacity or introducing non-local interactions generally improves accuracy. Although ResFF uses a lightweight architecture, the residual module effectively corrects the molecular mechanics baseline and enables chemically accurate energy predictions.

As illustrated in Fig. 3c, we present two representative examples of torsional profiles across different methods, with additional cases provided in Supplementary Figs. 1 and 2. Notably, ResFF closely reproduces most of the QM torsional profile—even for geometries far from equilibrium—without the additional torsional training data typically required by classical MMFFs. This capability remains challenging for traditional MMFFs, whose simplistic functional forms often fail to adequately capture intricate QM-level interatomic interactions despite labor-intensive parameter fitting.

As low-energy regions dominate conformational sampling in molecular dynamics simulations, accurate representation of these regions is essential. We therefore evaluated whether each method correctly identifies the QM global minimum torsion angle in the TorsionNet-500 dataset. MACE-OFF(S) reproduces the QM minimum in 85.6% of cases, while ResFF achieves a comparable rate of 83.0%, indicating similar performance in locating thermodynamically relevant conformations. To further assess near-equilibrium accuracy, we computed the energy errors near the minima by selecting the four conformations closest to each QM minimum. In these low-energy regions, MACE-OFF(S) yields an MAE of 0.21 kcal/mol, and ResFF 0.29 kcal/mol. Both errors are well within thermal energy at room temperature (~ 0.6 kcal/mol), suggesting that ResFF provides an accurate and physically reasonable description of near-equilibrium regions relevant for MD sampling. While accurate modeling of equilibrium regions is essential, avoiding systematic underestimation or overestimation of torsional barriers is equally important for maintaining realistic transition behavior. Our analysis further reveals that while MACE-OFF(S) demonstrates favorable overall MAE performance, it tends to underestimate torsional energy barriers, as evident in Fig. 3c. To quantify this tendency, we calculated the maximum-minimum energy difference $(\Delta {E}_{{{\rm{barrier}}}})$ for ResFF, MACE-OFF(S) and QM reference, defining the deviation as $\Delta \Delta {E}_{{{\rm{barrier}}}}=\Delta {E}_{{{\rm{barrier}}}}\left({{\rm{Method}}}\right)-\Delta {E}_{{{\rm{barrier}}}}\left({{\rm{QM}}}\right)$. An ideal force field should exhibit a mean $\Delta \Delta {E}_{{{\rm{barrier}}}}$ close to zero, indicating no systematic bias. Our results reveal that MACE-OFF(S) exhibits a mean $\Delta \Delta {E}_{{{\rm{barrier}}}}$ of − 0.18 kcal/mol, whereas ResFF displays a mean $\Delta \Delta {E}_{{{\rm{barrier}}}}$ of 0.02 kcal/mol. Taken together, these results indicate that ResFF maintains competitive accuracy in low-energy regions while reducing systematic bias in barrier heights, yielding a more balanced representation of torsional energy landscapes relevant for conformational sampling and transition kinetics.

Overall, these findings suggest that our hybrid design of ResFF ensures both the accuracy and generalizability for the torsional profile. As a result, ResFF could serve as a reliable tool for regular molecular simulation tasks, including geometry optimization and molecular dynamics, where capturing torsional energy details is especially important.

### ResFF captures intermolecular interactions with high accuracy

Accurate description of physical and biological processes, such as dimerization, protein-ligand binding, and crystallization, requires reliable modeling of intermolecular interactions. To assess ResFF’s capability in this regard, we evaluated its performance on the well-established S66×8 benchmark dataset, which comprises 528 dimers to test whether it can predict intermolecular energies with confidence. To ensure consistency of the QM level of theory, we also recomputed the single-point energies of S66×8 under ωB97M-D3(BJ)/def2-TZVPPD.

**Fig. 4:**
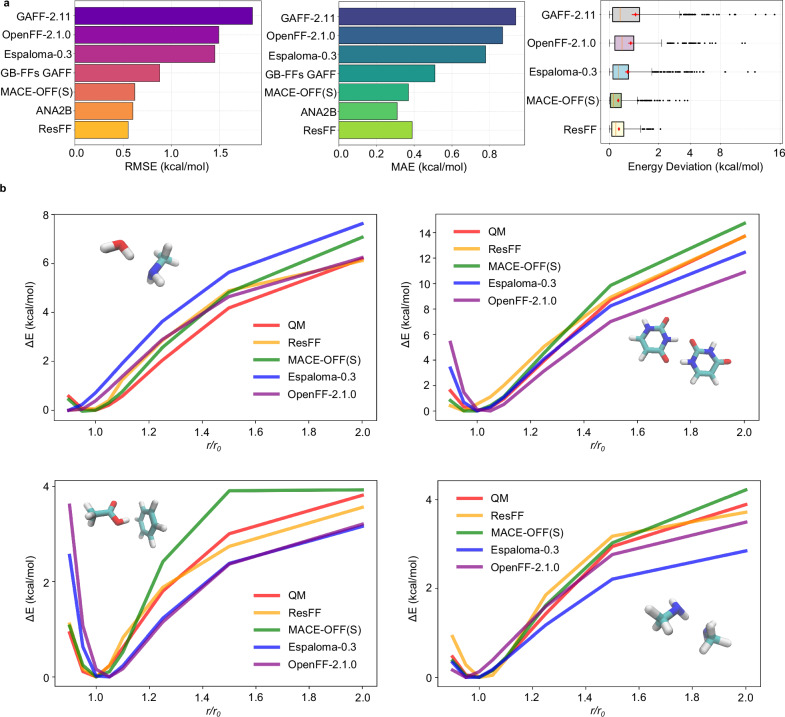
Performance of different methods on the S66×8 dataset. **a** RMSE, MAE, and energy deviation distributions. The mean energy deviation is marked by a red diamond, and the median is represented in an orange solid line. **b** Examples of predicted dimer interaction energy profiles compared with QM references. The horizontal axis shows the intermolecular distance ratio *r/r*_*0*_ (where *r* is the distance between monomers and *r*_*0*_ is their equilibrium separation), ranging from 0.9 to 2.0. The vertical axis denotes relative energies normalized to the method’s minimum energy points. Source data are provided as a Source Data file.

As shown in Table 3 and Fig. 4a, ResFF demonstrates exceptional accuracy with an RMSE of 0.55 kcal/mol, exceeding all compared force fields. As anticipated, although both methods adopt an end-to-end training framework, GB-FFs GAFF lags behind ResFF by approximately 24% (0.51 kcal/mol vs. 0.39 kcal/mol) in accuracy. This difference likely arises because the fully rigid functional forms in GB-FFs GAFF restrict its neural networks to fully capture the complex interactions revealed by the QM method. In contrast, ResFF’s hybrid architecture, combining physical baselines with flexible residual learning, enables precise modeling of such interactions.

**Table 3:** RMSE (kcal/mol), MAE (kcal/mol), Pearson correlation coefficient r, and coefficient of determination R^2^ of different methods on the S66×8 dataset

Methods	RMSE (kcal/mol)	MAE (kcal/mol)	Pearson’s r	R^2^
OpenFF-2.1.0	1.49	0.87	0.793	/
GAFF-2.11	1.84	0.94	/	/
GB-FFs GAFF	0.88	0.51	/	/
Espaloma-0.3	1.39	0.74	0.806	/
MACE-OFF(S)	0.62	0.37	**0.975**	0.910
ANA2B	0.60	**0.31**	/	/
ResFF	**0.55**	0.39	0.970	**0.929**

Best results are in bold, and second-best results are underlined. Results for GAFF-2.11 and GB-FFs GAFF are collected from Chen et al.^50^. Espaloma-0.3 and MACE-OFF(S) results are generated using published model weights. ANA2B result is collected from Thürlemann et al.^53^. Noted that S66×8 was used as an early-stopping criterion during the ANA2B training stage.

We further illustrate examples of dimer energy profiles for representative methods in Fig. 4b and Supplementary Fig. 3. Figure 4b presents four typical interaction motifs, where ResFF’s predicted interaction energies closely match QM references across all tested intermolecular distances. This aligns with the design principle outlined in Fig. 1c: by integrating physics-based constraints with data-driven corrections, ResFF balances accuracy within the training distribution and stability across unseen configurations. OpenFF-2.1.0 captures the general trends observed in QM calculations but lacks accuracy in reproducing fine-grained energy variations. Espaloma-0.3 exhibits similar behavior; its intermolecular interactions primarily rely on OpenFF’s noncovalent terms, resulting in less accurate profiles.

A distinct pattern is observed for purely local neural network force fields. MACE-OFF(S) agrees well with QM near equilibrium distances but deteriorates at extended separations, reflecting the intrinsic locality of message-passing architectures. Expanding the spatial receptive field alleviates this limitation: scaling to MACE-OFF(L) reduces the RMSE to 0.31 kcal/mol (Supplementary Table 3), underscoring the importance of interaction range. Alternatively, explicitly embedding long-range physics also improves performance. MACELES-OFF, which incorporates an electrostatic module, achieves an RMSE of 0.41 kcal/mol, while ANA2B, integrating analytical electrostatic and dispersion terms, reaches 0.60 kcal/mol. These models directly encode known analytical physical forms into the potential energy surface.

In contrast, ResFF adopts a hybrid formulation that does not attempt to reconstruct analytical long-range electrostatics within a finite-cutoff neural network. Instead, it combines an MM baseline with equivariant residual refinement to correct systematic deficiencies in the chemically relevant near-equilibrium regime. The MM backbone provides a physically stable reference landscape, while the residual network refines environment-dependent interactions without requiring the inference of full long-range physics from local descriptors alone. Within this framework, ResFF achieves an RMSE of 0.55 kcal/mol on S66 × 8, maintaining close agreement with QM across the entire test range.

Collectively, these results indicate that intermolecular accuracy may be enhanced through extended receptive fields, explicit long-range modeling, or structured residual refinement. Rather than competing strategies, explicit physical embedding and hybrid residual correction represent complementary inductive bias choices for machine learning force field design.

### ResFF reliably reproduces energy minima on OpenFF Industry Benchmark

Accurate evaluation of energetics and geometries is fundamental for computational modeling of chemical and biological systems. An ideal force field should faithfully reproduce the same energy minima and conformations consistent with QM calculations. We therefore evaluated the alignment of ResFF-optimized geometries with QM-optimized references using the OpenFF Industry Benchmark, which contains 73301 drug-like molecules.

Following an approach analogous to Takaba et al.^49^, we focused on two key comparison aspects: agreement of conformer energies and agreement of force-field-optimized (FF-opt) geometries with QM data. Three metrics were employed for assessment: root-mean-square deviation (RMSD) of atomic positions, relative energy difference (ΔΔ*E*), and torsion fingerprint deviation (TFD)^72^. TFD quantifies the deviations of all torsion angles using a Gaussian-weighted function, serving as a supplementary measure to RMSD for evaluating torsional accuracy. As an alignment-free metric, TFD overcomes major limitations of RMSD, which depends on the size or number of rotatable bonds of the underlying molecules and lacks normalization^72^. The ΔΔ*E* between the force field (FF) and QM energy for the i-th conformer of a molecule is calculated as:

1 $$\Delta \Delta {E}_{i}=|\Delta {E}_{{{{\rm{FF}}}},i}-\Delta {E}_{{{{\rm{QM}}}},i}|$$

2 $$=|({E}_{{{{\rm{FF}}}},i}-{E}_{{{\rm{FF}}},0})-({E}_{{{{\rm{QM}}}},i}-{E}_{{{\rm{QM}}},0})|$$

where the 0-th conformer denotes the lowest-energy state at either the FF or QM level for a given molecule.

**Fig. 5:**
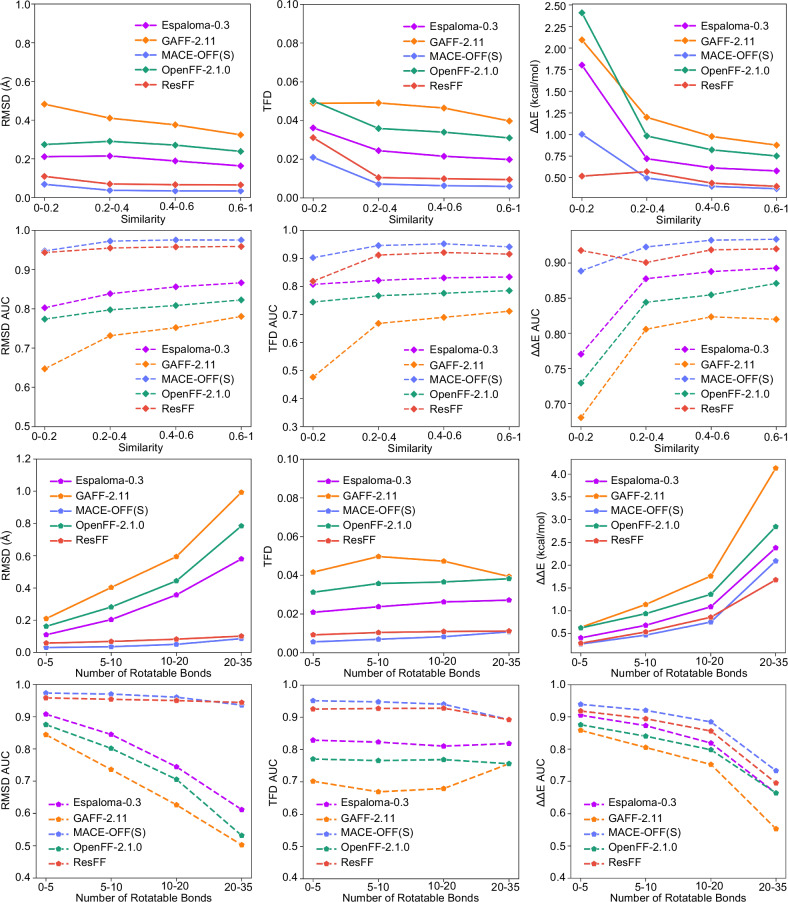
Metrics of different force fields on the OpenFF Industry Benchmark dataset. Median values and corresponding area under curves (AUCs) of root mean square deviation (RMSD, left), torsion fingerprint deviation (TFD, middle), and relative energy difference (ΔΔ*E*, right) for different force fields, evaluated as functions of molecular similarity to the SPICE dataset and the number of rotatable bonds. ResFF and MACE-OFF(S) are conducted using a BFGS optimizer with 0.1 kcal/mol·Å as convergence implemented in ASE. Results for GAFF-2.11, OpenFF-2.1.0, and Espaloma-0.3 are adapted from Takaba et al.^49^. Source data are provided as a Source Data file.

As shown in Fig. 5, to assess generalization behavior, we report the median values of RMSD, TFD, and ΔΔ*E*, as well as their corresponding AUC values with molecular similarity to the SPICE dataset. For molecules with high similarity to the training domain, ResFF outperforms classical MM force fields and Espaloma-0.3 on average, and achieves performance close to MACE-OFF(S). In practical applications, it is desirable that a model maintains robust performance even when extrapolating to less familiar regions of the extensive drug-like chemical space. In this regard, for molecules with low similarity scores below 0.2, ResFF exhibits the lowest ΔΔ*E* errors, indicating improved robustness when applied to structurally diverse and extrapolative scenarios.

Molecular flexibility further increases the difficulty of structure optimization due to the enlarged conformational search space. As shown in Fig. 5, the errors of classical MM force fields increase systematically with the number of rotatable bonds, whereas ResFF maintains performance comparable to MACE-OFF(S) models. For highly flexible molecules with more than 20 rotatable bonds, ResFF yields lower ΔΔ*E* values relative to QM references, while preserving low RMSD and TFD, indicating accurate reproduction of both equilibrium geometries and relative conformer energetics in challenging cases.

Overall, within SPICE-like chemical space, ResFF remains competitive with scale-matched local NNFFs, while in more extrapolative regimes (lower SPICE similarity and higher molecular flexibility) it exhibits consistently improved robustness. This provides confidence for the more challenging molecular dynamics simulations presented in the next section, where accurate energy representations are essential for capturing complex conformational dynamics and intermolecular interactions in realistic biological contexts.

### ResFF enables efficient and robust molecular dynamics simulations

A central application of force fields is to facilitate fast and stable molecular dynamics simulations, which are essential for predicting dynamic processes and thermodynamic properties in conformational sampling and protein-ligand binding studies. Classical molecular force fields often suffer from suboptimal accuracy and transferability due to the limitations of their functional forms and handcrafted parameterization. While NNFFs have emerged as a promising alternative for accurate energy and force prediction, MD simulations driven by NNFFs have been reported to exhibit instability due to unphysical behavior^43–45,52,73^. To evaluate ResFF’s suitability for MD simulations, we assessed its performance across multiple real-world biomolecular systems.

We first evaluated the performance of ResFF on a biological fragment, the tetrapeptide AcAla_3_NMe from the MD22 dataset^18^. We conducted a 1-nanosecond (ns) metadynamics simulation at 500 K with 1 femtosecond (fs) timestep in explicit solvent. It is noted that only the intramolecular interactions of the tetrapeptide are treated by ResFF, while the rest of the system is described using conventional MMFFs, providing a straightforward implementation as reported in refs. ^74,75^.

**Fig. 6:**
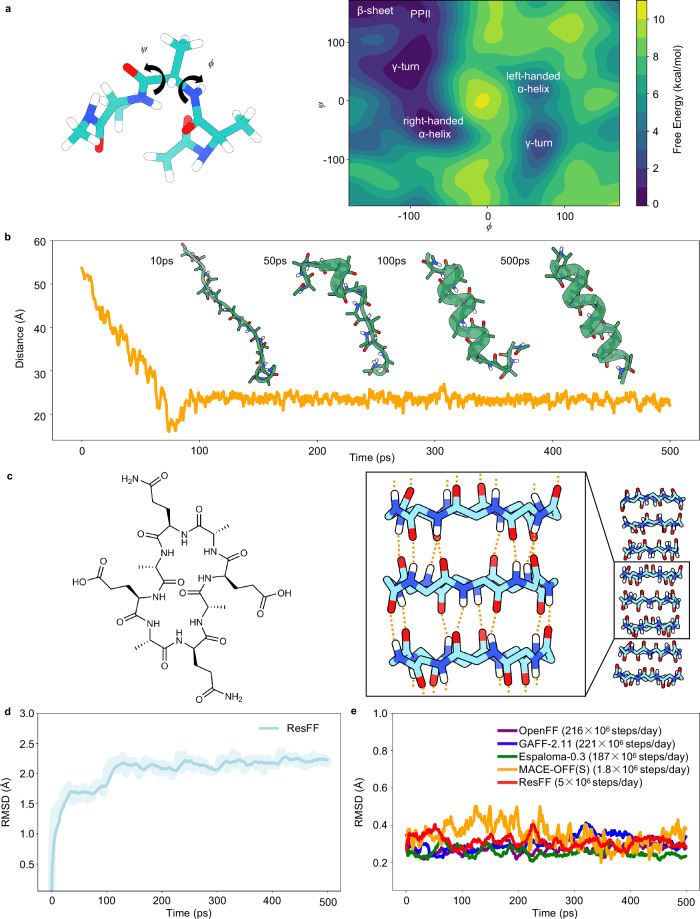
Molecular simulation of tetrapeptide, polyalanine, cyclic peptide (CY) assembly and Tyk2 complex. **a** Free energy surface of the tetrapeptide generated by ResFF. **b** Polyalanine folding dynamics: time evolution of the distance between α-carbons at the N- and C-termini. Representative conformations at 10 ps, 50 ps, 100 ps, and 500 ps illustrate secondary structure formation. **c** Chemical structure of the cyclic α-alt(D,L)-peptide. Representative final snapshot of CY assembly of MD simulation is shown using backbone atoms only. The intermolecular hydrogen bonding network that stabilizes the tubular assembly is highlighted in an orange dashed line. **d** RMSD of CY assembly during the simulation. The shaded area shows RMSD fluctuations, while the solid line represents the RMSD smoothed using a 15-point moving average. **e** Tyk2 simulations: comparison of ligand RMSD relative to the initial structure between different force fields. Simulation speeds are indicated. Source data are provided as a Source Data file.

In terms of computational efficiency, ResFF achieves a simulation speed of 5 × 10⁶ steps/day on an 80GB A100 GPU. Figure 6a presents the free energy surface (FES) with Ramachandran maps of AcAla_3_NMe, which characterize the peptide’s secondary structural conformation. ResFF successfully sampled several minima: polyproline II (PPII)-type conformations (− 90° ≤ *ϕ* ≤ − 60° and 100° ≤ *ψ* ≤ 180°), anti-parallel β-sheet (*ϕ* ≤ − 120°, *ψ* ≥ 120°), right-handed α-helical structure (*ϕ* ≈ – 60°, *ψ* ≈ – 60°), left-handed α-helix (*ϕ* ≈ 60°, *ψ* ≈ 60°) and γ-turn (*ϕ* ≈ − 60°/ *ψ* ≈ 60°, *ϕ* ≈ 60°/ *ψ* ≈ – 60°), consistent with the representative conformation of alanine in experimental observations^76,77^. We additionally performed simulations using the AMBER force field for comparison, as shown in Supplementary Fig. 4. Although the γ-turn basin is less pronounced in the AMBER FES, such conformations are not unprecedented. The γ-turn, characterized by an intramolecular CO (*i*)-NH (*i* + 2) hydrogen bond, is known to be energetically accessible in small capped peptide systems depending on backbone flexibility and sampling conditions^78^.

These results demonstrate ResFF’s ability to efficiently and reliably explore small molecule conformational landscapes over nanosecond-scale simulations, providing proof-of-concept for its stability in small molecule modeling.

We further investigated the folding dynamics of polyalanine. As a well-studied system, the 15-residue polyalanine (Ala_15_) is known to spontaneously fold into a stable α-helix in vacuo, driven by extensive hydrogen bonding interactions and synergistic polarization effects^79^. Some conventional force fields have been reported to fail in accurately modeling Ala_15_ folding^80^, prompting us to evaluate whether ResFF—trained solely on small molecules—can generalize to larger biomolecular systems.

As shown in Fig. 6b, simulations were initiated with a fully extended Ala_15_ structure, with the distance between the α-carbons of N-terminal and C-terminal residues tracked throughout. Within ~ 100 picoseconds (ps), Ala_15_ adopted a stable α-helical secondary structure. The folding process began with a short-time transition state characterized by a wavy conformation with dihedral angles *ϕ* ≈ 0° and *ψ* ≈ 0°, which transiently appears before rapidly folding into more stable secondary helical structure^80^ as shown in Supplementary Fig. 5a; once the first helical turn formed, the remaining helical segments assembled rapidly—consistent with experimental observations^79^. This phenomenon is similar to the nucleation of helical structures in Ala_15_ that initiates peptide folding^81^, suggesting that ResFF captures key transition structures underlying folding dynamics. Supplementary Fig. 5b further illustrates changes in hydrogen bond counts during folding, confirming that α-helix stability is primarily governed by hydrogen bonding interactions.

These results demonstrate ResFF’s capacity to extrapolate to complex hydrogen bonding networks, highlighting its potential for modeling biologically realistic systems.

To further evaluate the applicability of ResFF to larger, out-of-distribution systems, we performed MD simulations on a cyclic peptide (CY) assembly containing 848 atoms, substantially larger than the molecules present in the training data. Compared to linear peptides, cyclic peptides are more rigid and exhibit desirable drug-like properties, including high affinity for protein surfaces, increased specificity, and enhanced enzyme stability^82^. Accurate modeling of such systems is therefore critical for the design of cyclic peptide therapeutics.

Following the work of Ghadiri et al., who designed and synthesized a cyclic α-alt(D,L)-peptide with the sequence of cyclo-[(L-Gln-D-Ala-L-Glu-D-Ala)_2_-] (Fig. 6c) and showed that it forms ordered tubular assemblies stabilized by inter-ring backbone hydrogen bonds^83,84^, we constructed an eight-ring stack using PyMOL, initializing the inter-ring spacing at 4.73 Å, consistent with prior structural and computational studies^85,86^. ResFF was used to model both intra- and inter-peptide interactions, while water was represented with the TIP3P model. A 500 ps MD simulation was performed at 300 K with a 1 fs time step.

During the simulation, the CY assembly remained structurally stable, with persistent inter-ring hydrogen bonding networks forming between neighboring peptide backbones. The all-atom RMSD and a representative final-frame snapshot (Fig. 6c, d) confirm structural integrity. The average inter-ring distance is 4.85 Å, closely matching the experimentally reported value of 4.73 Å^83,84^. The full inter-ring distance distribution in Supplementary Fig. 6 shows modest fluctuations consistent with a thermally equilibrated system.

These results demonstrate that ResFF, although trained primarily on relatively small molecules, can reliably reproduce physically reasonable structural features in significantly larger systems, highlighting its potential applicability for modeling supramolecular assemblies and other extended peptide-based structures.

To evaluate ResFF’s applicability to heterogeneous protein-ligand systems, we conducted simulations on Tyk2, a well-studied tyrosine protein kinase with significant therapeutic implications in inflammatory bowel disease (IBD). A 500 ps simulation was performed at 300 K in explicit solvent. Following the approach in previous studies^75,87^, we constructed a hybrid simulation system where ligand internal energetics were parameterized by ResFF or MACE-OFF(S), while the other components were modeled using traditional force fields. Each simulation setup was repeated three times to ensure reproducibility.

As shown in Fig. 6e, all force fields exhibit stable ligand dynamics over the simulation time, as reflected by comparable ligand RMSD profiles. The potential energy and temperature fluctuations in Supplementary Figs. 7 and 8 likewise show no signs of integration failures or abnormal temperature excursions. ResFF shows stable behavior similar to conventional molecular mechanics force fields. In particular, analysis of ligand bond-length distributions shows that ResFF produces narrow, physically reasonable distributions consistent with conventional molecular mechanics force fields (Supplementary Fig. 9). Overall, these results demonstrate that ResFF supports stable MD simulations across a wide range of system sizes and environments, from small molecules to CY assemblies and protein-ligand complexes, supporting its further applicability in drug discovery.

## Discussion

Achieving an optimal balance between accuracy and generalization remains a key challenge in molecular simulations. While classical force fields offer robust physical interpretability, they often struggle to capture complex noncovalent interactions, whereas NNFFs excel in accuracy but may lack generalizability. In this work, we introduce ResFF, a hybrid force field that leverages deep residual learning to integrate the strengths of both approaches. By retaining analytical MM terms, ResFF inherits the robust physical foundation and generalizability of MMFFs, while its residual neural network component dynamically corrects for residual but complex interactions that are difficult to describe analytically. We also designed an end-to-end training procedure, allowing the two components to complement each other and deliver the best possible performance. This synergistic design enables ResFF to address the trade-off between accuracy and generalization effectively.

The residual learning framework of ResFF allows the neural networks to focus on learning the difference (residual) between QM energies and predictions from the analytical base model. This approach not only reduces the learning complexity for the neural networks but also ensures that physical constraints from classical force fields are preserved. Although the total energy is expressed as a linear sum of MM and residual terms, the residual network introduces nonlinear corrections within its finite interaction cutoff. The formulation, therefore, remains locally nonlinear in representational capacity, while still constrained by the locality of the model architecture. As demonstrated on the Gen2-Opt and DES370K benchmarks, ResFF outperforms both traditional force fields and several widely used NNFFs in energy prediction accuracy, while maintaining broad generalizability to unseen molecular systems. Furthermore, ResFF accurately reproduces QM-derived torsional profiles, intermolecular interaction energies, and energy minima across diverse chemical environments, highlighting its reduced propensity for overfitting and enhanced transferability to complicated drug-like molecules. More importantly, in molecular dynamics simulations from small protein fragments to large biological assembly and protein-ligand complex, ResFF can produce stable and reasonable trajectories. These results demonstrate its robustness and practicality for heterogeneous biological systems, as well as its potential for future applications in accurate binding free energy calculations and for elucidating structural mechanisms in drug discovery. Finally, as shown in Supplementary Fig. 10, we benchmarked the computational performance of ResFF for systems ranging from 42 atoms up to approximately 7962 atoms, using OpenMM on a single A100 GPU. The current implementation exhibits scaling with a measured slope of 0.024 ms/atom/step over the tested range. For a protein system of roughly 1000 atoms, ResFF attains a throughput of approximately 2.5 × 10⁶ steps/day, which is similar to that of the MACE-OFF(S). While the present implementation is primarily optimized for small to medium-sized systems, these results provide a clear baseline for future development. We expect that substantial improvements are feasible through engineering optimizations, such as C++ acceleration and multiple time step (MTS) integration, where the NNFF is evaluated with a larger outer time step, while the more efficient MMFF is applied at every inner time step to accelerate the MD simulation^35^, which are part of our ongoing and future work.

Despite these encouraging results, certain limitations remain. First, our reliance on conventional MM covalent terms may still cap the attainable accuracy, since the choice of current covalent terms is inherently restricted. Future work could refine how functional forms and neural network corrections could be best allocated. Second, while ResFF is currently best suited for drug-like molecules within the chemical space of SPICE, its applicability is primarily limited to systems dominated by local interactions. Because the present architecture does not explicitly incorporate long-range Coulomb or polarization terms, retraining or fine-tuning on task-specific data is recommended for systems in which long-range electrostatics or coordination effects play a central role, such as highly charged species, transition-metal complexes, or extended biopolymers. As an additional transferability assessment, we performed condensed-phase benchmarks on simple organic liquids (Supplementary Fig. 11). These results suggest reasonable stability for local packing-dominated properties, but also indicate that ResFF is not yet optimized for bulk thermodynamic quantities governed by long-range electrostatics and dispersion. Applications to periodic liquids, materials, or other long-range-dominated systems, therefore, require additional validation and future incorporation of explicit long-range treatments. Finally, the hybrid nature of ResFF requires substantial engineering work to integrate with existing molecular dynamics software for advanced alchemical free energy calculations. Nevertheless, emerging interfaces in tools such as ASE^88,89^, OpenMM^47^, and LAMMPS^90^ offer promising pathways for streamlined implementation.

Accurate and generalizable molecular simulations could drive deeper insights into biological mechanisms and facilitate drug discoveries. Therefore, it has long been a fundamental goal of computational chemistry. By presenting a residual-learning route for force field development, ResFF offers a compelling alternative that capitalizes on both physics-based and data-driven strengths. With ongoing improvements in computational power and software engineering, we anticipate that ResFF could evolve into an effective tool for large-scale molecular simulations, ultimately contributing insights to biological research and drug discovery.

## Methods

### Details of the ResFF architecture

ResFF comprises two core components: an MM module and a residual module. The MM module establishes a physics-informed energy baseline, while the residual module refines predictions via data-driven corrections. A shared feature embedding layer provides a unified molecular representation for both modules.

The MM module predicts force field parameters for explicit covalent terms (bond stretches, angle bends, and torsions), as covalent terms are well designed to capture the dominant covalent contributions and can be reliably fitted to abundant QM training data without requiring costly experimental measurements. In this way, the module encodes the primary covalent interactions to form a physically grounded energy scaffold. The residual module complements this by modeling pairwise atomic interactions, dynamically updating latent atomic representations via a modified multi-head attention mechanism to capture context-dependent interatomic effects. The residual energy predicted by this module serves as an inexpensive correction to the approximately fitted covalent interactions, rather than representing strictly defined noncovalent terms as in conventional MMFFs; hence, we refer to it as “residual”. Finally, an output module employs gated equivariant modules to predict scalar atom-wise energies, which are aggregated to yield the total molecular residual energy.

The total energy of a system is computed as the sum of the energies from the MM module and the residual module. This residual learning framework enables ResFF to efficiently integrate the dominance of covalent interactions with the flexibility required to model noncovalent effects, supporting accurate and generalizable molecular simulations.

ResFF begins molecular representation by converting SMILES into a topology graph, where atoms and covalent bonds are encoded as nodes and edges, respectively. Atomic features—including element type, hybridization state, atomic degree, aromaticity, atomic mass, and ring size—are extracted via RDKit and concatenated into continuous embeddings. This design eliminates reliance on discrete atom typing rules, instead providing a data-driven molecular representation that aligns with the residual learning framework. Such flexibility in feature encoding enables subsequent modules to effectively capture both dominant covalent interactions and complex residual corrections.

The MM module leverages a 2D-GNN for message passing, allowing atoms to aggregate local neighborhood information and update their feature representations^91,92^. To enforce permutation invariance—ensuring chemically equivalent atoms yield identical bond, angle, and torsion parameters—Janossy pooling^93^ is used to generate unified embeddings for bond, angle, and torsion terms. These embeddings are fed into feed-forward neural networks to predict corresponding force field parameters: equilibrium bond length (${r}_{0}$) and force constants (${K}_{r}$) for bonds; equilibrium angles (${\theta }_{0}$) and force constants (${K}_{\theta }$) for angles; force constants (${K}_{\varphi,{{\rm{proper}}}}$) for torsions; and force constants (${K}_{\varphi,{{\rm{improper}}}}$) for improper torsions. The improper torsion term is introduced to model out-of-plane distortions and maintain planarity, thereby improving the representation of inversion barriers. These parameters remain fixed during simulations.

The energy ${E}_{{{\rm{MM}}}}(R)$ (R represents atomic coordinates) of MM module is computed as the sum of bond, angle, and torsion terms:

3 $${E}_{{{\rm{MM}}}}(R)={E}_{{{\rm{bond}}}}+{E}_{{{\rm{angle}}}}+{E}_{{{{\rm{proper}}}} \, {{{\rm{torsion}}}}}+{E}_{{{{\rm{impropr}}}}\,{{{\rm{torsion}}}}}$$

where:

4 $${{E}}_{{{\rm{bond}}}}={\sum}_{{{\rm{bond}}}}\frac{1}{2}{{K}}_{{r}}{\left({{r}}_{{i},{j}}-{{r}}_{0}\right)}^{2}$$

5 $${{E}}_{{{\rm{angle}}}}={\sum}_{{{\rm{angle}}}}\frac{1}{2}{{K}}_{\theta }{({\theta }_{{i},{j},{k}}-{\theta }_{0})}^{2}$$

6 $${E}_{{{{\rm{proper}}}}\,\,{{{\rm{torsion}}}}}={\sum}_{{{{\rm{proper}}}}\,{{{\rm{torsion}}}}}{\sum}_{n}{K}_{\varphi,{{{\rm{proper}}}},n}\left[1+\cos (n{\varphi }_{{{{\rm{proper}}}},i,j,k,l}-{\varphi }_{0})\right]$$

7 $${E}_{{{{\rm{improper}}}}\,\,{{{\rm{torsion}}}}}={\sum}_{{{{\rm{improper}}}}\,{{{\rm{torsion}}}}} {\sum}_{n}{K}_{\varphi,{{{\rm{improper}}}},n}\left[1+\cos (n{\varphi }_{{{{\rm{improper}}}},i,j,k,l}-{\varphi }_{0})\right]$$

Here, ${r}_{i,j}$, ${\theta }_{i,j,k}$, ${\varphi }_{{{\rm{proper}}},i,j,k,l}$ and ${\varphi }_{{{\rm{improper}}},i,j,k,l}$ denote the bond length, bond angle, and proper torsion angle and improper torsion angle derived from atomic coordinates, respectively; ${K}_{b}$, ${r}_{0}$, ${K}_{\theta }$, ${\theta }_{0}$, ${K}_{\varphi,{{\rm{proper}}},n}$ and ${K}_{\varphi,{{\rm{improper}}},n}$ are the parameters predicted for each valence term. Torsion periodicities n are set to 1 through 6, with phase angle ${\varphi }_{0}$ fixed at 0.

Analogous to the “identity mapping” in ResNet^62^, this module provides a stable physics-based baseline for molecular energy, consistently capturing dominant covalent interactions and forming the foundation for residual learning.

To model residual interactions, we employ the Equivariant Transformer (ET)^30,31^ architecture—an equivariant neural network based on vector representations. Compared to models relying on irreducible representations, ET reduces computational overhead significantly while maintaining robust performance. Its role is to predict residual molecular energy from the atomic numbers and positions, following a structured workflow outlined below.

First, an embedding layer transforms atomic numbers and local atomic environments into dense scalar and vector feature vectors for each atom. Specifically, for each atom *i*, its 1-hop neighbors *j* and pairwise distance ${d}_{{ij}}$ (within a predefined cutoff) are integrated to generate initial scalar node embeddings ${{{h}}}_{i}^{0}$ and edge embeddings ${f}_{{ij}}^{0}$.

8 $${{{h}}}_{{i}}^{0},{{f}}_{{ij}}^{0}={{{\rm{Embed}}}}\left({{z}}_{{i}},{\left\{{{z}}_{{j}},{{d}}_{{ij}}\right\}}_{{j}\in 1-{{{\rm{hop}}}} \,{{{\rm{neighbors}}}}}\right)$$

where ${z}_{i}$ denotes the atomic number of atom *i*.

Next, a sequence of update layers captures pairwise interactions using a modified multi-head attention mechanism, enabling progressive refinement of both scalar and vector representations.

This process relies on message passing and aggregation, as described by Eqs. 9–12. Scalar messages ${m}_{i}^{l}$—dependent on node features ${{{h}}}_{i}^{l},{{{h}}}_{j}^{l}$, and edge embeddings ${f}_{{ij}}^{l}$—are transmitted from neighbor *j* to atom *i* through a message function ${f}_{m}^{l}$. Concurrently, vector messages ${{{{\bf{m}}}}}_{i}^{l}$ are constructed using scalar message ${m}_{{ij}}^{l}$, relative position vectors ${{{{\bf{r}}}}}_{{ij}}$, and neighbor vector feature ${{{{\bf{v}}}}}_{j}^{l}$. Let ${N}_{i}$ represent the number of 1-hop neighbors of atom i; update functions then refine the features:

9 $${{{h}}}_{{i}}^{{{l}}+1}={{f}}_{{u}}^{{l}}({{{h}}}_{{i}}^{{l}},{{m}}_{{i}}^{{l}},\left\langle {{{{\bf{v}}}}}_{{i}}^{{l}},{{{{\bf{v}}}}}_{{{i}}}^{{l}}\right\rangle )$$

10 $${{{{\bf{v}}}}}_{i}^{l+1}={f}_{u}^{l}({{{{\bf{v}}}}}_{i}^{l},{m}_{i}^{l},{{{{\bf{m}}}}}_{i}^{l})$$

11 $${{m}}_{{i}}^{{l}}={\sum}_{{{j}}\in {{N}}_{{i}}}{{{f}}_{{m}}^{{l}}}({{\mbox{h}}}_{{i}}^{{l}},{{\mbox{h}}}_{{j}}^{{l}},{{\mbox{f}}}_{{ij}}^{{l}})$$

12 $${{{{\bf{m}}}}}_{{{i}}}^{{{l}}}={\sum}_{{{{j}}}\in {{{N}}}_{{{i}}}}{{{{f}}}_{{{m}}}^{{{l}}}}({{{m}}}_{{{ij}}}^{{{l}}},{{{{\bf{r}}}}}_{{{ij}}},{{{{\bf{v}}}}}_{{{j}}}^{{{l}}})$$

where scalar embedding ${{{h}}}_{i}^{l+1}$ is updated using the aggregated scalar message ${f}_{u}^{l}$, and the vector feature ${{{{\bf{v}}}}}_{i}^{l+1}$ is updated by combining scalar and vector messages via an update function ${f}_{u}^{l}$.

Notably, to preserve equivariance (ensuring consistent behavior under global rotations/translations), only linear operations are applied to vector features during message passing and updates.

Finally, layer normalization is applied, followed by an output network using gated equivariant blocks to generate scalar atom-wise predictions. These are aggregated to yield the residual energy ${E}_{{{\rm{residual}}}}$:

13 $${E}_{{{\rm{residual}}}}={\sum}_{i=1}^{N}{{{h}}}_{i}^{L}$$

where ${{{h}}}_{i}^{L}$ denotes the final scalar embedding of atom *i* from the last GNN layer L, and *N* is the total number of atoms in the molecule. The total molecular energy is computed as the sum of residual energy and the ${E}_{{{\rm{MM}}}}$ from the MM module, completing the energy prediction framework.

### Data for training and evaluation

We use the SPICE^21^ dataset for training ResFF in more practical scenario, whose composition is detailed in Table 4. The dataset contains QM energies and forces computed at the ωB97M-D3(BJ)/def2-TZVPPD level using Psi4^94^, providing high-fidelity reference data. Geometries in the SPICE dataset were generated via classical force field-driven MD simulations, with a focus on sampling maximally distinct conformations to cover diverse molecular configurations. Following the approach of Wang et al.^48^, we excluded configurations with energies more than 0.1 Hartree (62.5 kcal/mol) above the minimum and removed ion pairs to ensure physical validity. Unique molecules were then partitioned into training, validation, and testing sets at an 8:1:1 ratio.

**Table 4:** Summary of the datasets used to train and evaluate the model

Training Data					
Datasets		Molecules	Conformations	Atoms	Elements
SPICE^21^	Dipeptides	677	33663	26–60	H, C, N, O, S
	Solvated amino acids	26	1300	76–96	H, C, N, O, S
	DES370K dimers	3095	326978	2–34	H, C, N, O, F, P, S, Cl, Br, I
	DES370K monomers	374	18700	2–33	H, C, N, O, F, P, S, Cl, Br, I
	PubChem	14601	707449	3–50	H, C, N, O, F, P, S, Cl, Br, I
Gen2 Optimization^63^		1024	244989	11–76	H, C, N, O, F, Si, P, S, Cl, Br, I
Evaluation Data					
Dataset		Mols	Conformations	Atoms	Elements
TorsionNet-500^65^		500	12000	13–37	H, C, N, O, F, S, Cl
Torsion Scan^66^		62	2232	10–23	H, C, N, O, F, S
S66×8^67^		66	528	6–34	H, C, N, O
OpenFF Industry Benchmark^68^		9728	73301	2–82	H, C, N, O, F, P, S, Cl, Br

To assess extrapolation capability—defined as performance on unseen molecular systems—we conducted parallel experiments comparing ResFF with classical MMFFs and NNFFs. Two benchmark datasets were used: the Gen2 Optimization (Gen2-Opt) dataset and the DES370K dataset. Gen2-Opt comprises drug-like molecules used in parameterizing OpenFF 1.2.0^4^, with geometries optimized at the B3LYP-D3(BJ)/DZVP level. DES370K, a subset of SPICE, contains diverse molecular dimers. These validation benchmarks enable direct comparison of ResFF with classical MMFFs and NNFFs, highlighting how its residual learning architecture enhances generalization to previously unseen molecular systems.

Four benchmark datasets were used to comprehensively assess ResFF performance across key molecular properties:

TorsionNet-500^65^: Contains torsion drive calculations for 500 diverse molecules spanning pharmaceutical chemical space (including acyclic, aryl, and biaryl motifs). Single-point energies of optimized conformations, computed at the B3LYP/6-31G(d) level, provides a benchmark for evaluating torsional potential energy surfaces—critical for accurate molecular conformational sampling in drug discovery.

Torsion Scan^66^: Includes 62 drug-like molecular fragments (e.g., aryl, aryl-amide, biaryl) with dihedral angles systematically varied from − 170° to 170° in 10° increments. Single-point energies at the CCSD(T)/CBS level enable rigorous assessment of torsional barriers, which are essential for characterizing rotational dynamics in functional groups common to bioactive compounds.

S66×8^67^: Features 528 dimer configurations from 66 representative molecular systems, sampled at 8 intermolecular separations (0.9–2.0 × equilibrium distance). Interaction energies calculated at the CCSD(T)/CBS level cover diverse noncovalent interactions (hydrogen bonding, dispersion, and mixed electrostatic/dispersion effects), offering a comprehensive test of force field accuracy in modeling weak intermolecular forces.

OpenFF Industry Benchmark^68^: Comprises 73,301 drug-like molecules with QM-optimized geometries (B3LYP-D3BJ/DZVP), serving as a large-scale validation set for reproducing energy minima and molecular geometries. Sourced from pharmaceutical and chemical industries, it ensures relevance to real-world drug design applications.

### Training objective and three-stage optimization strategy

ResFF employs a weighted multi-target mean squared error (MSE) loss function to minimize discrepancies between predictions and reference data for both total energy and atomic force. The total loss is formalized as:

14 $${{{\rm{Loss}}}}={{{\rm{Loss}}}}_{{{\rm{E}}}}+{\lambda {{\rm{Loss}}}}_{{{\rm{F}}}}$$

where λ balances the contributions of energy and force losses (set to 1 during training, corresponding to an equal ratio of force loss to energy loss). The energy ${{{{\rm{Loss}}}}}_{{\rm{E}}}$ and force ${{{\rm{Loss}}}}_{{\rm{F}}}$ are defined as:

15 $${{{\rm{Loss}}}}_{{{\rm{E}}}}={{||}{E}_{{{\rm{pre}}}}-{E}_{{{\rm{ref}}}}{||}}^{2}$$

16 $${{{\rm{Loss}}}}_{{{\rm{F}}}}=\frac{1}{{N}}{\sum}_{{n}=1}^{{N}}\frac{1}{3{{N}}_{{a}}}{\sum}_{{i}=1}^{{{N}}_{{a}}} {\sum}_{\alpha={x},{y},{z}}{{||}{{{{\bf{F}}}}}_{{{\rm{pre}}}}-{{{{\bf{F}}}}}_{{{\rm{ref}}}}{||}}^{2}$$

17 $${E}_{{{\rm{pre}}}}={E}_{{{\rm{MM}}}}+{\beta }{E}_{{{\rm{residual}}}}$$

18 $${{{{\bf{F}}}}}_{{{\rm{pre}}}}=-\nabla {E}_{{{\rm{pre}}}}$$

Here, ${E}_{{{\rm{pre}}}}$ denotes ResFF’s predicted total energy, where ${E}_{{{{\rm{MM}}}}}$ and ${E}_{{{{\rm{residual}}}}}$ are the outputs from the MM and residual modules, respectively, and ${\beta }$ weights the contribution of residual energy. ${E}_{{{{\rm{ref}}}}}$ is the reference QM energy. For atomic forces, ${{{{\bf{F}}}}}_{{{\rm{pre}}}}$ represents ResFF’s predicted force—calculated as the negative gradient of the total energy with respect to atomic coordinates (where ∇ denotes the gradient operator)—and ${{{{\bf{F}}}}}_{{{\rm{ref}}}}$ is the reference QM force. N and ${N}_{a}$ denotes the number of molecules in the dataset and the number of atoms in each configuration. The units of the ${{{\rm{Loss}}}}_{{{\rm{E}}}}$ and ${{{\rm{Loss}}}}_{{{\rm{F}}}}$ are in (kcal/mol)^2^ and (kcal/mol·Å)^2^, respectively.

To leverage synergies between the MM and residual modules, training proceeds in three sequential stages, each designed to enhance the efficiency of residual learning, as described below:

Stage 1: MM Module Optimization. The residual module is frozen (${\beta }$ = 0) to isolate training of the MM module. This stage prioritizes learning dominant covalent interactions (bond stretches, angle bends, torsions), establishing a stable physics-informed baseline—analogous to the “identity mapping” in ResNet^62^. By focusing on MM terms, the framework ensures that subsequent residual corrections target noncovalent deviations rather than re-learning fundamental covalent physics.

Stage 2: Residual Module Specialization. With the MM baseline converged, its parameters are fixed, and the residual module is trained independently (${\beta }$ = 1). Here, the equivariant neural network learns to model subtle noncovalent interactions (e.g., hydrogen bonding, dispersion), capturing energy contributions beyond the MM baseline. This stage mirrors “residual mapping” in deep learning, where the neural network refines predictions by learning deviations from the established baseline.

Stage 3: Joint Optimization. The final stage alternates between fine-tuning the MM and residual modules over two cycles, with *β* maintained at 1. This joint optimization ensures the modules evolve synergistically: the MM module provides interpretable physical constraints, while the residual module delivers adaptive noncovalent corrections.

This three-stage paradigm embodies the core of residual learning: decomposing energy prediction into a physics-informed baseline and data-driven corrections. The further energy decomposition result and visualizations presented in Supplementary Figs. 12 and 13 support the rationale behind the model design. The MM terms contribute the most to the total energy predicted by ResFF, while the residual component accounts for a relatively small portion, consistent with previous findings that intermolecular interactions are several orders of magnitude weaker than covalent bonds^58^.

Ablation experiments are further conducted to validate the effectiveness of the residual module and the necessity of the three-stage training strategy. As shown in Supplementary Table 4, although the residual term contributes only a small fraction of the total molecular energy, it plays a crucial role in improving accuracy. Moreover, joint optimization further refines the balance between MM and residual components, leading to optimal performance. By separating covalent dominance from noncovalent complexity, ResFF avoids overfitting pitfalls of pure NNFFs while surpassing classical MMFFs in describing complex interactions.

### Metrics

Four metrics are used to assess all the methods: MAE, RMSE, Pearson correlation coefficient (r), and coefficient of determination (R^2^). These metrics are calculated as follows:

19 $${{{\rm{MAE}}}}=\frac{1}{{N}} {\sum }_{{i}}^{{N}}|{{E}}_{{{\rm{FF}}}}-{{E}}_{{{\rm{QM}}}}|$$

20 $${{{\rm{RMSE}}}}=\sqrt{\frac{1}{{N}}{\sum }_{{i}}^{{N}}{({{E}}_{{{\rm{FF}}}}-{{E}}_{{{\rm{QM}}}})}^{2}}$$

21 $${{r}}=\frac{{\sum }_{{i}}^{{N}}({{E}}_{{{\rm{FF}}}}-\overline{{{E}}_{{{\rm{FF}}}}})({{E}}_{{{\rm{QM}}}}-\overline{{{E}}_{{{\rm{QM}}}}})}{\sqrt{{\sum }_{{i}}^{{N}}{({{E}}_{{{\rm{FF}}}}-\overline{{{E}}_{{{\rm{FF}}}}})}^{2}{({{E}}_{{{\rm{QM}}}}-\overline{{{E}}_{{{\rm{QM}}}}})}^{2}}}$$

22 $${{\mbox{R}}}^{2}=1-\frac{{\sum }_{{i}}^{{N}}{({{E}}_{{{\rm{QM}}}}-{{E}}_{{{\rm{FF}}}})}^{2}}{{\sum }_{{i}}^{{N}}{({{E}}_{{{\rm{QM}}}}-\overline{{{E}}_{{{\rm{QM}}}}})}^{2}}$$

where ${E}_{{{\rm{FF}}}}$ and ${E}_{{{\rm{QM}}}}$ represent the energy predicted by the FF and the QM reference energy, respectively. $\overline{{E}_{{{\rm{FF}}}}}$ and $\overline{{E}_{{{\rm{QM}}}}}$ denote the averages of the predicted energy by the force field and the QM, respectively. N is the number of conformations. All statistics are computed with the ${E}_{{{\rm{FF}}}}$ and ${E}_{{{\rm{QM}}}}$ centered to have a zero mean for each molecule.

### Experimental settings

When training on the SPICE dataset, we utilized GraphSAGE as the GNN for the MM module, following the approach of Wang et al.^48^. The MM module was configured with 3 GraphSAGE layers, each containing 512 units, and 4 GraphSAGE layers with 512 units for the symmetry-preserving pooling and readout stages. For the residual module, we employed the ET^30,31^ model, configured with 3 interaction layers, 80 hidden channels, 64 non-trainable radial basis functions, 8 attention heads, and a cutoff of 10 Å. For training, we used a batch size of 64, an initial learning rate of 1e-4, and the Adam optimizer with PyTorch’s default parameters. MSE loss was weighted energy and forces, with both weights equal to 1.0. The learning rate decayed using an on-plateau scheduler, based on the total weighted validation MSE losses, with a patience of 20 and a decay factor of 0.8. Training terminates when reaching early stopping training rounds of 50 or validation loss stagnates for 20 consecutive epochs. When training on the Gen2-Opt and DES370K dataset, all the units in the MM module were reduced to 128 and the forces weight was set to 0. The training was conducted on a single NVIDIA A100 GPU.

For a parallel comparison of the Gen2-Opt and DES370K, we used the MACE-S default training settings with a batch size of 64 provided in https://github.com/ACEsuit/mace. For NequIP, we used the default training settings in https://github.com/mir-group/nequip/blob/main/configs/examples.yaml with ${r}_{\max }$ of 6.0, number of layers of 4, ${l}_{\max }$ to 1, number of features of 32 and batch size of 128. It should be noted that we trained NequIP, MACE-OFF(S), and ResFF solely on energies due to the high computational cost. This choice may impact their performance, as force field models are often trained jointly on energies and forces to achieve optimal accuracy. Espaloma-0.3 was trained on about 1 million conformations derived from a curated subset of the SPICE dataset, including optimized geometries and torsion-drive datasets from OpenFF 1.x and 2.x, along with additional peptide and RNA data. The weights of Espaloma-0.3 were used without fine-tuning for inference, which is available in https://github.com/choderalab/espaloma/releases/download/0.3.2/espaloma-0.3.2.pt. The results of OpenFF and GAFF were generated using OpenMM. Snapshots with energies more than 0.1 Hartree (62.5 kcal/mol) above the minimum were filtered from the Gen2-Opt, as done in previous research^48^. For the results of OpenFF, GAFF, and Espaloma-0.3 in DES370K, predicted energies exceeding this threshold were likewise excluded to mitigate large deviations in intermolecular interactions, given that MMFFs were trained on near-equilibrium conformations.

For evaluation of the TorsionNet-500, Torsion Scan and S66×8, our ResFF was trained on 0.8 million conformations collected from the SPICE dataset, without any additional torsion scan refinement procedures. We used the weights of MACE-OFF(S) from https://github.com/ACEsuit/mace/tree/main. MACE-OFF(S) was trained on the SPICE dataset, further supplemented with molecules from the QMugs dataset and water clusters, enhancing its applicability to larger and solvent systems. The total training set comprised approximately 0.9 million conformations. For Espaloma-0.3, the weight was from https://github.com/choderalab/espaloma/releases/download/0.3.2/espaloma-0.3.2.pt without fine-tuning. The results of ANI-2x, TorsionNet and GB-FFs GAFF were collected from their corresponding literature. ANI-2x is an NNFF trained on approximately 8.9 million molecular conformations, incorporating a torsion refinement step to improve its accuracy in capturing torsion energy profiles. TorsionNet is a specialized neural network model designed for predicting torsional energies, trained on 1.2 million conformations generated through systematic torsion scan procedures. GB-FFs GAFF was initially pretrained on the ANI-1 dataset and subsequently fine-tuned on the SPICE dataset, totaling around 14 million training conformations.

All MD simulations were performed in OpenMM version 8.1.2 with a timestep of 1 fs on a single NVIDIA A100 GPU. In explicit solvent simulations, water molecules were modeled using the TIP3P water model. For the benchmark systems, tetrapeptide metadynamics simulation was carried out in OpenMM at 500 K for 1 ns, using a bias factor of 10, a barrier height of 1 kJ/mol, and an update frequency of 100 steps to enhance sampling along the central backbone *ϕ* and *ψ* dihedral angles. Polyalanine folding dynamics was simulated in the gas phase at 300 K for 500 ps. Cyclic peptide assembly simulation was performed in explicit solvent at 300 K for 500 ps. Simulations of the Tyk2 protein-ligand system were conducted in explicit solvent at 300 K for 500 ps, with the protein described by the AMBER ff14SB, water modeled using TIP3P, and the ligand parameterized using ResFF or alternative molecular force fields. Finally, for the condensed-phase benchmarks (Supplementary Fig. 11), liquid boxes of 64 molecules were minimized by L-BFGS and simulated in the NPT ensemble at 298.15 K and 1 atm using a Langevin integrator, 1 fs timestep, and Monte Carlo barostat. Each trajectory was run for 300 ps, with the final 100 ps used for density and energy averaging. Enthalpies of vaporization ($\Delta {{\mbox{H}}}_{{{\rm{vap}}}}$) was calculated from gas- and liquid-phase energy differences with the standard thermal correction. More details of the condensed-phase benchmarks are provided in Supplementary Note 1.

## Supplementary information

Supplementary Information

Transparent Peer Review file

## Source data

Source Data

## Data Availability

The SPICE dataset is available at https://zenodo.org/records/8222043. The Gen2-Opt dataset is available at https://zenodo.org/records/8150601. The TorsionNet-500 dataset can be found in ref. ^65^. The Torsion Scan dataset can be found in ref. ^66^. The S66×8 dataset can be found in ref. ^67^. The OpenFF Industry Benchmark is available at https://github.com/openforcefield/qca-dataset-submission/tree/master/submissions/2021-06-04-OpenFF-Industry-Benchmark-Season-1-v1.1. Source data are provided in this paper.

## Code availability

The code, training utilities, and model weights required to reproduce the reported results have been released under an open-source license at Zenodo: 10.5281/zenodo.20484065 (ref. ^95^), and the GitHub repository is available at https://github.com/Ameki0/ResFF.
