# Supplementary material for: Deep residual learning for molecular force fields
Source: Nat Commun. 2026 Jun 30;17:8160. doi: 10.1038/s41467-026-74983-0 (PMC13458457; doi:10.1038/s41467-026-74983-0)
Supplement: Supplementary file 1 [file 41467_2026_74983_MOESM1_ESM.pdf]

Deep Residual Learning for Molecular Force Fields

Supplementary Information

Xinyu Jiang[1,2,#], Mingan Chen[1,3,4,#], Chuanlong Zeng[1,2], Duanhua Cao[1,5], Jie Yu[1,4,6], Runze Zhang[1,2,7], Zunyun Fu[8], Zhehuan Fan[1,2], Jiacheng Xiong[1], Xutong Li[1], Xiaomin Luo[1,2], Dan Teng[1,*], Mingyue Zheng[1,2,*]

[1]Drug Discovery and Design Center, State Key Laboratory of Drug Research, Shanghai Institute of Materia Medica, Chinese Academy of Sciences, 555 Zuchongzhi Road, Shanghai, 201203, China.

[2]University of Chinese Academy of Sciences, No. 19A Yuquan Road, Beijing, 100049, China.

[3]School of Physical Science and Technology, ShanghaiTech University, 393 Middle Huaxia Road, Shanghai, 201210, China.

[4]Lingang Laboratory, Shanghai, 200031, China.

[5]Innovation Institute for Artificial Intelligence in Medicine of Zhejiang University, College of Pharmaceutical Sciences, Zhejiang University, Hangzhou, Zhejiang, 310058, China.

[6]School of Information Science and Technology, ShanghaiTech University, 393 Middle Huaxia Road, Shanghai, 201210, China.

[7]Science for Life Laboratory, Department of Cell and Molecular Biology, Uppsala University, BMC, Uppsala, Sweden.

[8]iHuman Institute, ShanghaiTech University, Shanghai 201210, China.

[#]These authors contributed equally: Xinyu Jiang, Mingan Chen.

*Correspondence should be addressed to Mingyue Zheng. Tel: +86-021-68077843; Email: myzheng@simm.ac.cn.

*Correspondence may also be addressed to Dan Teng. Tel: +86-021-68077844; Email: tengdan@simm.ac.cn.

Contents of this file

Supplementary Figures 1 to 13 3
Supplementary Tables 1 to 4 10
Supplementary Note 1 12
Supplementary References 14

Supplementary Figures 1 to 13

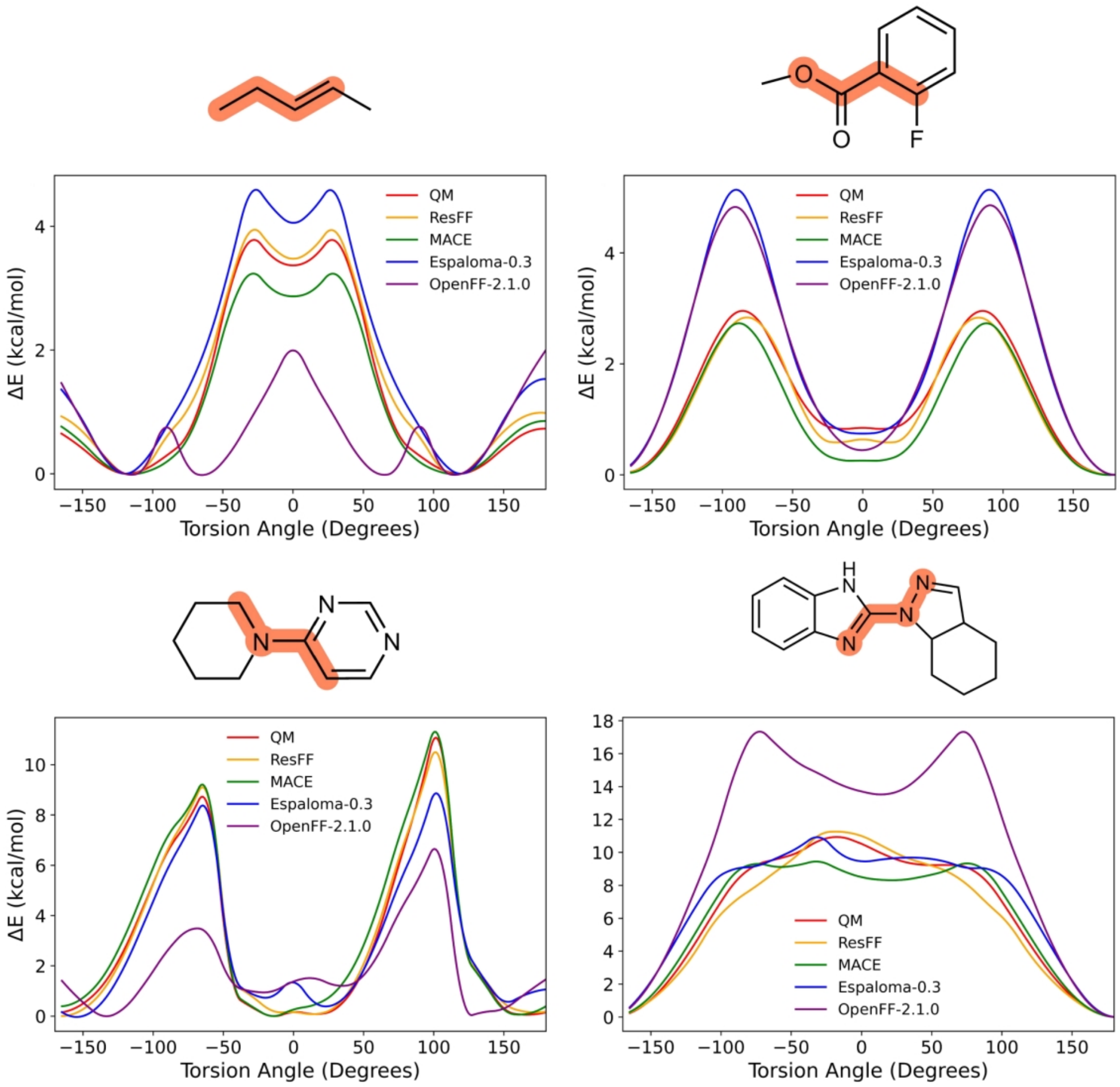

Supplementary Figure 1. Examples of torsion potential energy surface prediction of models on the TorsionNet-500 dataset, compared with the QM references. The torsion angle of each molecule is highlighted in red color. Source data are provided as a Source Data file.

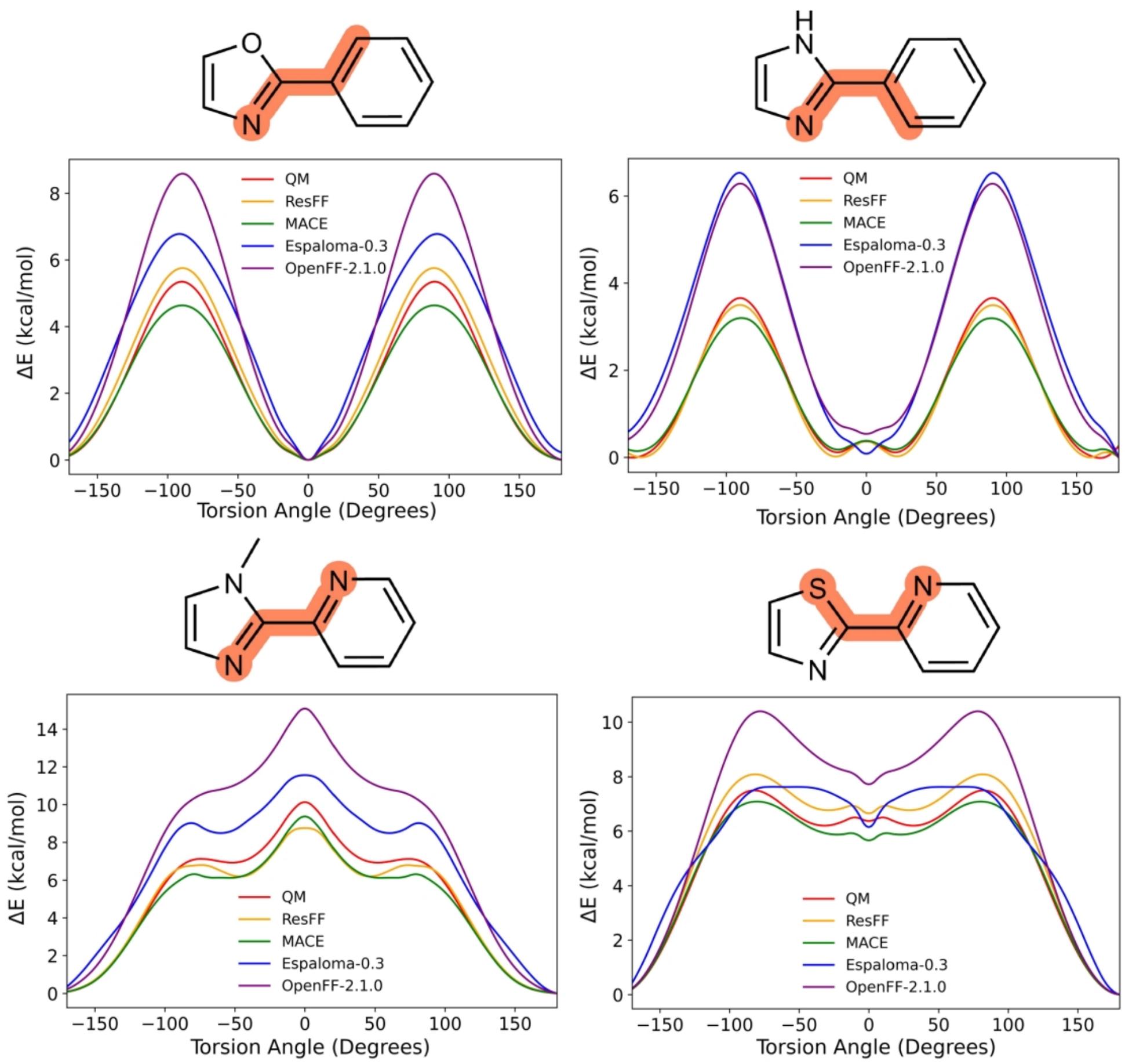

Supplementary Figure 2. Examples of torsion potential energy surface prediction of models on the Torsion Scan dataset, compared with the QM references. The torsion angle of each molecule is highlighted in red color. Source data are provided as a Source Data file.

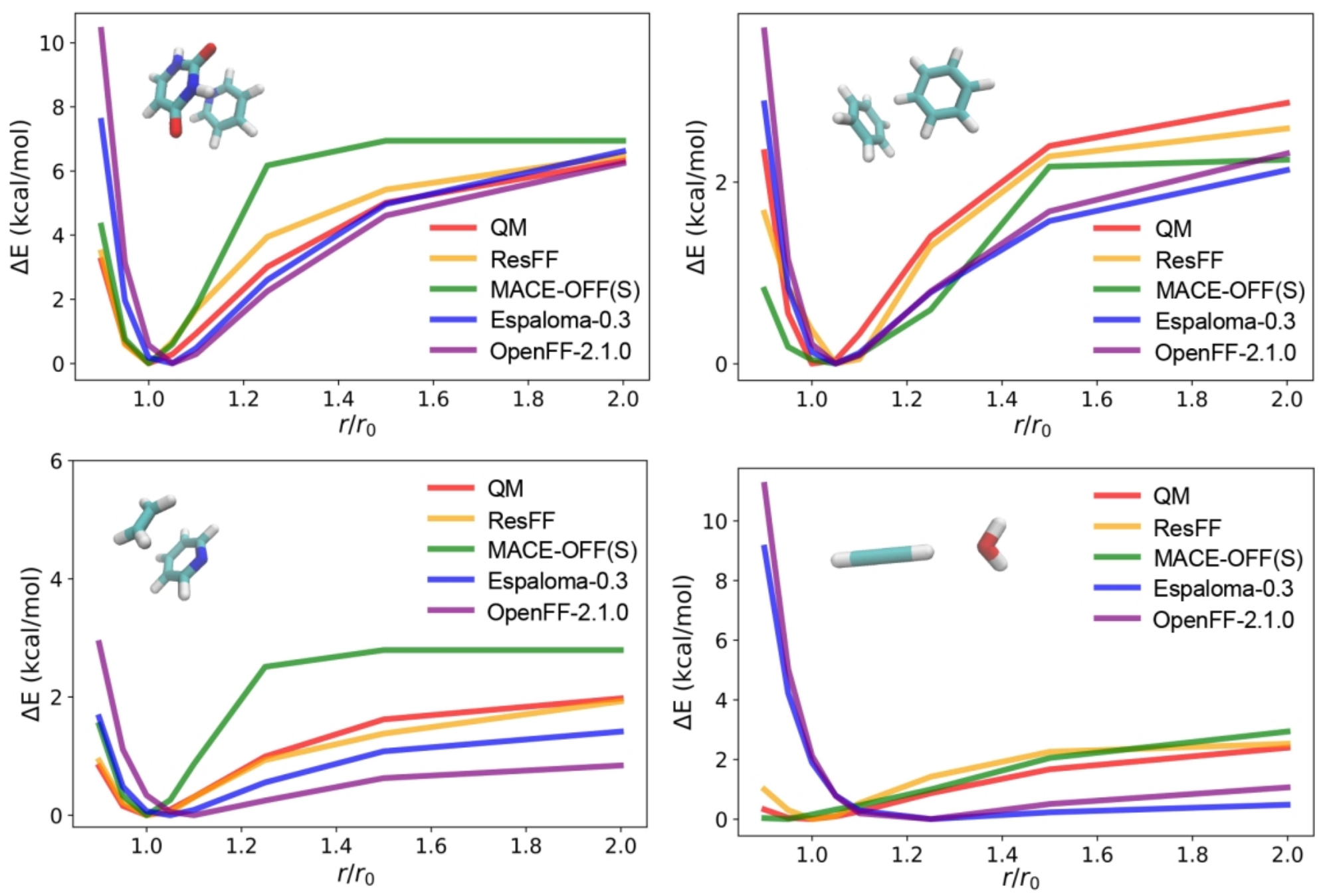

Supplementary Figure 3. Examples of dimer energy profiles of models on the S66×8 dataset, compared with the QM references. Source data are provided as a Source Data file.

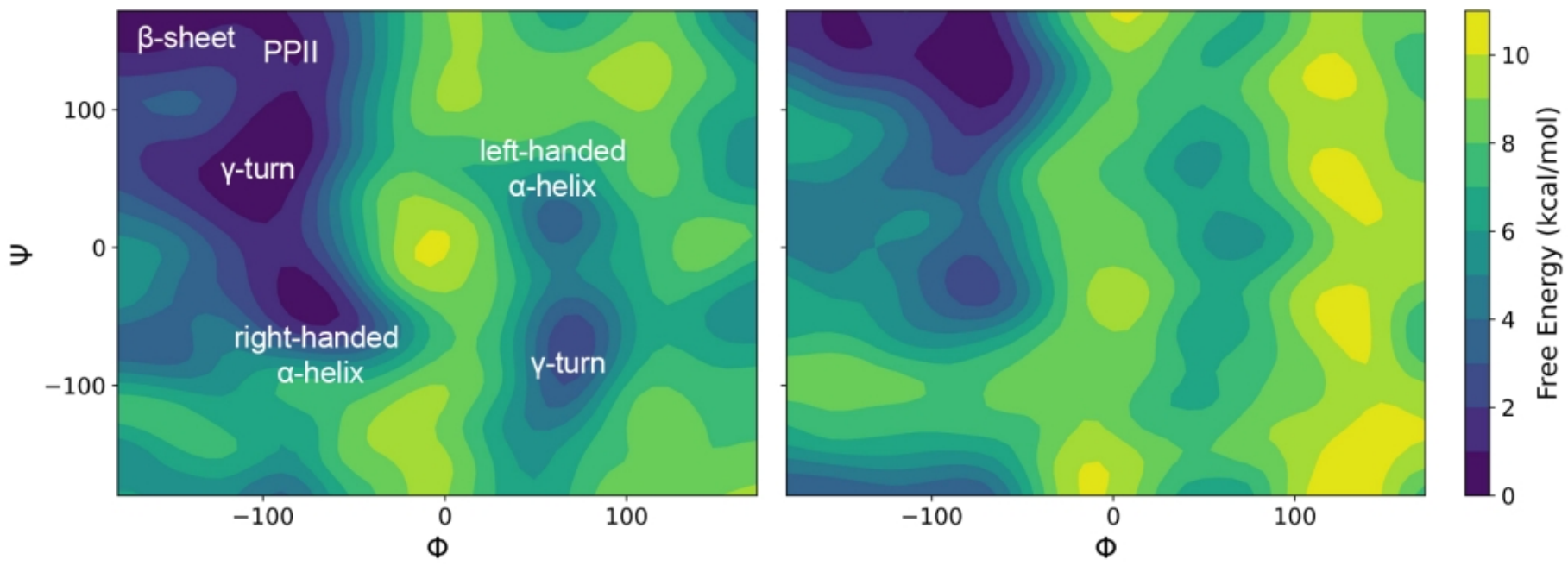

Supplementary Figure 4. Free energy surface of tetrapeptide. Free energy surface of the tetrapeptide generated by ResFF (left) and AMBER (right) in explicit solvent at 500 K for 1 ns simulation. Source data are provided as a Source Data file.

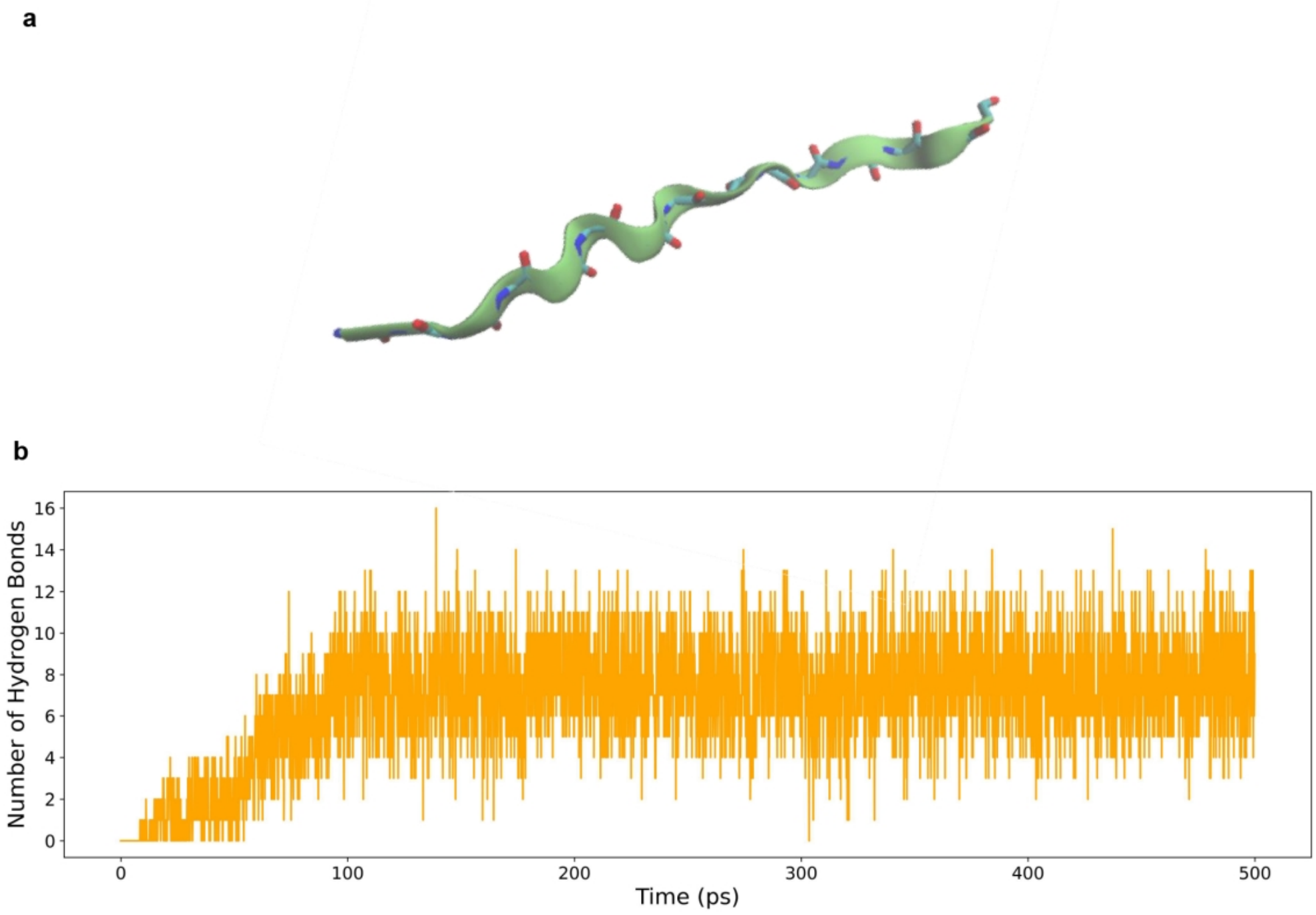

Supplementary Figure 5. Polyalanines folding dynamics. (a) Illustration of short-time wavy conformation that occurs in polyalanine folding. **(b)** Number of hydrogen bonds formed during the polyalanine folding process. Source data are provided as a Source Data file.

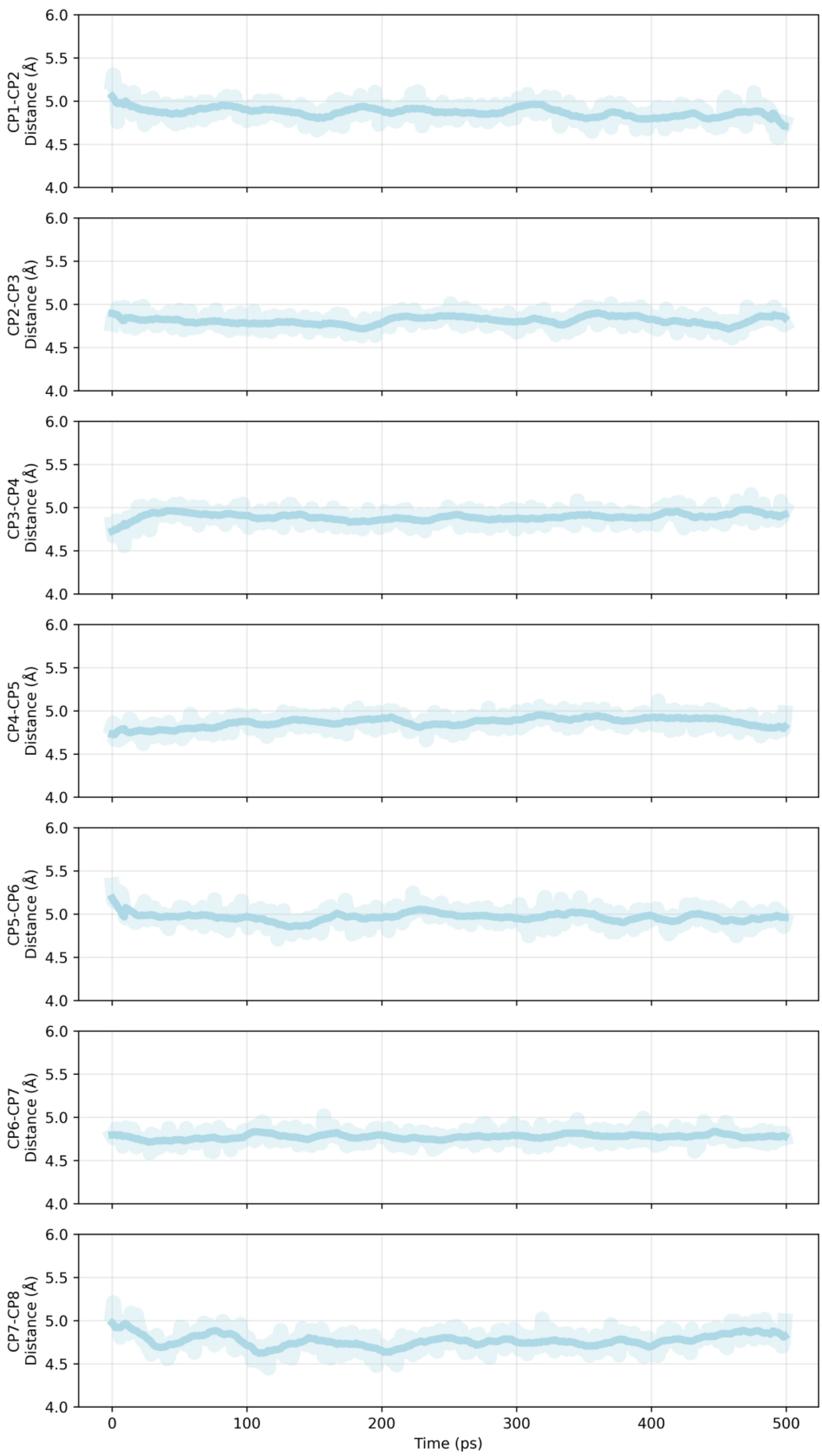

Supplementary Figure 6. **Inter-ring distance of each cyclic peptide (CY) in α-alt(D,L)-peptide assembly during the simulation.** The shaded area shows fluctuations in the inter-ring distance, while the solid line represents the trajectory smoothed using a 15-point moving average. Source data are provided as a Source Data file.

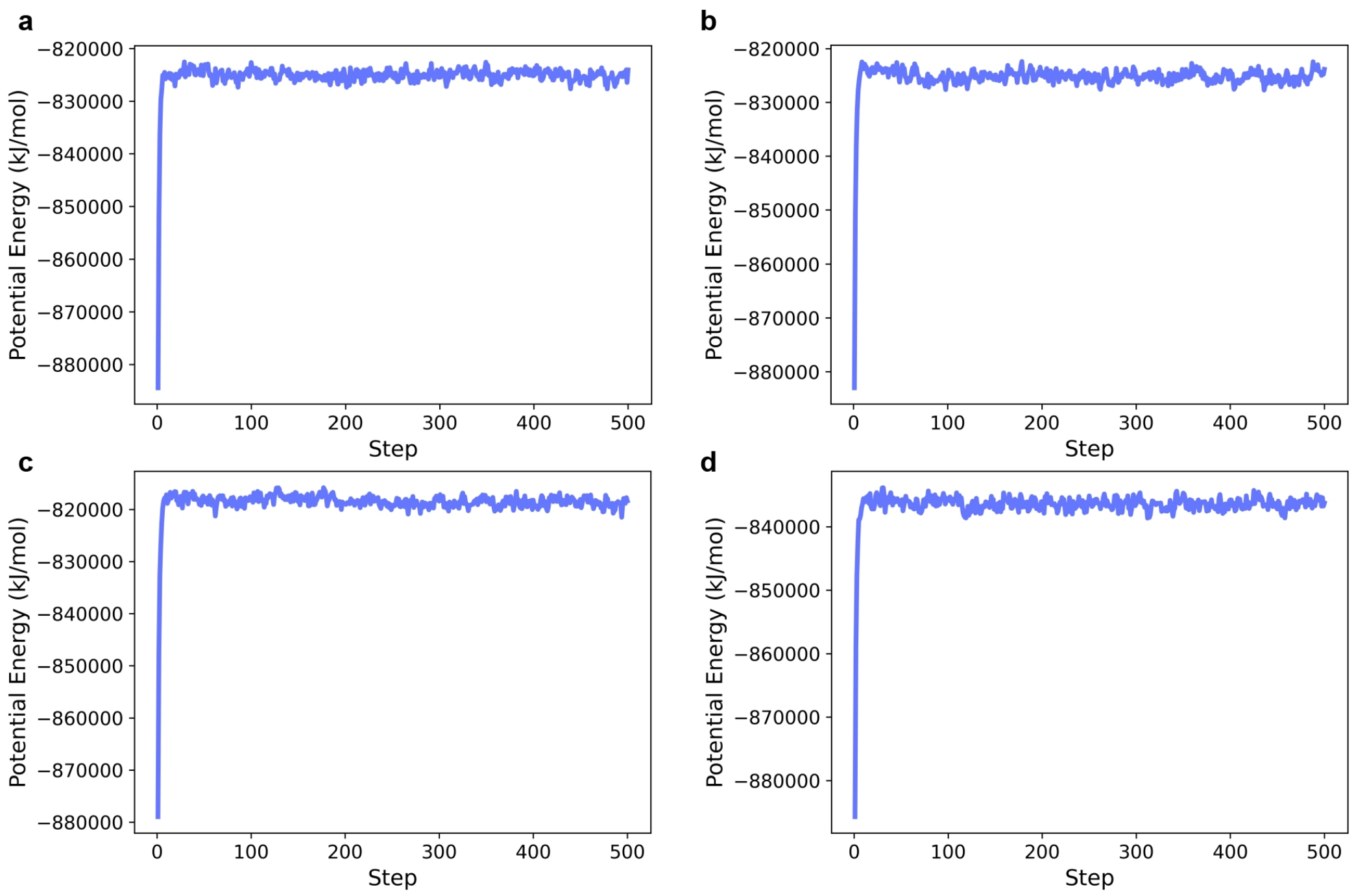

Supplementary Figure 7. Potential energy fluctuation of representative force fields for the ligand in Tyk2 complex systems dynamic simulation. (a) OpenFF-2.1.0. **(b)** Espaloma-0.3. **(c)** ResFF. **(d)** MACE-OFF(S). Source data are provided as a Source Data file.

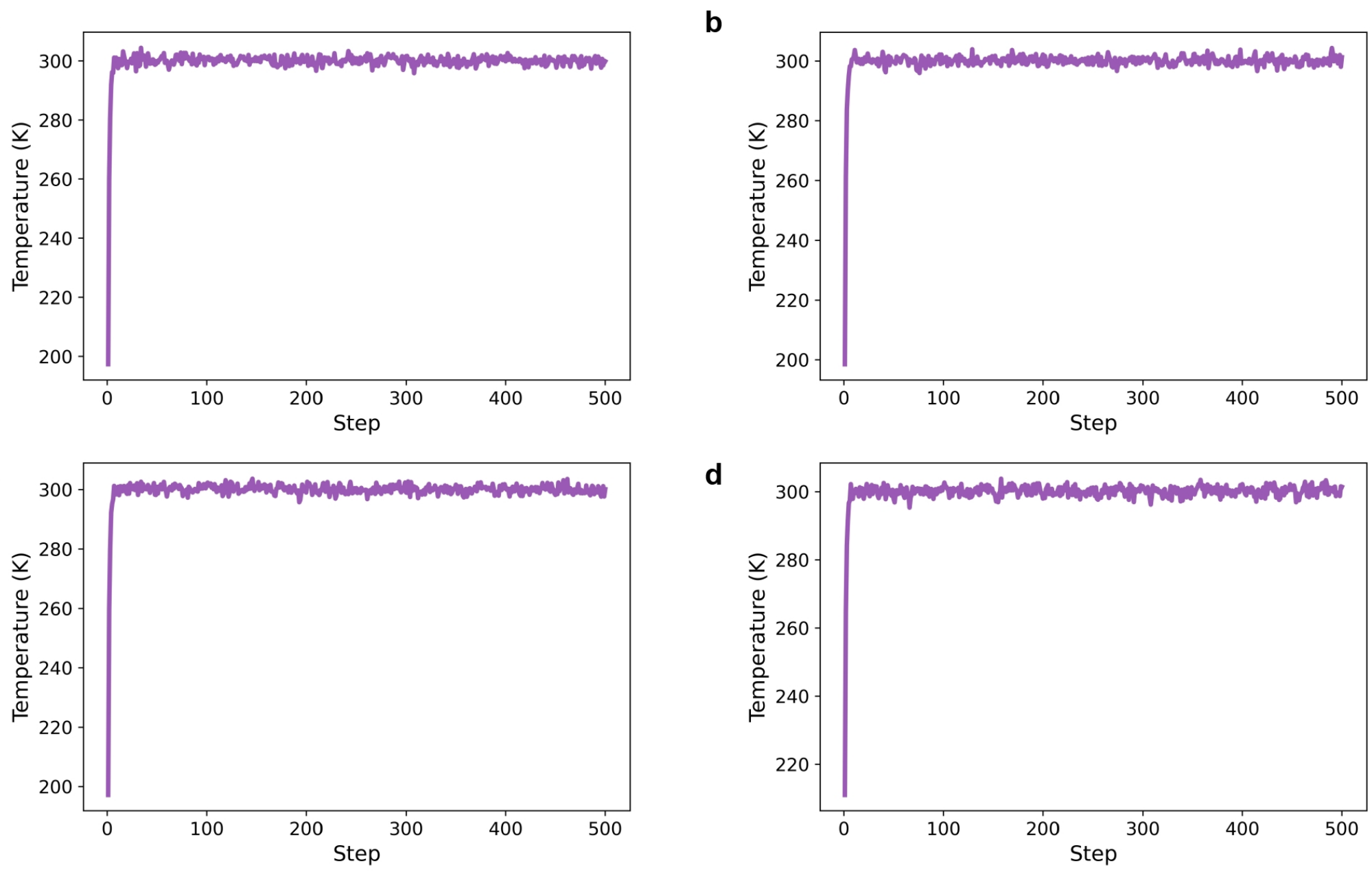

Supplementary Figure 8. Temperature fluctuation of representative force fields for the ligand in Tyk2 complex systems dynamic simulation. (a) OpenFF-2.1.0. **(b)** Espaloma-0.3. **(c)** ResFF. **(d)** MACE-OFF(S). Source data are provided as a Source Data file.

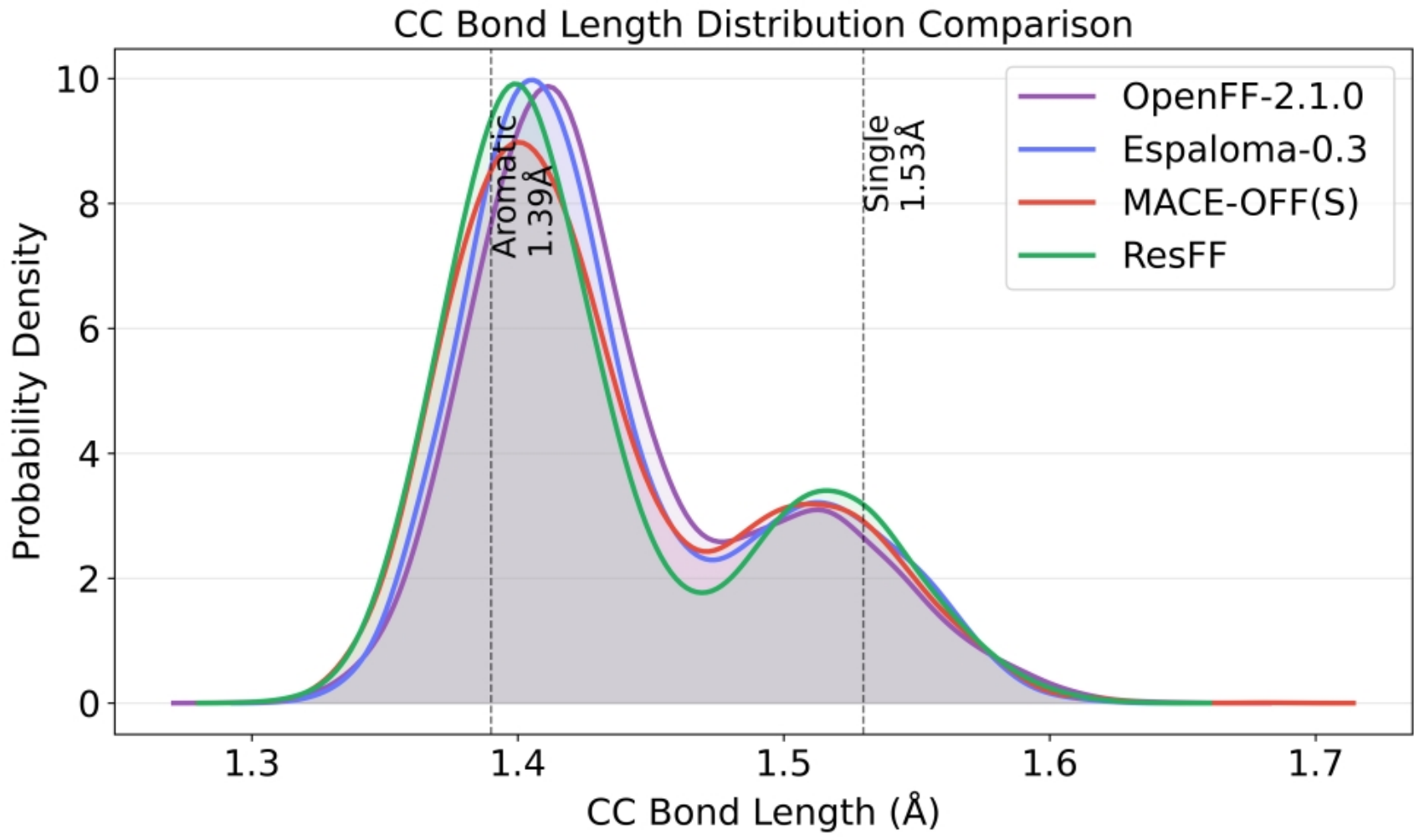

Supplementary Figure 9. Bond length analysis of representative force fields for the ligand in Tyk2 complex systems dynamic simulation. Reference values for standard bond lengths are shown (C-C single: 1.53 Å[1]; aromatic: 1.39 Å[2]). Source data are provided as a Source Data file.

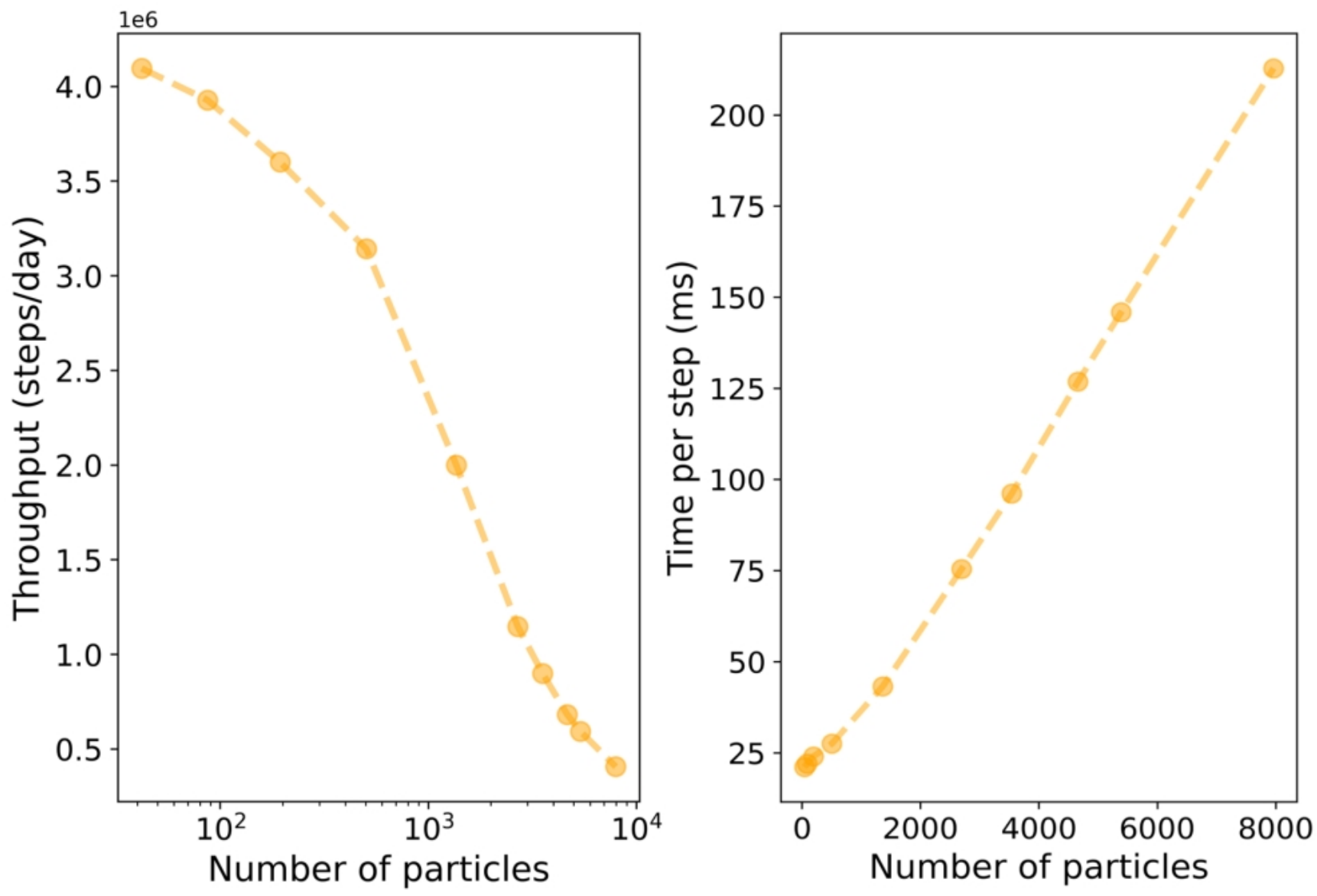

Supplementary Figure 10. Scaling analysis of ResFF. The plot illustrates simulation performance for molecular systems ranging from small peptides (42 atoms) to large proteins (7962 atoms). Benchmarks were conducted on a single NVIDIA A100 GPU using OpenMM version 8.1.2. Source data are provided as a Source Data file.

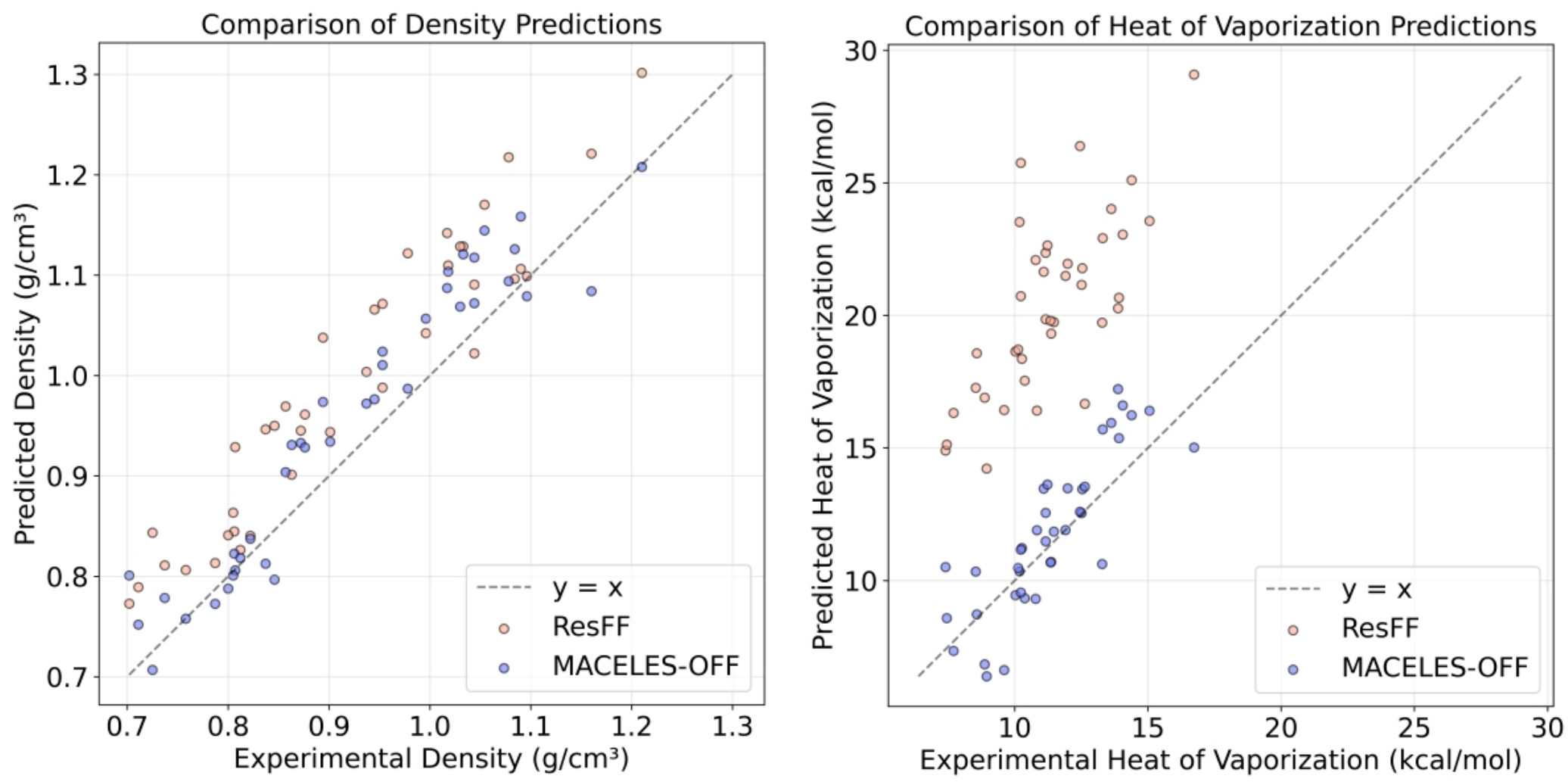

Supplementary Figure 11. Liquid property simulations of organic molecules. Predicted liquid densities (left) and enthalpies of vaporization ΔH_{vap} (right) for ResFF and MACELES-OFF. Source data are provided as a Source Data file.

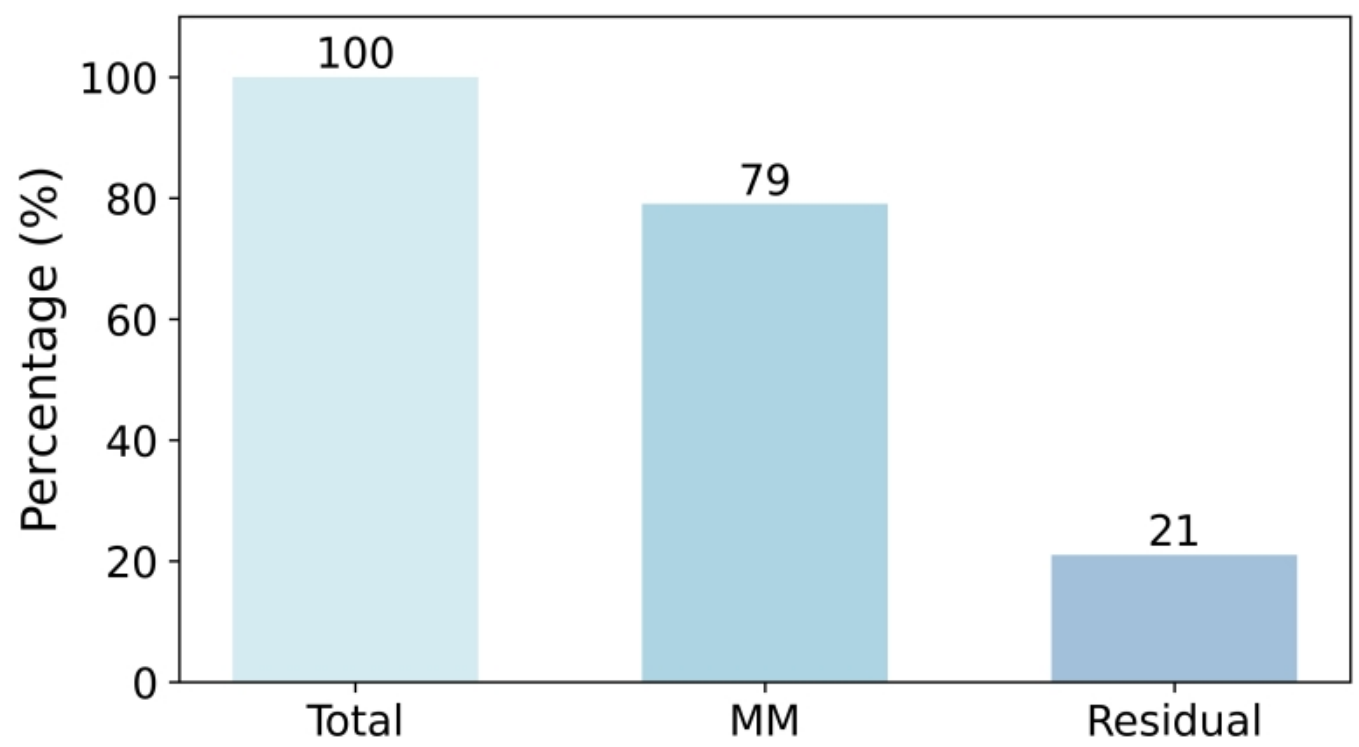

Supplementary Figure 12. Energy Decomposition of ResFF. Relative energy contributions are averaged over all molecules in the Torsion Scan dataset. The MM module accounts for the majority of the total energy, contributing about 79%, while the residual term represents only a small fraction of 21%. Since the MM module is first optimized to fit the QM reference with a certain level of accuracy, it leaves only minor energy deviations, which the residual module can then further refine more effectively.

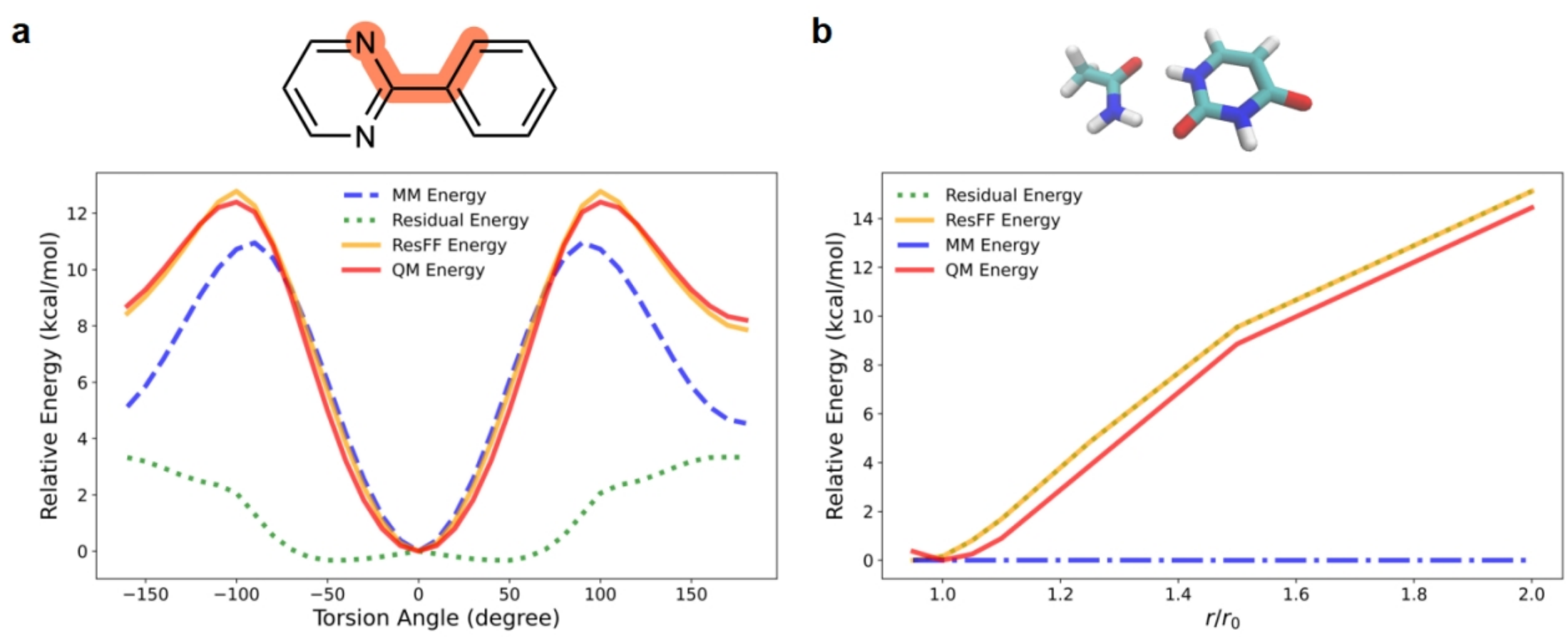

Supplementary Figure 13. Energy decomposition examples. (a) An energy decomposition example in the Torsion Scan dataset. The MM component describes the overall shape of the QM torsional energy profile, while the residual module provides corrections that improve agreement in regions

where the MM description is less accurate. **(b)** An energy decomposition example of $AcNH_2$-Uracil in the S66×8 dataset. For dimer distance scans, the internal geometries of the monomers are fixed and only the intermolecular distance varies. As a result, the relative energy of MM component remains zero across the scan, while the energy variation is entirely contributed by the residual term, capturing subtle intermolecular interactions that MM module is not designed to describe. Source data are provided as a Source Data file.

Supplementary Tables 1 to 4

Supplementary Table 1. ResFF performance on SPICE test set.

Molecule	Conformation	MAE (kcal/mol)	RMSE (kcal/mol)
Neutral	90435	1.36	2.96
Charged	7466	2.52	4.62

Supplementary Table 2. Performances of different methods on the TorsionNet-500 dataset and Torsion Scan dataset. Metrics include RMSE, MAE, Pearson correlation r, and coefficient of determination R^2**.** Best results are in bold, and second-best result are underlined.

Dataset	Methods	RMSE (kcal/mol)	MAE (kcal/mol)	Pearson's r	R^2
TorsionNet-500	MACE-OFF(S)	0.51	0.34	0.982	0.965
	MACE-OFF(M)	0.28	0.19	<u>0.995</u>	0.989
	MACE-OFF(L)	**0.21**	**0.14**	**0.997**	**0.994**
	MACELES-OFF	<u>0.22</u>	<u>0.15</u>	**0.997**	<u>0.993</u>
	ResFF	0.72	0.45	0.965	0.930
Torsion Scan	MACE-OFF(S)	0.43	0.28	0.988	0.976
	MACE-OFF(M)	0.22	0.16	<u>0.997</u>	0.994
	MACE-OFF(L)	<u>0.19</u>	<u>0.13</u>	**0.998**	<u>0.995</u>
	MACELES-OFF	**0.16**	**0.11**	**0.998**	**0.997**
	ResFF	0.90	0.50	0.953	0.896

Supplementary Table 3. Performances of different methods on the S66×8 dataset. Metrics include RMSE, MAE, Pearson correlation r, and coefficient of determination R^2**.** Best results are in bold, and second-best result are underlined.

Methods	RMSE (kcal/mol)	MAE (kcal/mol)	Pearson's r	R^2
MACE-OFF(S)	0.62	0.37	0.975	0.910
MACE-OFF(M)	<u>0.36</u>	<u>0.19</u>	<u>0.990</u>	<u>0.970</u>
MACE-OFF(L)	**0.31**	**0.16**	**0.993**	**0.977**
MACELES-OFF	0.41	0.21	0.985	0.953
ResFF	0.55	0.39	0.970	0.929

Supplementary Table 4. Ablation Experiments of ResFF. Ablation studies are conducted on the residual module and on the joint optimization of ResFF to assess their respective contributions. *ResFF (MM only)* refers to the model trained solely on the MM module, *ResFF (w/o joint optimization)* denotes the model trained with both modules but without joint optimization, and *ResFF* represents the full model trained with the three-stage strategy. All three models are trained on the SPICE dataset under identical hyperparameter settings and evaluated on the Torsion Scan dataset. *ResFF (w/o joint optimization)*, with the additional residual component, outperforms *ResFF (MM only)*, while joint optimization yields further improvements. Best results are in bold, and second-best results are underlined.

Models	w/ residual module	w/ joint optimization	RMSE (kcal/mol)	MAE (kcal/mol)	Pearson's *r*	R^2
ResFF (MM only)	×	×	1.22	0.86	0.917	0.807
ResFF (w/o joint optimization)	√	×	<u>0.96</u>	<u>0.63</u>	<u>0.950</u>	<u>0.880</u>
ResFF	√	√	**0.90**	**0.50**	**0.953**	**0.896**

Supplementary Note 1

Supplementary Note 1. Condensed Phase Benchmarks

As an exploratory transferability test, we performed an additional condensed-phase benchmark on 39 organic liquids taken from previous research[3]. Specifically, we evaluated liquid densities and enthalpies of vaporization (ΔH_{vap}) using ResFF and compared the results with the reported MACELES-OFF data.

ResFF simulations were performed in OpenMM following the condensed-phase protocol described in previous studies[4, 5]. Liquid boxes containing 64 molecules were initialized using packmol[6], with box vectors adjusted to 80% of the experimental density. Structures were first minimized using the L-BFGS algorithm. MD simulations were then performed in the NPT ensemble at 298.15 K using a Langevin integrator with a 1 fs timestep and a Monte Carlo barostat at 1 atm. Trajectories were propagated for 300 ps, and the final 100 ps were used for density and energy averaging. ΔH_{vap} was calculated from the difference between the average gas-phase molecular energy and the average per-molecule liquid-phase energy, with the thermal correction included according to the protocol in previous studies[4, 5]. Data for MACELES-OFF were taken from previous research[3].

As shown in Supplementary Fig. 11, ResFF achieves a high Pearson correlation coefficient of 0.95 for liquid density. This result suggests that ResFF can capture local packing environments and short- to medium-range intermolecular interactions reasonably well for simple organic liquids, despite not being fitted to condensed-phase observables. In contrast, the correlation for ΔH_{vap} is more moderate (Pearson $r = 0.72$), accompanied by a systematic deviation. This behavior is expected because ΔH_{vap} is an integrated thermodynamic quantity that is sensitive to cumulative long-range dispersion, electrostatic interactions, and condensed-phase polarization effects. These contributions are not explicitly included in the current ResFF architecture and fall outside its primary design scope.

For comparison, MACELES-OFF achieves Pearson correlations of 0.96 for liquid density and 0.84 for ΔH_{vap} . Its improved performance is consistent with its explicit treatment of long-range electrostatics and further highlights the importance of physically motivated long-range terms for quantitative condensed-phase thermodynamic predictions.

These results indicate that ResFF retains numerical stability and reasonable transferability for simple organic liquids, particularly for properties dominated by local packing interactions. However,

ResFF should not be regarded as a condensed-phase-optimized force field. Quantitative prediction of bulk thermodynamic properties, such as enthalpies of vaporization, dielectric responses, crystal lattice energies, or material properties, would require explicit long-range treatments and/or retraining on appropriate condensed-phase reference data.

Supplementary References

1. Lide, D. R. *CRC Handbook of Chemistry and Physics*. (CRC Press, 2005).
2. McClain, K. R. et al. Linear Inverse Sandwich Complexes of Tetraanionic Benzene Stabilized by Covalent δ-Bonding with Late Lanthanides. *J. Am. Chem. Soc.* **146**, 32708-32716 (2024).
3. Kim, D. et al. A universal augmentation framework for long-range electrostatics in machine learning interatomic potentials. *J. Chem. Theory Comput.* **21**, 12709-12724 (2025).
4. Batatia, I., Kov'acs, D. a. P. e., Simm, G. N. C., Ortner, C. & Csányi, G. MACE: Higher Order Equivariant Message Passing Neural Networks for Fast and Accurate Force Fields. Preprint at https://arxiv.org/abs/2206.07697 (2022).
5. Kovács, D. P. et al. MACE-OFF: Short-Range Transferable Machine Learning Force Fields for Organic Molecules. *J. Am. Chem. Soc.* **147**, 17598-17611 (2025).
6. Martínez, L., Andrade, R., Birgin, E. G. & Martínez, J. M. PACKMOL: A package for building initial configurations for molecular dynamics simulations. *J. Comput. Chem.* **30**, 2157-2164 (2009).
