# Supplementary material for: Deep residual learning for molecular force fields
Source: Nat Commun. 2026 Jun 30;17:8160. doi: 10.1038/s41467-026-74983-0 (PMC13458457; doi:10.1038/s41467-026-74983-0)
Supplement: Supplementary file 2 [file 41467_2026_74983_MOESM2_ESM.pdf]

nature portfolio

Peer Review File

Deep Residual Learning for Molecular Force Fields

Corresponding Author: Professor Mingyue Zheng

This file contains all reviewer reports in order by version, followed by all author rebuttals in order by version.

Version 0:

Reviewer comments:

Reviewer #1

(Remarks to the Author)
In the manuscript titled "Deep Residual Learning for Molecular Force Fields", Jiang and co-workers presented ResFF, a hybrid force field model that combines physics-based molecular mechanics terms with neural residual corrections. The module, built on a 2D graph neural network, predicts covalent parameters and analytically computes bond, angle, and torsion energies. The residual module, based on an equivariant neural network, learns remaining interactions and adds corrective energy terms. By summing outputs from both components, ResFF aims to retain the interpretability and transferability of classical force fields while leveraging neural networks to capture subtle noncovalent effects. The authors also acknowledge key limitations, such as the restricted accuracy of analytic covalent forms and the engineering challenges of integrating the hybrid model into molecular-dynamics packages.
Overall, I personally think the originality of this ResFF work is reasonably good. Its "Residual Learning" is essentially the same concept as δ-learning, and there are already many related papers using δ-learning in the AI4S and AI4M fields. However, this paper identifies a very good entry point and clearly explains how its approach differs from previous δ-learning methods. The manuscript is well written. Although the technical difficulty of the work is not high, the perspective, ideas, and writing are all commendable.

I have several comments and suggestions as follows:
1. ResFF is primarily trained on small, drug-like neutral molecules from SPICE and related datasets. The text emphasizes extrapolation to monomers and dimers, but the model does not evaluate on common charged species, macromolecules, metals, or reactive systems. Otherwise, the current applicability is limited to neutral organic molecules and discuss expected behavior for ions, transition-metal complexes, and biopolymers. It is better to explain to users when retraining or fine-tuning is required.
2. The SI shows that the MM module contributes ~79% and the residual term ~21% of total energy, assuming linear additivity. For strongly coupled systems, could nonlinear coupling terms be more appropriate? Moreover, it is better to visualize this decomposition.
3. In comparison with polarizable or many-body expansions, whether the residual term implicitly encodes higher-order many-body effects, and how double-counting between MM and residual components is avoided.
4. For materials or condensed-phase systems, can the local message-passing design of the residual module capture long-range dispersion and electronic-screening effects?
5. The MD demonstrations are persuasive but limited in scale. To establish broader applicability, I suggest the authors test ResFF on larger systems. Moreover, MACE instability is shown through a single bond collapse. I suggest to do a more systematic comparison of MD stability.

(Remarks on code availability)

Reviewer #2

(Remarks to the Author)
The authors present the ResFF force field for molecular modelling in which a MM force field (bonds, angles, torsions) is supplemented by residual corrections from a neural network. The model is trained on

the SPICE QM dataset to create a transferable model that is tested on various organic molecule benchmarks.

The model is clearly described and shows promising performance, but benchmarks against state-of-the-art machine learning potentials, both in the gas and condensed phase, as well as scaling tests to profile the computational expense are needed to better understand the advantages and disadvantages of the residual corrections, before publication.

Major comments:

- The residual part of the FF seems to be used to model long-ranged electrostatics and dispersion, as well as correcting the valence terms. This is an interesting choice, as actually we know what the physics-based equations are for electrostatics and dispersion. Many machine learning potentials (MLPs) are going the other way and adding explicit electrostatics to local models (eg https://arxiv.org/abs/2507.14302). A comment on these contrasting approaches would be useful.

- With the above in mind, we need to see testing of ResFF for condensed phase modeling. Eg MACE-OFF is tested on densities and enthalpies of vaporization of liquids, as well as crystal data. This is a much more stringent test of the developed nonbonded interactions than what is presented here. As presented, it is unclear whether ResFF is intended for condensed phase modeling.

- It should be noted that the MM FFs (eg OpenFF and Espaloma) are not expected to do well on the DES370K as they are effective fixed charge force fields for condensed phase modeling. Eg if we look at the water-water dimer in Fig 4, OpenFF does very badly, yet models the condensed phase properties of water very well. For the application area of these force fields, condensed phase accuracy is more important than dimer interactions.

- Section 2.2. It should be noted in the main text that the experiment here is done with the small MACE model, trained only on energies (not forces), which is not the ideal method to train a MLP. Further, as I understand it, DES370K is only testing the residual part of the FF, since the bonded interactions are fixed? The higher accuracy of ResFF could just be the result of a longer-range receptive field and we can clearly see that in Fig S3 where interactions beyond the MACE cutoff are inaccurate. Why not compare with the MACE-OFF23(L) model, with a longer-range receptive field, or even better a model such as MACELES-OFF that includes long-ranged electrostatics?

- I have similar comments throughout the manuscript. The claim at the end is that ResFF out-performs the state-of-the-art. But MACE-OFF23(S) is not even the SOTA in the MACE-OFF paper. The authors should compare throughout with the large model and/or a non-local model that treats long-ranged interactions, otherwise it is not clear to me what the application area of ResFF is?

- Table 2. Here the claim is that the small MACE model outperforms ResFF, but ResFF is more useful because MACE underestimates barrier heights. If MACE does better overall, but worse near the barrier, then ResFF must do worse in low energy regions? The low energy regions are the ones that are sampled in MD, so it seems more important to get these right?

- Fig 5. The claim here is that MACE is less accurate than OpenFF for modelling energetics of the industry benchmark set? This is a very expected result that needs some diagnosis. One aspect could be that MACE is not trained on charged molecules. I suggest to investigate what happens when a model that is trained to represent these cases (such as MACE-omol (https://github.com/ACEsuit/mace/releases) or MACELES-OFF) is used. Otherwise the authors are comparing with a model taken out of its area of applicability, and cannot make useful

statements about ResFF accuracy relative to SOTA.

- Biological testing. Why do the authors not use ResFF for the entire system? Is the MACE timing for the tetrapeptide (2.2x10^6 steps/day) measured by the authors or taken from the MACE-OFF paper? If the latter, this seems to be the time for the tetrapeptide plus water box (with everything modeled using MACE-OFF24(M), not the small model that the authors have been comparing to). How does the computation time for ResFF scale with number of atoms in the system?

- Fig 6a. The gamma-turn region observed here seems much deeper than usually oberserved. How does this compare to eg the free energy surface from the AMBER force field?

Minor comments:

- Note that precision does not mean the same as accuracy. Throughout, the authors tend to use precision when I think they mean accuracy.

- In Table 1, why are some R^2 values missing?

- For polyalanine, please explain what is meant by wavy intermediate here? What feature of the data indicates it is present?

- eq 10. The units of energy and force losses are important here when setting lambda to 1.

- eq 12. I guess there should not be a lambda on the LHS?

(Remarks on code availability)
I was able to install and run the code. I could not see any information on how to reproduce the tables/figures in the paper. For example, I could not see a straightforward tutorial demonstrating how to assign force field parameters to a single molecule and e.g run molecular dynamics, nor would I know where to start to reproduce eg Fig 3c.

Version 1:

Reviewer comments:

Reviewer #1

(Remarks to the Author)
I appreciate that the authors have made significant improvements in the revision. The new test on the charged molecules and energy decomposition provides a clearer figure to the readers. The test on a large molecular system also demonstrates the good performance of the ResFF model. Although the limitation on the metals, reactive systems and macromolecules in my first comment has not been solved, the authors have narrowed the applicability domain in the revised manuscript. Regarding my Comment 4, the authors also claimed the limitation of current ResFF on the long-range electrostatic and dispersion in the revision, and stated its application domain in drug-like molecular systems where local interactions dominate. I think these clarifications of limitations and application domain of ResFF are good enough although some of my comments have not been addressed. Overall, I recommend this version for publication in NC.

(Remarks on code availability)
The code information is clear with detailed comments, which is usable resource for the community.

Reviewer #2

(Remarks to the Author)
The authors have, for the most part, responded sufficiently to the comments raised. As such, the strengths and limitations of the ResFF approach are much clearer.

I recommend publication subject to consideration of the following points:

- I am still uncomfortable with the R^2 data in Table 1, particularly the negative values, and I suggest the authors re-check these. I understand this can happen, but not why these models should look so bad. Do the data need to be shifted so that the means align?

- The authors have mis-interpreted the statement on speed in the MACE-OFF paper. "Figure 8 shows the free energy surface of Ala3 in water, modeled by AMBER14SB/TIP3P and MACE-OFF24(M)". They mean that the top panel in Fig 8 is modeled by AMBER14SB/TIP3P (ie classical) and the bottom panel with MACE-OFF24(M) (ie MLP). You can also see in Figure 12, a throughput of around 3M steps/day for ~1K atoms. I do not think it is worth discussing at length. The authors can just remove this statement, and mention that the two methods are similar in throughput.

- p32. The authors have provided condensed phase benchmarks in their response, but I do not see them in the revised paper. I suggest to include, at least in SI, as it will stop others from trying to apply the methods outside their intended application area.

- p34. Please also mention how improper torsions are handled.

- p39. The figure of 73301 drug-like moleucles does not appear to match the data in Table 4?

(Remarks on code availability)
I have not re-run the code after revision, but I thank the authors for updating the repository with the requested changes.

Open Access This Peer Review File is licensed under a Creative Commons Attribution 4.0 International License, which permits use, sharing, adaptation, distribution and reproduction in any medium or format, as long as you give appropriate credit to the original author(s) and the source, provide a link to the Creative Commons license, and indicate if changes were made.
In cases where reviewers are anonymous, credit should be given to 'Anonymous Referee' and the source.
The images or other third party material in this Peer Review File are included in the article's Creative Commons license, unless indicated otherwise in a credit line to the material. If material is not included in the article's Creative Commons license and your intended use is not permitted by statutory regulation or exceeds the permitted use, you will need to obtain permission directly from the copyright holder.
To view a copy of this license, visit https://creativecommons.org/licenses/by/4.0/

Reviewer #1 (Remarks to the Author):

In the manuscript titled "Deep Residual Learning for Molecular Force Fields", Jiang and co-workers presented ResFF, a hybrid force field model that combines physics-based molecular mechanics terms with neural residual corrections. The module, built on a 2D graph neural network, predicts covalent parameters and analytically computes bond, angle, and torsion energies. The residual module, based on an equivariant neural network, learns remaining interactions and adds corrective energy terms. By summing outputs from both components, ResFF aims to retain the interpretability and transferability of classical force fields while leveraging neural networks to capture subtle noncovalent effects. The authors also acknowledge key limitations, such as the restricted accuracy of analytic covalent forms and the engineering challenges of integrating the hybrid model into molecular-dynamics packages.

Overall, I personally think the originality of this ResFF work is reasonably good. Its "Residual Learning" is essentially the same concept as δ-learning, and there are already many related papers using δ-learning in the AI4S and AI4M fields. However, this paper identifies a very good entry point and clearly explains how its approach differs from previous δ-learning methods. The manuscript is well written. Although the technical difficulty of the work is not high, the perspective, ideas, and writing are all commendable.

Response： Thank you for your valuable time and for your positive feedback on the strengths of our work. Your suggestions have been instrumental in enhancing the overall quality and rigor of our paper. We have carefully addressed all specific comments below and revised the manuscript accordingly to further clarify the positioning, scope, and practical implications of ResFF. We hope that these clarifications and the corresponding revisions have adequately addressed your concerns.

Comments 1:

ResFF is primarily trained on small, drug-like neutral molecules from SPICE and related datasets. The text emphasizes extrapolation to monomers and dimers, but the model does not evaluate on common charged species, macromolecules, metals, or reactive systems. Otherwise, the current applicability is limited to neutral organic molecules and discuss expected behavior for ions, transition-metal complexes, and biopolymers. It is better to explain to users when retraining or fine-tuning is required.

Response: We thank you for raising this important point regarding the domain of applicability of ResFF. Clarifying the intended chemical space is essential for responsible and reproducible use of any force-field-like model.

In the present work, ResFF is trained and validated primarily on small, drug-like organic molecules represented by the SPICE datasets (excluding metal-containing systems). Accordingly, the goal of this study is to evaluate performance and generalization within this chemical space rather than to claim universal transferability across all molecular classes. We have revised the manuscript (Discussion, line 581-586) to explicitly state this applicability domain.

Although the SPICE dataset includes both neutral and charged organic molecules, the charged species constitute a smaller fraction and primarily correspond to isolated gas-phase ions sampled in quantum-chemical calculations. These configurations probe local electronic structure but do not represent long-range Coulomb interactions characteristic of condensed-phase ionic environments. Consistent with this, ResFF does not employ explicit atomic charges or a long-range Coulomb term; instead, electrostatic effects are represented implicitly through the interactions learned from the training data. As a result, the model is expected to describe local interactions reliably but may not quantitatively reproduce long-range ion-ion interactions in bulk ionic or highly polarized environments. To clarify this behavior, we added a new analysis (Table S1) showing that the MAE on neutral molecules in the SPICE test set is 1.36 kcal/mol, whereas the MAE increases to 2.52 kcal/mol for charged species. This difference reflects the reduced representation of charged systems in the training data and indicates that such systems are only partially covered by the current model.

The current SPICE training data do not include macromolecules, transition-metal complexes, or reactive bond-forming/breaking processes. Therefore, ResFF is presently best suited for neutral, drug-like organic molecules and closely related fragments. In practical terms, retraining or fine-tuning should be considered when (i) new elements or coordination environments appear (e.g., transition metals or metal–ligand coordination), (ii) long-range electrostatics dominate the physics (e.g., salt bridges in highly charged biomolecules or concentrated ionic solutions), or (iii) chemical reactions or bond rearrangements are involved. The hybrid design of ResFF was intentionally chosen to facilitate future extensions beyond this initial domain. The analytical MM backbone provides a physically interpretable baseline, while the residual neural module is responsible for learning systematic corrections. To support

transparent use and extension, we provide pretrained model weights, example workflows, and documented fine-tuning procedures in our public GitHub repository (https://github.com/Ameki0/ResFF), enabling users to reproduce the reported results and adapt the model when their systems fall outside the defined applicability domain.

Table S1. ResFF performance on SPICE test set.

Molecule	Conformation	MAE (kcal/mol)	RMSE (kcal/mol)
Neutral	90435	1.36	2.96
Charged	7466	2.52	4.62

Comments 2:

(1) The SI shows that the MM module contributes ~79% and the residual term ~21% of total energy, assuming linear additivity. For strongly coupled systems, could nonlinear coupling terms be more appropriate? (2) Moreover, it is better to visualize this decomposition.

Response: We sincerely thank you for this insightful comment regarding the energy decomposition and the assumption of linear additivity.

(1) On linear additivity and possible nonlinear coupling

In ResFF, the total energy is expressed as

$$E_{\text{Total}} = E_{\text{MM}} + E_{\text{residual}} \quad ,$$

which represents a functional decomposition rather than a physical assertion that molecular energetics are intrinsically linear. The MM component provides a physically motivated baseline energy defined by an explicit analytical form, while the residual term is trained to reproduce the systematic difference between QM and MM energies.

Importantly, this formulation does not assume that nonlinear couplings are absent. Instead, the residual model learns the many-body and coupling effects missing from the classical functional form. Because the residual network depends on the full molecular geometry, it can represent interactions equivalent to bond-angle coupling, torsion-nonbonded coupling, and other higher-order effects that are not explicitly present in conventional MM force fields. In this sense, nonlinear physical effects are

captured within the residual energy rather than through additional analytical coupling terms.

The reported ~79% (MM) and ~21% (residual) contributions should therefore not be interpreted as a physically unique energy partitioning. The decomposition is model-dependent and serves only as an indicator of how much of the QM energy variation is already explained by the baseline MM functional form before learning corrections. For the systems considered in this work, primarily small, neutral, drug-like organic molecules, this separation has proven sufficient. In this regime, linear additivity provides an effective balance between accuracy, interpretability, and training stability, as demonstrated by the benchmark results and molecular dynamics tests presented in the manuscript. The observation that the majority of variation is captured by the MM term suggests that the residual model acts primarily as a structured correction rather than replacing the underlying force field.

For systems in which strong electrostatic coupling, coordination effects, or bond rearrangements dominate the energetics, such as highly ionic environments, transition-metal complexes, or reactive processes, more explicit coupling or polarization treatments may be required. In the line 555-558 of the revised Discussion, we have clarified this applicability boundary and explicitly noted that, although the total energy is written in additive form, nonlinear corrections are captured within the interaction radius of the residual model.

(2) Visualization of the decomposition.

Following your suggestion, we added new visualizations (Fig. S12) illustrating the decomposition across representative datasets.

For torsional scans, the MM component captures the dominant bonded and short-range energetic trends, while the residual term provides localized corrections in regions where classical force fields typically deviate from QM. For intermolecular interaction scans (e.g., S66×8), the intramolecular MM contribution remains essentially unchanged along the separation coordinate, and the distance-dependent interaction energy is largely described by the residual term. An example for the $AcNH_2$-Uracil dimer is now provided.

These analyses demonstrate that the relative roles of the MM and residual components are system-dependent: the residual model functions as a small correction in conformational energetics but can represent the dominant contribution for intermolecular interactions when required.

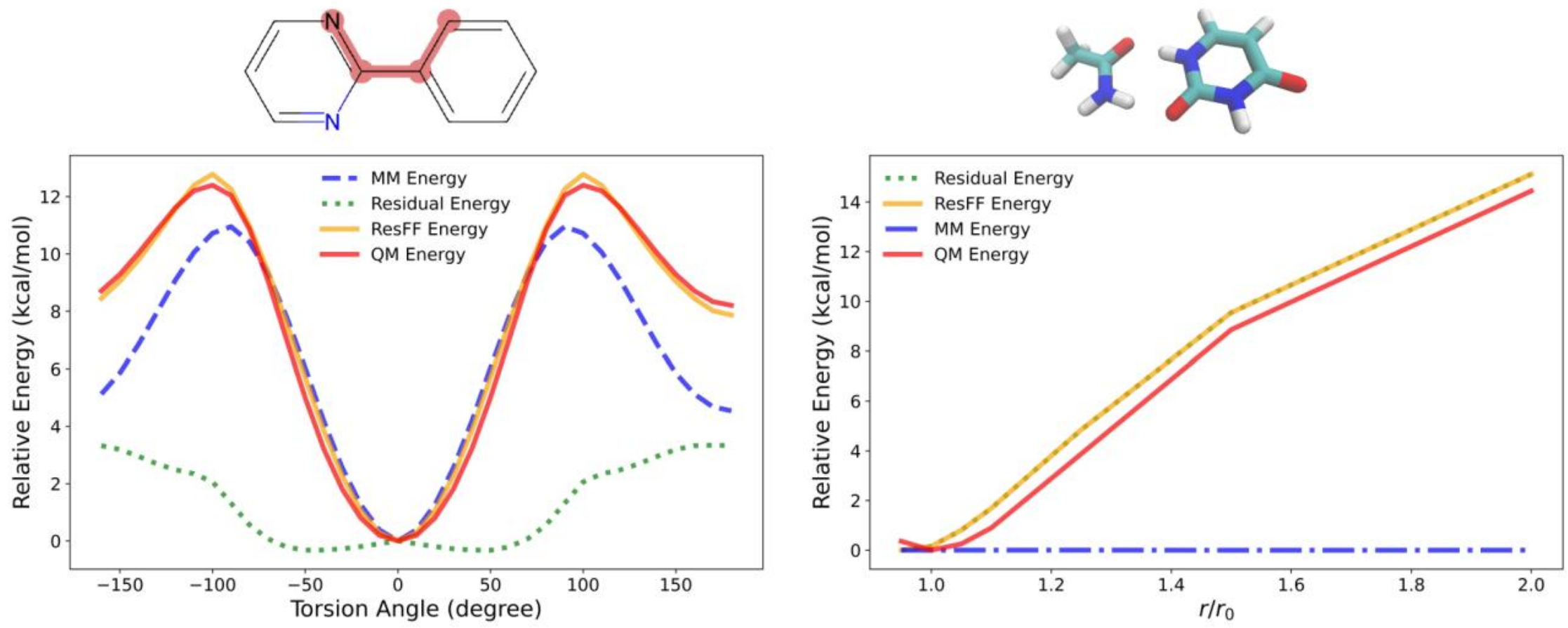

Figure S12. Energy decomposition examples. (a) An energy decomposition example in the Torsion Scan dataset. The MM component describes the overall shape of the QM torsional energy profile, while the residual module provides corrections that improve agreement in regions where the MM description is less accurate. **(b)** An energy decomposition example of $AcNH_2$-Uracil in the S66×8 dataset. For dimer distance scans, the internal geometries of the monomers are fixed and only the intermolecular distance varies. As a result, the relative energy of MM component remains zero across the scan, while the energy variation is entirely contributed by the residual term, capturing subtle intermolecular interactions that MM module is not designed to describe.

Comments 3:

(1) In comparison with polarizable or many-body expansions, whether the residual term implicitly encodes higher-order many-body effect. (2) How double-counting between MM and residual components is avoided.

Response: Thank you for this professional question regarding higher-order many-body effects and the possibility of double counting between the MM and residual components.

(1) Relation to many-body effects

The residual term in ResFF can be understood as implicitly capturing effective many-body and environment-dependent contributions, insofar as these effects are present in the QM reference data. It is important to emphasize that ResFF is not intended to provide an explicit or uniquely decomposable many-body expansion, as in polarizable force fields or formal many-body approaches (e.g., AMOEBA). Instead, the residual module serves as a data-driven correction that captures systematic deficiencies of the MM description in an effective manner.

(2) Avoidance of double counting

Potential double counting is avoided by defining the residual model as a correction relative to a fixed MM reference. During training, the residual network is fitted to the energy difference so any interaction already described by the MM baseline produces no learning signal. This constrains the residual model to learn only systematic deficiencies of the MM functional form rather than reparametrizing the entire potential energy surface.

Importantly, the MM parameters remain fixed during residual training, which fixes the reference energy gauge and prevents the neural model from arbitrarily redistributing energy contributions. A limited final fine-tuning stage is applied only to improve numerical consistency and stability; it does not modify the functional form of the MM terms nor alter the correction-based definition of the residual.

Taken together, the MM component provides a physically interpretable baseline potential, while the residual network acts as a structured correction anchored to that baseline. ResFF should therefore be viewed as a hybrid correction framework rather than a replacement for explicit polarizable or many-body force fields. This design represents a deliberate balance between physical interpretability, accuracy, and computational efficiency for drug-like molecular systems.

Comments 4:

For materials or condensed-phase systems, can the local message-passing design of the residual module capture long-range dispersion and electronic-screening effects?

Response: Thank you for this important question regarding the ability of the local message-passing residual module to describe long-range interactions in condensed-phase and materials systems.

The residual component of ResFF is constructed using a local message-passing architecture with a finite spatial cutoff. Consequently, it does not explicitly represent long-range physical effects such as asymptotic dispersion interactions or collective electronic screening. These interactions are known to be important for cohesive thermodynamic properties of bulk systems and are not rigorously captured by a purely local potential.

To assess the practical implications, we performed additional simulations for 39 organic liquids (Ref. [1]) and evaluated liquid density and enthalpy of vaporization ΔH_{vap}. As shown in Fig. R1, ResFF reproduces liquid densities with a high Pearson

correlation of 0.95. This behavior is consistent with statistical mechanical expectations, as equilibrium liquid density is primarily governed by short-range repulsion and local packing structure, which are well described by the present model. In contrast, ΔH_{vap} shows a moderate correlation of 0.72 with a systematic deviation. This is also physically expected: ΔH_{vap} depends on the cohesive energy of the liquid, which involves an integral over intermolecular interactions and is therefore highly sensitive to the long-range tail of dispersion and electrostatic interactions. Because these contributions extend beyond the cutoff of the local residual model, errors accumulate in bulk thermodynamic quantities.

These results indicate that ResFF provides an accurate short-range potential energy surface but does not aim to replace condensed-phase or materials force fields that explicitly treat long-range electrostatics and dispersion. Instead, its intended application domain is molecular conformational energetics and simulations of drug-like molecular systems where local interactions dominate. For applications requiring quantitatively accurate bulk thermodynamics, incorporation of explicit long-range treatments (e.g., Ewald electrostatics or analytical dispersion corrections) would be necessary. We have clarified this scope and added corresponding discussion in the revised Discussion (line 581-588).

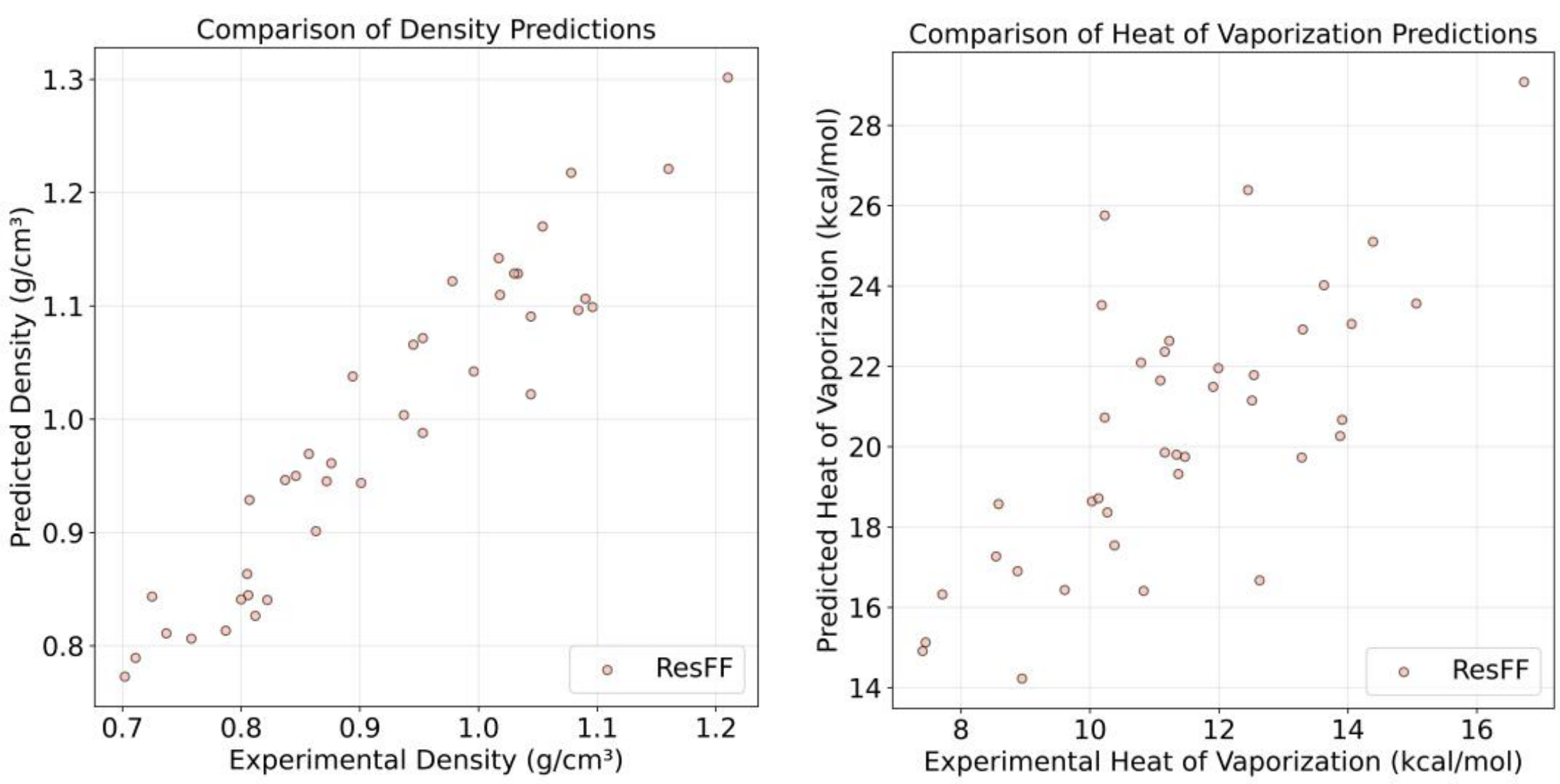

Fig. R1. Liquid property simulations of organic molecules. (left) Predicted liquid densities and (right) predicted enthalpies of vaporization ΔH_{vap} for ResFF. ResFF simulations were implemented in OpenMM following the condensed-phase protocol described in Ref. [2,3]. Liquid boxes containing 64 molecules were initialized using

packmol[4], with box vectors adjusted to 80% of the experimental density. Prior to MD production, structures underwent energy minimization via the L-BFGS algorithm. Simulations were conducted in the NPT ensemble at 298.15 K using a Langevin integrator with 1 fs timestep and a Monte Carlo barostat with 1 atm. Trajectories were propagated for 300 ps, with the final 100 ps used for density and energy averaging. ΔH_{vap} was calculated by comparing condensed-phase energies with vacuum-phase simulations of a single non-periodic molecule, using the same averaging window.

Comments 5:

(1) The MD demonstrations are persuasive but limited in scale. To establish broader applicability, I suggest the authors test ResFF on larger systems. (2) Moreover, MACE instability is shown through a single bond collapse. I suggest to do a more systematic comparison of MD stability.

Response: We sincerely thank you for the constructive suggestions regarding both the system size of the MD demonstrations and the need for a more systematic assessment of stability. We agree that stability, rather than a single illustrative trajectory, is critical for evaluating the practical usability of a new force field, and we have expanded our MD analyses accordingly.

(1) Larger systems

To examine behavior beyond the molecular sizes represented in the training data, we performed additional simulations on a substantially larger system: a cyclic peptide tubular assembly composed of eight stacked rings (848 atoms). Such assemblies are stabilized by cooperative hydrogen-bond networks and are sensitive to inaccuracies in intermolecular interactions. We carried out a 500 ps explicit-solvent simulation at 300 K using ResFF for both intra- and inter-peptide interactions.

Throughout the trajectory, the assembly remained structurally intact, maintaining persistent backbone hydrogen bonding. The average inter-ring distance (~4.85 Å) agrees well with the reported experimental value (~4.73 Å). Importantly, no integration instabilities, atom overlaps, or abnormal force spikes were observed during the simulation. The RMSD and distance distributions (Fig. 6d and Fig. S6) show bounded fluctuations consistent with a thermally equilibrated system. We have revised line 500-523 of Section 2.6 of the manuscript to include these new results.

(2) Systematic MD stability comparison

To move beyond a single collapse example, we performed protein-ligand MD simulations for the Tyk2 complex (PDB: 4GIH), using identical protocols while varying only the ligand force field (ResFF, MACE-OFF(S), Espaloma-0.3, GAFF-2.11, and OpenFF-2.1.0). The protein and solvent were consistently modeled with AMBER ff14SB and TIP3P water. Each system was simulated for 500 ps at 300 K with a 1 fs timestep.

During the simulation, ResFF produced stable trajectories with no integration failures or abnormal temperature excursions as shown in Fig. S7 and Fig. S8. Ligand RMSD profiles are comparable to conventional MM force fields (Fig. 6e). In addition, bond-length distributions (Fig. S9) remain narrow and physically reasonable, indicating smooth force behavior near equilibrium geometries. These analyses address numerical stability rather than only structural preservation.

Overall, these results indicate that ResFF can be used in practical MD simulations without the numerical instabilities sometimes associated with machine-learned force fields. We have revised line 532-540 of Section 2.6 and the Supporting Information to clarify the scope and interpretation of these MD tests.

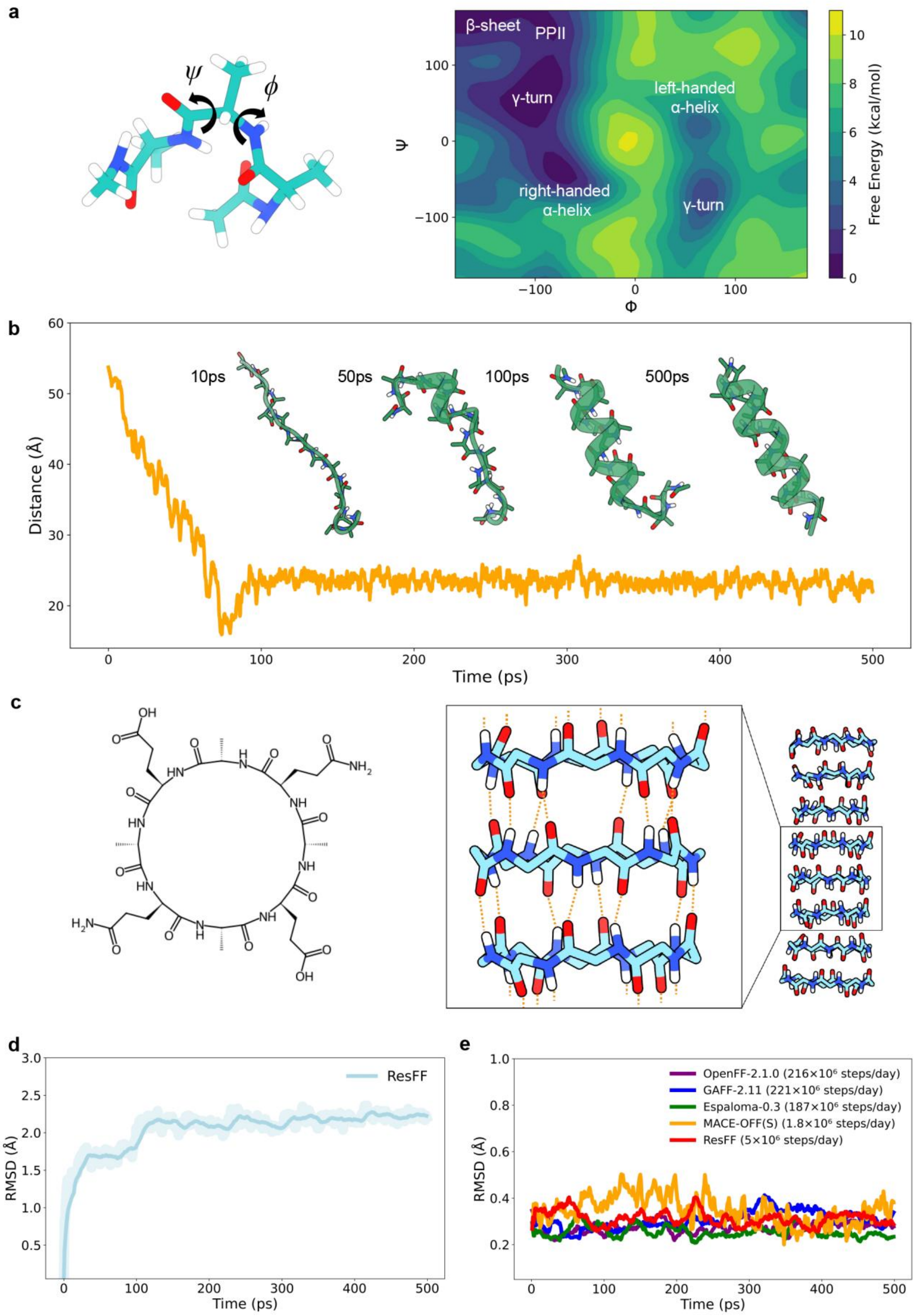

Figure 6. Molecular simulation of tetrapeptide, polyalanine, cyclic peptide (CY) assembly and Tyk2 complex systems. (a) Free energy surface of the tetrapeptide generated by ResFF in explicit solvent at 500 K for 1 ns simulation. **(b)** Polyalanine folding dynamics: time evolution of the distance between α-carbons at the N- and C-

termini during a 500 ps gas-phase simulation at 300 K with 1 fs timestep. Representative conformations at 10 ps, 50 ps, 100 ps, and 500 ps illustrate secondary structure formation. **(c)** Chemical structure of the cyclic α-alt(D,L)-peptide. Representative final snapshot of CY assembly after 500 ps of MD simulation at 300 K in solvent with 1 fs timestep, shown using backbone atoms only. The intermolecular hydrogen bonding network that stabilizes the tubular assembly is highlighted. **(d)** RMSD of the CY assembly during the simulation. **(e)** Tyk2 simulations in explicit solvent: comparison of ligand RMSD relative to the initial structure, as predicted by ResFF, MACE-OFF(S), Espaloma-0.3, GAFF-2.11 and OpenFF-2.1.0 at 300 K. The protein was parameterized with AMBER/ff14sb, and the TIP3P water was used. Simulation speeds are indicated.

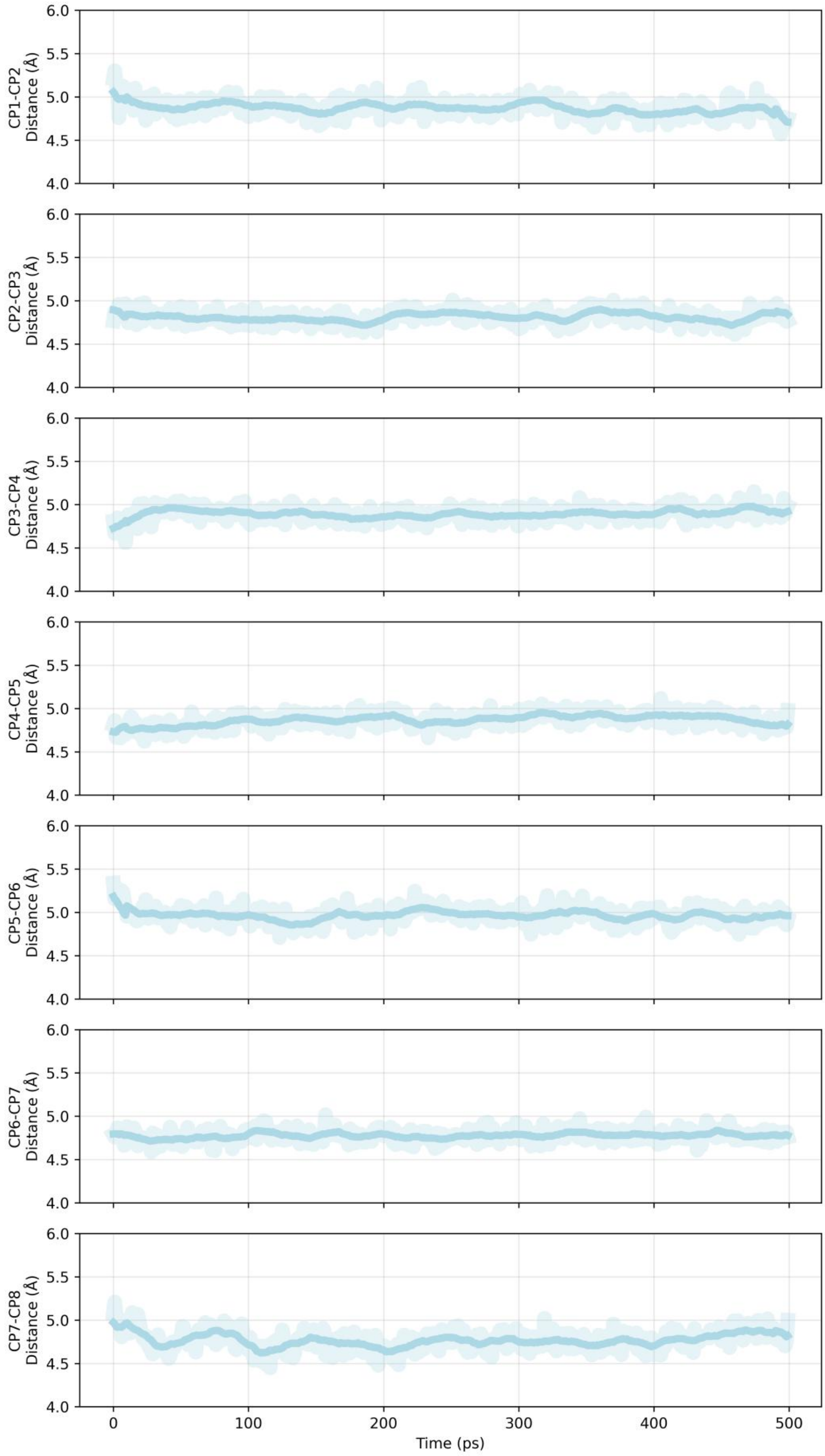

Figure S6. Inter-ring distance of each cyclic peptide (CY) in α-alt (D, L)-peptide assembly during the simulation.

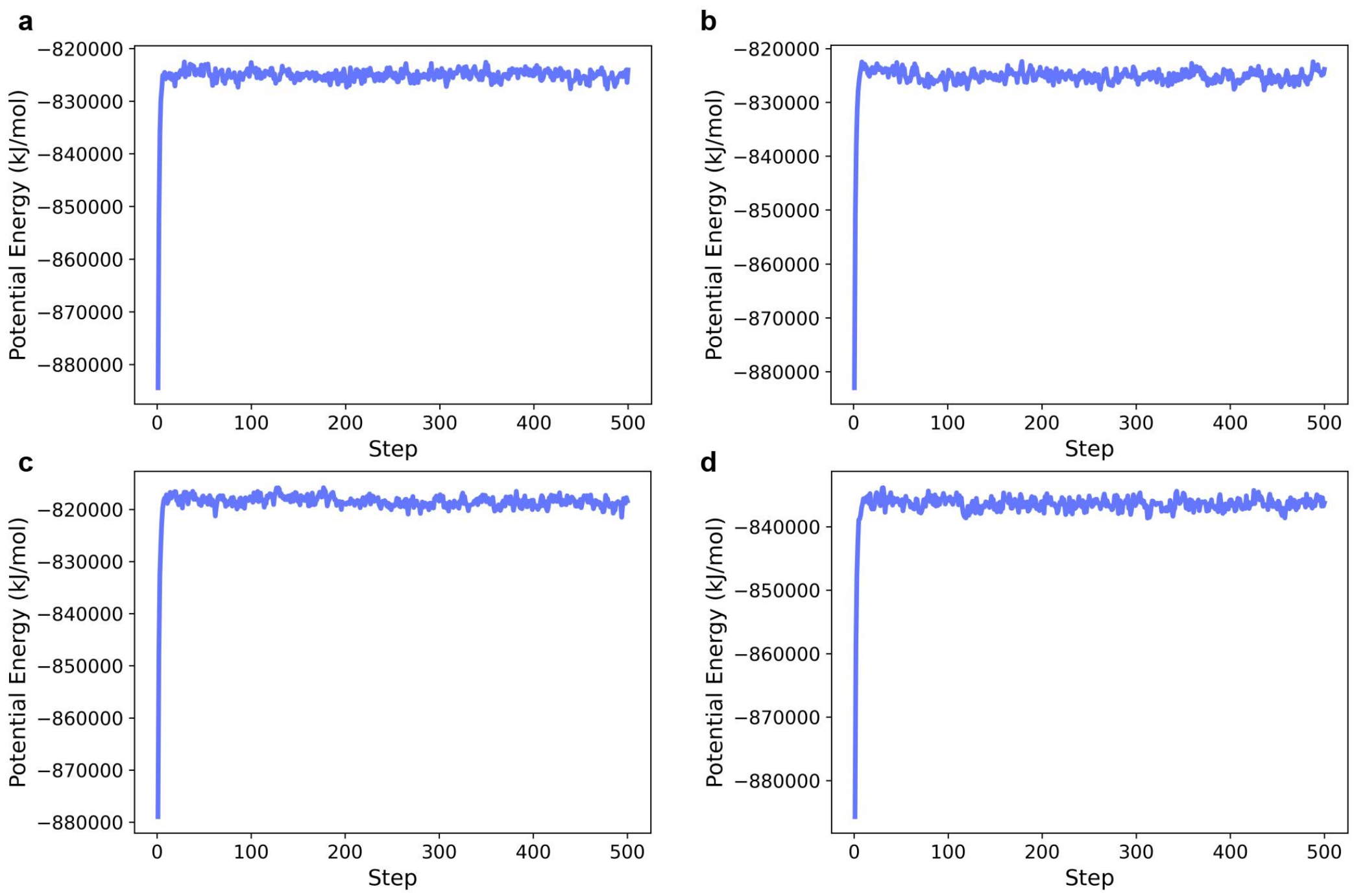

Figure S7. Potential energy fluctuation of representative force fields for the ligand in Tyk2 complex systems dynamic simulation. (a) OpenFF-2.1.0. (b) Espaloma-0.3. (c) ResFF. (d) MACE-OFF(S).

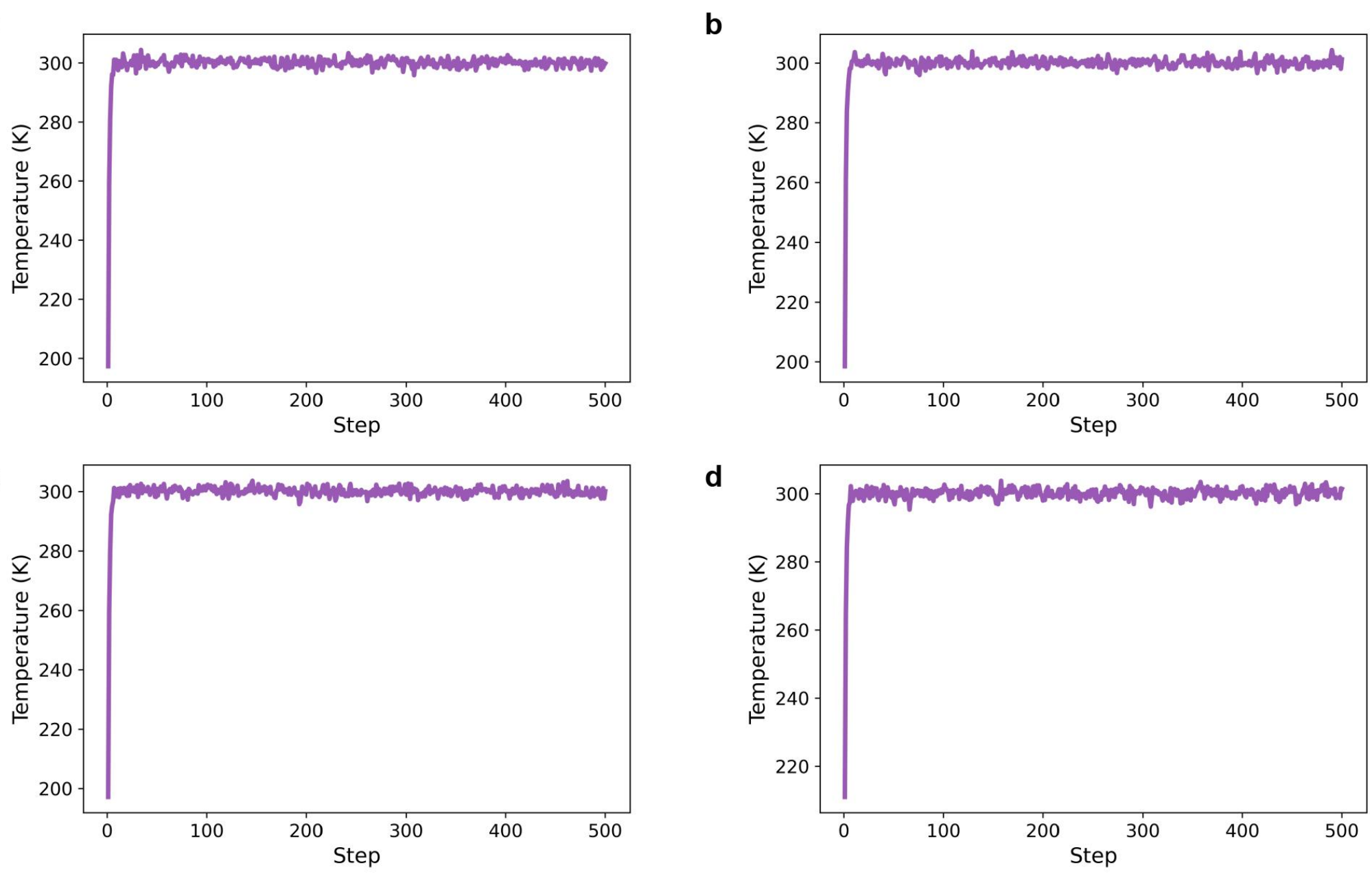

Figure S8. Temperature fluctuation of representative force fields for the ligand in Tyk2 complex systems dynamic simulation. (a) OpenFF-2.1.0. (b) Espaloma-0.3. (c) ResFF. (d) MACE-OFF(S).

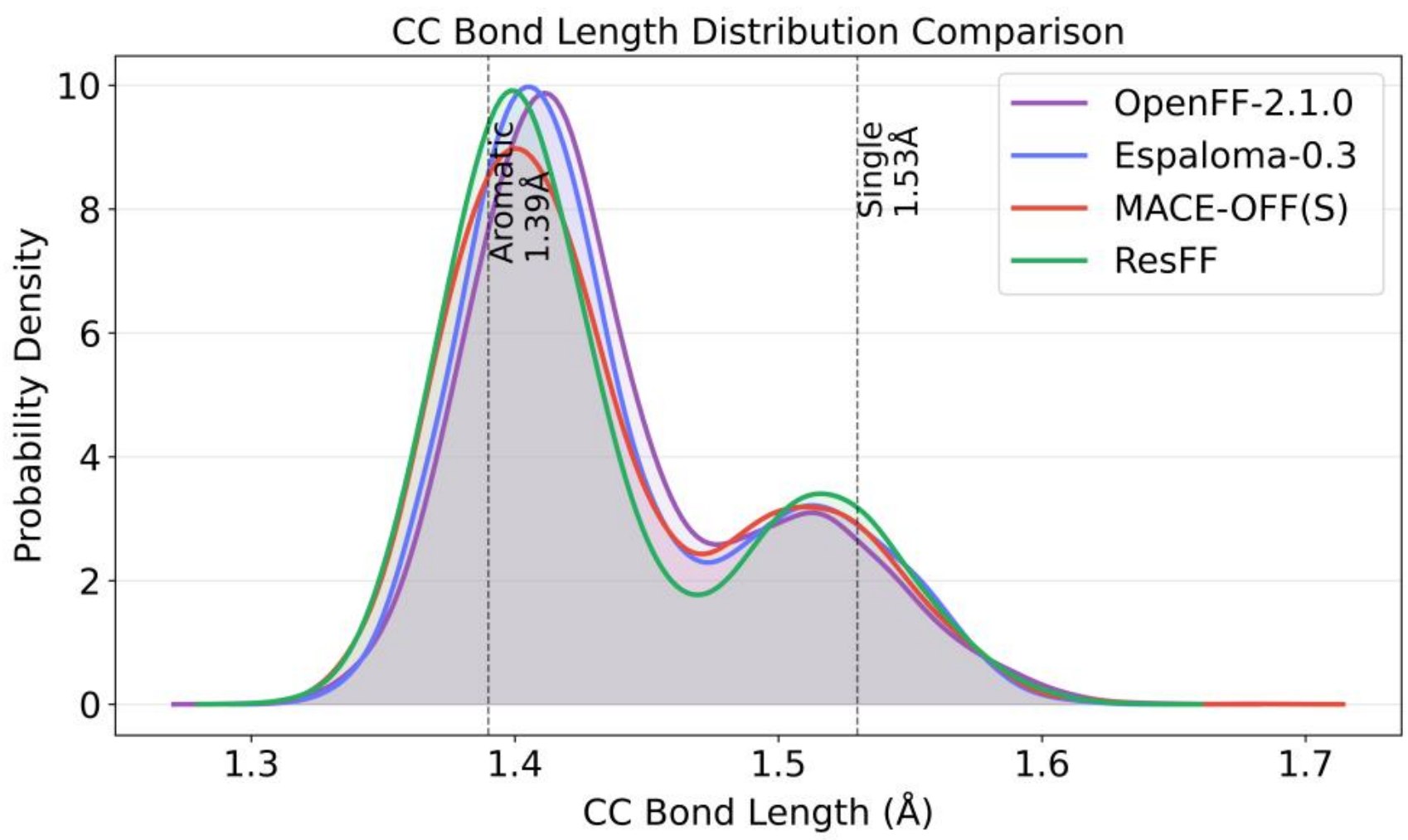

s

Figure S9. Bond length analysis of representative force fields for the ligand in Tyk2 complex systems dynamic simulation. Reference values for standard bond lengths are shown (C-C single: 1.53 Å[5]; aromatic: 1.39 Å[6]).

Reviewer #2 (Remarks to the Author):

The authors present the ResFF force field for molecular modelling in which a MM force field (bonds, angles, torsions) is supplemented by residual corrections from a neural network. The model is trained on the SPICE QM dataset to create a transferable model that is tested on various organic molecule benchmarks.

The model is clearly described and shows promising performance, but benchmarks against state-of-the-art machine learning potentials, both in the gas and condensed phase, as well as scaling tests to profile the computational expense are needed to better understand the advantages and disadvantages of the residual corrections, before publication.

Response: We would like to express our sincere gratitude for your time and professional feedback. Your comprehensive suggestions have been instrumental in refining our manuscript. We have carefully addressed all comments to enhance the rigor and quality of our work, particularly by expanding our benchmarking against state-of-the-art models and clarifying ResFF's application scope. We hope that these revisions and the detailed responses below adequately address your concerns and have made the manuscript more complete.

Major comments:

Comments 1:

The residual part of the FF seems to be used to model long-ranged electrostatics and dispersion, as well as correcting the valence terms. This is an interesting choice, as actually we know what the physics-based equations are for electrostatics and dispersion. Many machine learning potentials (MLPs) are going the other way and adding explicit electrostatics to local models (eg https://arxiv.org/abs/2507.14302). A comment on these contrasting approaches would be useful.

Response: We thank you for this insightful comment and for highlighting the conceptual contrast between our residual-learning strategy and recent ML potentials that explicitly incorporate long-range electrostatics into local neural network models.

We fully agree that electrostatics and dispersion are governed by well-established physical laws and possess known analytical forms. We would like to clarify that the residual component in ResFF is not intended replace those physically motivated treatments. As a finite-cutoff local neural network, it cannot rigorously recover these long-range functional forms at large separations, nor is it designed to replace physically motivated long-range treatments. Instead, the residual term should

be interpreted as a short-range, environment-dependent correction to the analytical MM backbone. In practice, the reference electronic structure calculations contain quantum mechanical effects that are not fully captured by standard MM functional forms. The residual module provides a data-driven correction within a finite cutoff to reduce these systematic discrepancies in the chemically relevant near-equilibrium intermolecular regime. From this perspective, the residual network enhances the local accuracy of the potential energy surface, rather than attempting to construct an explicit, physically decomposed model of long-range electrostatics.

This philosophy differs from hybrid machine learning potentials that explicitly introduce long-range electrostatics or polarization to enforce correct asymptotic behavior. Those models prioritize rigorous treatment of long-range interactions and are particularly well suited for condensed-phase thermodynamics or systems where long-range electrostatics is dominant. In contrast, ResFF adopts a modular hybrid framework in which a physically interpretable MM backbone ensures stable molecular mechanics behavior, while the residual module enhances near-equilibrium energetic accuracy. The two strategies therefore address different methodological priorities and should be viewed as complementary.

To better contextualize these contrasting approaches, we have added comparisons with representative models that explicitly encode long-range interactions, namely MACELES-OFF and ANA2B, and revised Table 3 with additional results provided in Tables S3 in the Supporting Information. MACELES-OFF extends MACE-OFF(S) through a Latent Ewald Summation framework that infers atomic charges and long-range electrostatics directly from energy and force data, while ANA2B combines a local GNN with analytical electrostatics based on atomic multipoles and dispersion corrections, trained on a substantially larger dataset with a focus on noncovalent interactions.

Since these explicit additions primarily target intermolecular electrostatic and dispersion effects, we focus our comparison on the S66×8 benchmark, a gold-standard dataset for evaluating intermolecular noncovalent interactions, which directly probes the accuracy of noncovalent interaction energy predictions. As shown in Tables 3 and Tables S3, MACELES-OFF improves upon MACE-OFF(S), achieving a RMSE of 0.41 kcal/mol, and ANA2B also shows strong performance with a RMSE of 0.60 kcal/mol. Despite not explicitly encoding analytical electrostatic or dispersion terms, ResFF attains comparable accuracy on S66×8 with a RMSE of 0.55 kcal/mol, using a

substantially smaller training dataset and a less complex model architecture. This performance arises from the residual module's ability to adaptively correct deficiencies in the underlying MM description in an environment-dependent manner.

Taken together, these results indicate that explicitly incorporating physics-based electrostatics and dispersion is a highly effective strategy for maximizing accuracy in noncovalent interactions. We have updated these results in line 358-377 of Section 2.4. At the same time, our findings suggest that a residual-learning framework offers an alternative and complementary pathway, achieving competitive accuracy and robust generalization for drug-like molecules, while preserving the modularity of the hybrid framework and seamless compatibility with classical MM force fields (enabling easy implementation in existing simulation workflows). We have revised line 172-179 of Section 2.1 to clarify this design choice, its relation to alternative hybrid ML-physics approaches, and the intended scope and limitations of ResFF.

Table 3. RMSE (kcal/mol), MAE (kcal/mol), Pearson correlation r, and coefficient of determination R^2 of different methods on the S66×8 dataset. Best results are in bold, and second-best results are underlined. Results for GAFF-2.11 and GB-FFs GAFF are collected from Chen et al.[7]. Espaloma-0.3 and MACE-OFF(S) results are generated using published model weights. ANA2B result is collected from Thürlemann et al.[8]. Noted that S66×8 was used as an early-stopping criterion during ANA2B training stage.

Methods	RMSE (kcal/mol)	MAE (kcal/mol)	Pearson's r	R^2
OpenFF-2.1.0	1.49	0.87	0.793	/
GAFF-2.11	1.84	0.94	/	/
GB-FFs GAFF	0.88	0.51	/	/
Espaloma-0.3	1.39	0.74	0.806	/
MACE-OFF(S)	0.62	<u>0.37</u>	**0.975**	<u>0.910</u>
ANA2B	<u>0.60</u>	**0.31**	/	/
ResFF	**0.55**	0.39	<u>0.970</u>	**0.929**

Table S3. Performances of different methods on the S66×8 dataset. Metrics include RMSE, MAE, Pearson correlation r, and coefficient of determination R^2. Best results are in bold, and second-best result are underlined.

Methods	RMSE (kcal/mol)	MAE (kcal/mol)	Pearson's r	R^2
MACE-OFF(S)	0.62	0.37	0.975	0.910
MACE-OFF(M)	<u>0.36</u>	<u>0.19</u>	<u>0.990</u>	<u>0.970</u>
MACE-OFF(L)	**0.31**	**0.16**	**0.993**	**0.977**
MACELES-OFF	0.41	0.21	0.985	0.953
ResFF	0.55	0.39	0.970	0.929

Comments 2:

With the above in mind, we need to see testing of ResFF for condensed phase modeling. Eg MACE-OFF is tested on densities and enthalpies of vaporization of liquids, as well as crystal data. This is a much more stringent test of the developed nonbonded interactions than what is presented here. As presented, it is unclear whether ResFF is intended for condensed phase modeling.

Response: We sincerely thank you for this important comment regarding the validation of ResFF in condensed-phase environments and for emphasizing liquid-state thermodynamic properties as stringent tests of nonbonded interactions.

We would like to clarify that ResFF is primarily designed as a generalizable molecular force field for accurately modeling intramolecular energetics and short- to intermediate-range noncovalent interactions in drug-like organic molecules, rather than as a force field explicitly optimized for condensed-phase thermodynamic properties. The model is trained on gas-phase quantum mechanical reference SPICE data, which does not directly constrain bulk observables such as liquid densities, enthalpies of vaporization, or crystal lattice parameters. To address your concern, we have now performed additional condensed-phase benchmarks as a transferability test. Specifically, we evaluated liquid densities and enthalpies of vaporization ΔH_{vap} for 39 organic molecules taken from Ref [1]. As shown in Fig. R2, ResFF achieves a high Pearson correlation coefficient of 0.95 for liquid density. This result is physically reasonable, as liquid density is predominantly governed by short- and medium-range

packing interactions and local chemical environments, which are well captured by the present model.

For the ΔH_{vap}, we observe a moderate correlation of 0.72 accompanied by a systematic deviation. This behavior is expected, since ΔH_{vap} is an integral thermodynamic quantity that is particularly sensitive to the cumulative contributions of long-range dispersion and electrostatic interactions, which fall outside the current design scope of ResFF.

For comparison, MACELES-OFF, which explicitly incorporates long-range electrostatic interactions, achieves higher correlations of 0.96 for liquid density and 0.84 for ΔH_{vap}. This improvement is consistent with its design objective and further supports the well-established view that explicit treatment of long-range corrections is essential for high-accuracy condensed-phase thermodynamic modeling.

Importantly, despite not being fitted to condensed-phase observables, ResFF remains numerically stable and exhibits strong correlation with experimental data. We therefore conclude that ResFF is suitable for molecular simulations in solution environments (e.g., drug-like molecules and protein-ligand MD) where accurate local energetics and conformational equilibria are essential, while it is not intended as a high-accuracy predictor of bulk thermodynamic properties such as heats of vaporization or crystal lattice energies. We have clarified this intended scope in the revised manuscript (Discussion, lines 581–588).

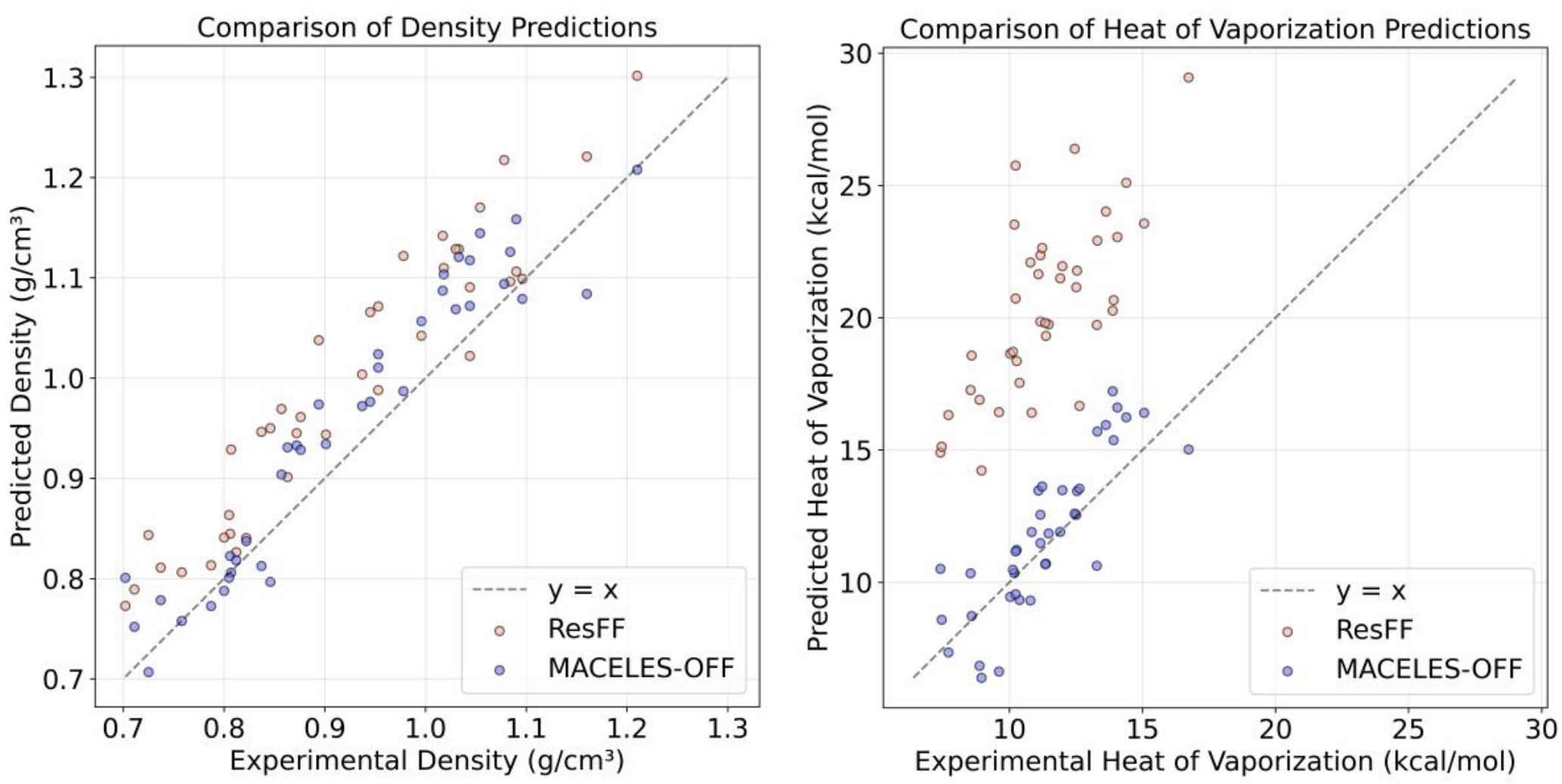

Fig. R2. Liquid property simulations of organic molecules. (left) Predicted liquid densities and (right) predicted enthalpies of vaporization ΔH_{vap} for ResFF and MACELES-OFF. Data for MACELES-OFF are sourced from Ref. [1]. ResFF

simulations were implemented in OpenMM following the condensed-phase protocol described in Ref. [2,3]. Liquid boxes containing 64 molecules were initialized using packmol[4], with box vectors adjusted to 80% of the experimental density. Prior to MD production, structures underwent energy minimization via the L-BFGS algorithm. Simulations were conducted in the NPT ensemble at 298.15 K using a Langevin integrator with 1 fs timestep and a Monte Carlo barostat with 1 atm. Trajectories were propagated for 300 ps, with the final 100 ps used for density and energy averaging. ΔH_{vap} was calculated by comparing condensed-phase energies with vacuum-phase simulations of a single non-periodic molecule, using the same averaging window.

Comments 3:

It should be noted that the MM FFs (eg OpenFF and Espaloma) are not expected to do well on the DES370K as they are effective fixed charge force fields for condensed phase modeling. Eg if we look at the water-water dimer in Fig 4, OpenFF does very badly, yet models the condensed phase properties of water very well. For the application area of these force fields, condensed phase accuracy is more important than dimer interactions.

Response: We sincerely thank you for this important clarification and fully agree with the point raised.

Classical fixed-charge molecular mechanics force fields (e.g., OpenFF and Espaloma) are primarily parameterized to reproduce condensed-phase thermodynamic observables (e.g., densities, heats of vaporization, and crystal properties), rather than to match high-level gas-phase intermolecular interaction energies. Consequently, their nonbonded parameters represent effective interactions calibrated under condensed-phase conditions, and they should not be interpreted as literal approximations to an isolated gas-phase dimer potential energy surface.Within this framework, reduced accuracy on isolated gas-phase dimers is not unexpected. Because the parameterization targets macroscopic thermodynamic behavior, many-body, polarization, and screening effects present in the condensed phase are implicitly absorbed into the effective pairwise terms. As a result, gas-phase dimer interaction energies are not necessarily reproduced with the same fidelity, and discrepancies on DES370K mainly reflect a difference in parameterization philosophy rather than a limitation within their intended application domain.

To avoid any potential misinterpretation, we would like to state explicitly that DES370K is not used in our manuscript as a ranking benchmark for condensed-phase force fields, and we do not interpret the DES370K results as evidence that ResFF is superior to condensed-phase-optimized MM force fields for bulk simulations. Instead, we use DES370K as a gas-phase diagnostic benchmark to examine how different modeling paradigms approximate quantum-mechanical intermolecular interaction energetics in near-equilibrium configurations, which is relevant for applications that depend on accurate local energetics (e.g., conformational equilibria and ligand-binding interactions).

Consistent with your emphasis that condensed-phase accuracy is the primary criterion for condensed-phase force fields, we have additionally included condensed-phase transferability tests in our response (Fig. R2), reporting liquid densities and enthalpies of vaporization (ΔH_{vap}) for 39 organic liquids. These results provide a more appropriate condensed-phase context for interpreting the nonbonded interactions.

ResFF follows a different design objective. By learning a correction to an MM baseline using QM reference data, it aims to approximate the underlying quantum-mechanical energy landscape for drug-like molecules, rather than to directly reproduce macroscopic thermodynamic observables. As a result, ResFF should not be viewed as replacing condensed-phase-optimized force fields. Classical fixed-charge force fields remain more appropriate for large-scale bulk thermodynamic simulations, whereas ResFF is intended for molecular simulations where local energetics and conformational accuracy are critical.

We have clarified this distinction and revised the relevant discussion in the lines 215–220 of Section 2.2 to avoid potential misinterpretation of the DES370K comparisons.

Comments 4:

Section 2.2. It should be noted in the main text that the experiment here is done with the small MACE model, trained only on energies (not forces), which is not the ideal method to train an MLP. Further, as I understand it, DES370K is only testing the residual part of the FF, since the bonded interactions are fixed? The higher accuracy of ResFF could just be the result of a longer-range receptive field and we can clearly see that in Fig S3 where interactions beyond the MACE cutoff are inaccurate. Why

not compare with the MACE-OFF23(L) model, with a longer-range receptive field, or even better a model such as MACELES-OFF that includes long-ranged electrostatics?

Response: We sincerely thank you for these important and technically detailed comments regarding the MACE baselines, the DES370K benchmark, and the role of receptive field and long-range interactions.

We agree that training neural network force fields using both energies and forces is generally preferable for constructing production-level machine-learning potentials. In the original manuscript, the small MACE-OFF model trained on energies only was not intended to represent the optimal achievable performance of the MACE architecture. Rather, it served as a controlled reference baseline trained under the same data availability and supervision setting as ResFF. Our objective was to compare modeling strategies and energy decomposition philosophies, not to benchmark the best possible MACE implementation.

To further address this concern, we retrained both MACE-OFF(S) and MACE-OFF(L) using combined energy and force supervision following the published protocols and hyperparameters. The resulting models (denoted MACE-OFF(S)* and MACE-OFF(L)*) show consistent improvements over the energy-only counterparts on both Gen2-Opt and DES370K in Table R3. In particular, the longer-range MACE-OFF(L)* substantially reduces errors on DES370K, confirming your expectation that intermolecular configurations in this dataset are sensitive to receptive-field length and force supervision.

We would also like to clarify the interpretation of the DES370K experiment. Although the MM covalent parameters are fixed during this stage, the predicted molecular energy is still the total energy given by the sum of the MM baseline and the residual neural correction. The dataset consists of intermolecular dimer configurations where intramolecular geometries remain near equilibrium. In this regime, the MM component provides the physically constrained molecular geometry and reference energetic baseline, while the residual network learns corrections to the full potential energy surface relative to this baseline. Therefore, DES370K evaluates noncovalent interaction energetics within a hybrid force-field framework rather than testing an isolated residual interaction model.

Regarding your important point that the improved performance could arise from a longer effective receptive field, we agree that receptive-field range is a critical factor for purely local neural potentials. NNFFs rely entirely on the information within theirs

cutoff to learn the atomic environment and reconstruct all relevant physical interactions. However, ResFF's formulation reduces the burden placed on the neural network. The MM backbone provides a physically stable reference energy landscape, while the neural component learns environment-dependent corrections primarily near equilibrium configurations. The neural network therefore does not need to reconstruct the entire interaction physics from local descriptors alone. The improved performance therefore reflects a different modeling formulation rather than simply an extended spatial receptive field. We have clarified this mechanistic distinction in the line 367-377 of Section 2.4.

Table R3. Performance of different methods on the Gen2-Opt test set and DES370K test set. Metrics include root mean squared error (RMSE), mean absolute error (MAE), pearson correlation r, and coefficient of determination R^2. Best results are in bold, and second-best results are underlined. NequIP and MACE results are derived from models trained using the settings provided in **S1** of **Supporting Information**.

Dataset	Methods	RMSE (kcal/mol)	MAE (kcal/mol)	Pearson's r	R^2
Gen2-Opt	OpenFF-2.1.0	3.76	1.83	0.630	/
	GAFF-2.11	3.77	2.14	0.609	/
	NequIP	6.81	4.66	0.473	-2.574
	MACE-OFF(S)	2.41	1.53	0.778	0.551
	MACE-OFF(S)*	2.99	1.81	0.558	0.309
	MACE-OFF(L)*	<u>2.28</u>	<u>1.34</u>	<u>0.779</u>	<u>0.600</u>
	Espaloma-0.3	3.69	1.94	0.644	/
	ResFF	**1.71**	**1.16**	**0.893**	**0.774**
DES370K	OpenFF-2.1.0	4.23	2.52	0.826	/
	GAFF-2.11	3.94	2.20	0.837	/
	NequIP	2.64	1.73	0.957	0.913
	MACE-OFF(S)	1.89	1.22	0.977	0.955
	MACE-OFF(S)*	1.82	1.14	0.979	0.959
	MACE-OFF(L)*	<u>1.37</u>	**0.86**	**0.988**	**0.976**
	Espaloma-0.3	4.68	2.78	0.768	/

ResFF	**1.36**	0.90	0.987	0.973

Finally, following your suggestion, we evaluated models that explicitly include longer-range physics. We compared against publicly available MACELES-OFF pretrained models and additional long-range-aware approaches (discussed in detail in our response to Comment 1 and updated Tables S2–S3). These models incorporate explicit electrostatics or multipole interactions and provide an appropriate reference for long-range intermolecular interactions. On the S66×8 benchmark, ResFF achieves comparable accuracy despite not explicitly parameterizing long-range electrostatics, indicating that the residual-learning framework can effectively correct near-equilibrium interaction energetics while remaining compatible with molecular mechanics simulations.

In summary, we have revised to clarify the role of the small MACE baseline (Section 2.2, line 240-245) and explain the physical meaning of the DES370K evaluation (Section 2.2, line 210), and discuss the relationship between receptive field, long-range interactions, and hybrid ML–physics modeling (Section 2.4, line 366-376).

Comments 5:

I have similar comments throughout the manuscript. The claim at the end is that ResFF out-performs the state-of-the-art. But MACE-OFF23(S) is not even the SOTA in the MACE-OFF paper. The authors should compare throughout with the large model and/or a non-local model that treats long-ranged interactions, otherwise it is not clear to me what the application area of ResFF is?

Response: We sincerely thank you for this important comment regarding the positioning of ResFF relative to state-of-the-art neural network force fields.

We fully agree that MACE-OFF23(S) does not represent the highest-accuracy model within the MACE-OFF family. To avoid misunderstanding, we have removed statements that could be interpreted as claiming that MACE-OFF(S) is the definitive SOTA baseline in the revised manuscript. Our intention is to position MACE-OFF(S) as a representative medium-scale equivariant model that enables controlled architectural comparisons.

Following your suggestion, we have added explicit comparisons with larger-capacity and long-range-aware MACE-based models, including MACE-OFF(L) and MACELES-OFF (Tables S2 and S3). Consistent with the original MACE-OFF study, these models achieve the highest absolute accuracy on torsional and intermolecular

interaction benchmarks, reflecting the benefits of increased model capacity and explicit treatment of longer-range interactions. We acknowledge that such models currently represent the upper bound of accuracy among neural network force fields evaluated here. In addition, to address your suggestion regarding non-local model, we further included a comparison with SO3LR, an equivariant neural network force field that explicitly incorporates long-range interactions. SO3LR achieves an RMSE of 1.51 kcal/mol, whereas ResFF attains a lower RMSE of 0.72 kcal/mol on TorsionNet-500. The results indicate that refined balance between neural network representation capacity and physically motivated interaction modeling appears to be critical.

Importantly, the primary goal of ResFF is not to outperform the largest or most expressive neural network force fields in absolute accuracy. Rather, ResFF is designed as a hybrid force field that targets a balanced trade-off between accuracy, generalization, and computational efficiency for drug-like molecular systems. In ResFF, a physically motivated MM module provides a stable baseline with fixed atom-type parameters assigned once at the beginning of the simulation. Classical MM force fields, however, are known to exhibit an intrinsic accuracy ceiling due to harmonic bonded approximations and simplified nonbonded forms. To mitigate these systematic deficiencies without sacrificing physical interpretability or simulation stability, we introduce a compact residual neural network (~320k parameters) that corrects the MM energy locally while preserving its inductive bias.

In this context, MACE-OFF(S) (~690k parameters) serves as the most appropriate scale-matched comparison for the residual module of ResFF, as both operate within a similar parameter regime. Larger models such as MACE-OFF(L) (~3.8M parameters) and MACELES-OFF (~2M parameters) are included to contextualize the accuracy-capacity trade-off rather than as direct architectural equivalents. While these larger NNFFs achieve superior accuracy, their design philosophy prioritizes expressive completeness of the learned potential, whereas ResFF emphasizes balanced performance within hybrid MM/ML simulations.

We have revised the line 262-265 and 276-288 of Sections 2.3 to clarify that the intended application domain of ResFF is drug-like molecular systems where a combination of physical robustness, moderate computational cost, and improved interaction accuracy over classical MM is desirable. ResFF is not positioned as a replacement for the most expressive large-scale NNFFs in scenarios where maximum attainable accuracy and explicit long-range physics are the primary objectives.

Table S2. Performances of different methods on the TorsionNet-500 dataset and Torsion Scan dataset. Metrics include RMSE, MAE, Pearson correlation r, and coefficient of determination R^2. Best results are in bold, and second-best result are underlined.

Dataset	Methods	RMSE (kcal/mol)	MAE (kcal/mol)	Pearson's r	R^2
TorsionNet -500	SO3LR	1.51	1.03	0.86	/
	MACE-OFF(S)	0.51	0.34	0.982	0.965
	MACE-OFF(M)	0.28	0.19	<u>0.995</u>	0.989
	MACE-OFF(L)	**0.21**	**0.14**	**0.997**	**0.994**
	MACELES-OFF	<u>0.22</u>	<u>0.15</u>	**0.997**	<u>0.993</u>
	ResFF	0.72	0.45	0.965	0.930
Torsion Scan	MACE-OFF(S)	0.43	0.28	0.988	0.976
	MACE-OFF(M)	0.22	0.16	<u>0.997</u>	0.994
	MACE-OFF(L)	<u>0.19</u>	<u>0.13</u>	**0.998**	<u>0.995</u>
	MACELES-OFF	**0.16**	**0.11**	**0.998**	**0.997**
	ResFF	0.90	0.50	0.953	0.896

Table S3. Performances of different methods on the S66×8 dataset. Metrics include RMSE, MAE, Pearson correlation r, and coefficient of determination R^2. Best results are in bold, and second-best result are underlined.

Methods	RMSE (kcal/mol)	MAE (kcal/mol)	Pearson's r	R^2
MACE-OFF(S)	0.62	0.37	0.975	0.910
MACE-OFF(M)	<u>0.36</u>	<u>0.19</u>	<u>0.990</u>	<u>0.970</u>
MACE-OFF(L)	**0.31**	**0.16**	**0.993**	**0.977**
MACELES-OFF	0.41	0.21	0.985	0.953
ResFF	0.55	0.39	0.970	0.929

Comments 6:

Table 2. Here the claim is that the small MACE model outperforms ResFF, but ResFF is more useful because MACE underestimates barrier heights. If MACE does better overall, but worse near the barrier, then ResFF must do worse in low energy regions? The low energy regions are the ones that are sampled in MD, so it seems more important to get these right?

Response: Thank you for this insightful comment. We fully agree that low-energy regions of torsional profiles are particularly important, as they dominate conformational populations sampled in molecular dynamics simulations.

To directly address this concern, we performed additional analyses focusing specifically on near-minimum regions of the TorsionNet-500 dataset. First, we evaluated whether the predicted global minimum torsion angle coincides with the QM reference. MACE-OFF(S) correctly identifies the QM minimum in 85.6% of cases, while ResFF does so in 83.0% of cases, indicating comparable performance in locating thermodynamically relevant conformations.

Second, we quantified energy errors in the vicinity of each QM minimum. For each torsional profile, we selected the four conformations closest to the QM minimum and computed the mean absolute error in this near-equilibrium region. MACE-OFF(S) achieves an MAE of 0.21 kcal/mol, while ResFF yields 0.29 kcal/mol. Although MACE-OFF(S) is slightly more accurate in these regions, both errors are small relative to typical torsional barriers (often 3-10 kcal/mol) and correspond to modest perturbations in Boltzmann populations at room temperature.

Importantly, systematic underestimation of torsional barrier heights, observed for MACE-OFF(S) in several profiles, can artificially increase transition rates and distort conformational kinetics, even if minima are well described. ResFF provides a more balanced representation across both minima and barrier regions, preserving near-equilibrium accuracy while reducing systematic barrier bias.

Therefore, ResFF does not sacrifice low-energy accuracy in order to improve barrier heights; rather, it maintains competitive near-minimum performance while offering a more physically consistent description of torsional energy landscapes as a whole. We have incorporated these additional analyses into the fifth paragraph of Section 2.3 to clarify the performance of ResFF in low-energy regions most relevant to MD sampling.

Comments 7:

Fig 5. The claim here is that MACE is less accurate than OpenFF for modelling energetics of the industry benchmark set? This is a very expected result that needs some diagnosis. One aspect could be that MACE is not trained on charged molecules. I suggest to investigate what happens when a model that is trained to represent these cases (such as MACE-omol (https://github.com/ACEsuit/mace/releases) or MACELES-OFF) is used. Otherwise, the authors are comparing with a model taken out of its area of applicability, and cannot make useful statements about ResFF accuracy relative to SOTA.

Response: Thank you for this important observation. We agree that the observed performance difference between the local MACE model and OpenFF on the Industry Benchmark warrants careful interpretation. In particular, model applicability, especially with respect to charged and highly polar molecules, is a critical factor when comparing force fields trained under different data regimes.

All results in Fig. 5 have now been updated in line of 405-425 of the revised Section 2.5 using the MACE-OFF(S) model as the local MACE baseline, and the comparisons were performed consistently using the ASE implementation on the OpenFF Industry Benchmark. In addition to evaluating neutral molecules with MACE-OFF(S), we further included MACELES-OFF, a MACE variant that explicitly incorporates long-range electrostatic interactions, and applied it to both neutral and charged molecular systems.

We did not include MACE-OMOL in this comparison, as it is trained on the OMOL dataset containing on the order of 10^8 molecules and employs a substantially larger model capacity. Our focus here is on drug-like molecules and force fields trained on datasets comparable to SPICE, in order to ensure a fair comparison within similar training regimes and application domains.

As shown in Fig. 5, to assess generalization behavior, we report the median values of root-mean-square deviation (RMSD) of atomic positions, torsion fingerprint deviation (TFD), and relative energy difference ($\Delta\Delta E$), together with their corresponding AUC values as a function of molecular similarity to the SPICE dataset. For molecules with high similarity to the training domain, ResFF outperforms classical MM force fields and Espaloma-0.3 on average, and achieves performance close to MACE-OFF(S). In practical applications, it is desirable that a model maintains robust performance even when extrapolating to less familiar regions of the

extensive drug-like chemical space. In this regard, for molecules with low similarity scores below 0.2, ResFF exhibits the lowest $\Delta\Delta E$ errors, indicating improved robustness when applied to structurally diverse and extrapolative scenarios.

Molecular flexibility further increases the difficulty of structure optimization due to the enlarged conformational search space. As shown in Fig. 5, the errors of classical MM force fields increase systematically with the number of rotatable bonds, whereas ResFF maintains performance comparable to MACE-OFF(S) models. For highly flexible molecules with more than 20 rotatable bonds, ResFF yields lower $\Delta\Delta E$ values relative to QM references, while preserving low RMSD and TFD, indicating accurate reproduction of both equilibrium geometries and relative conformer energetics in challenging cases.

We additionally evaluated MACELES-OFF on both neutral and charged molecules in Fig. R4. Relative to MACE-OFF(S), MACELES-OFF shows a modest improvement, consistent with the inclusion of explicit long-range electrostatic interactions and increased model capacity. These results further suggest the importance of incorporating physically motivated long-range terms when extending neural network force fields to charged and highly polar systems.

Overall, within SPICE-like chemical space ResFF remains competitive with scale-matched local NNFFs, while in more extrapolative regimes (lower SPICE similarity and higher molecular flexibility) it exhibits consistently improved robustness. As a result, ResFF can serve as a practical tool for modeling complex conformational dynamics and intermolecular interactions in real-world chemical spaces broader than the training domain.

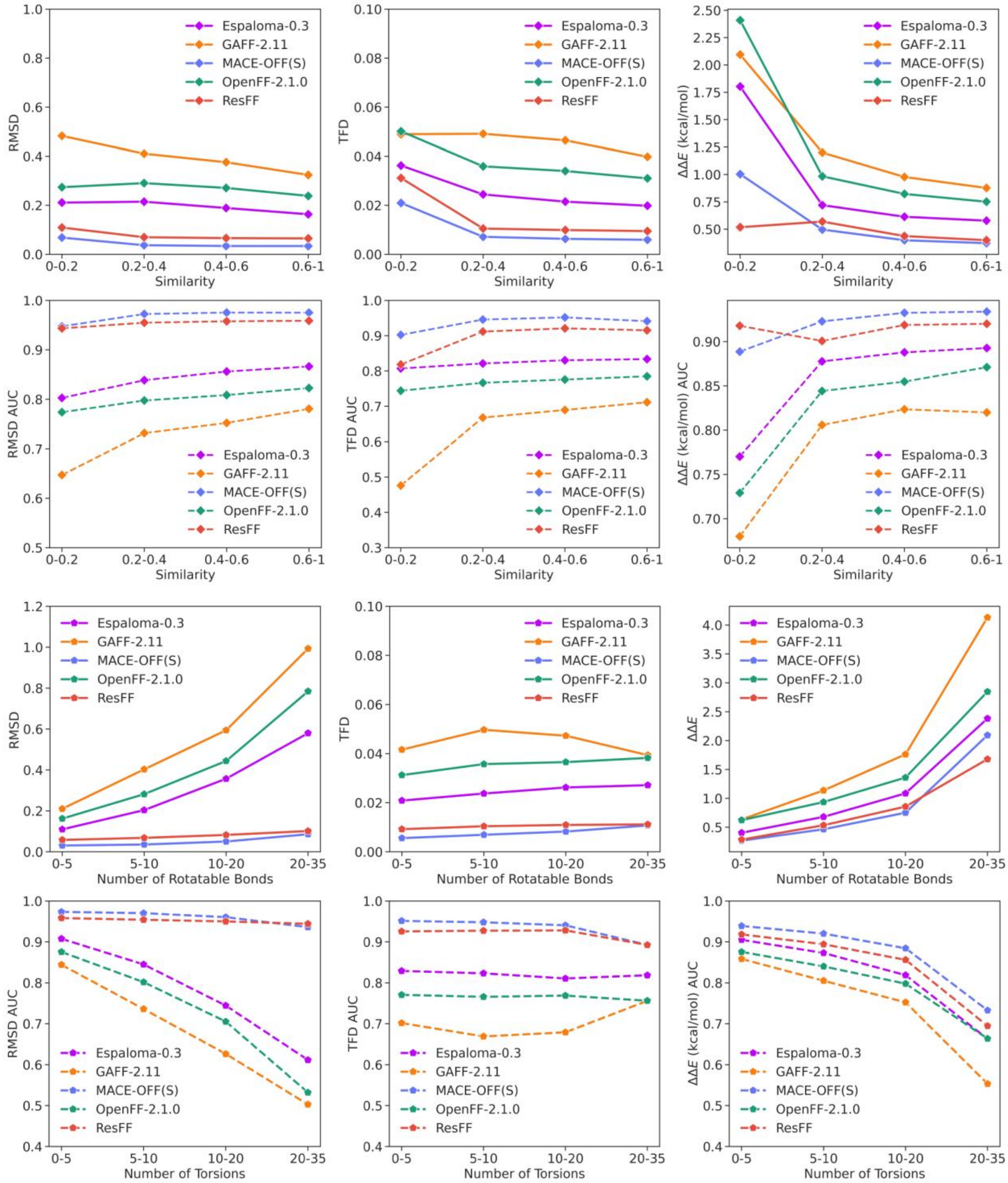

Figure 5. Metrics of different force fields on the OpenFF Industry Benchmark dataset. Median values and corresponding AUCs of root mean square deviation (RMSD, left), torsion fingerprint deviation (TFD, middle), and relative energy difference ($\Delta\Delta E$, right) for neutral molecules of different force fields, evaluated as functions of molecular similarity to the SPICE dataset and the number of rotatable bonds. ResFF and MACE-OFF(S) are conducted using a BFGS optimizer with 0.1 kcal/mol·Å as convergence implemented in ASE. Results for GAFF-2.11, OpenFF-2.1.0, and Espaloma-0.3 are adapted from Takaba et al.[9].

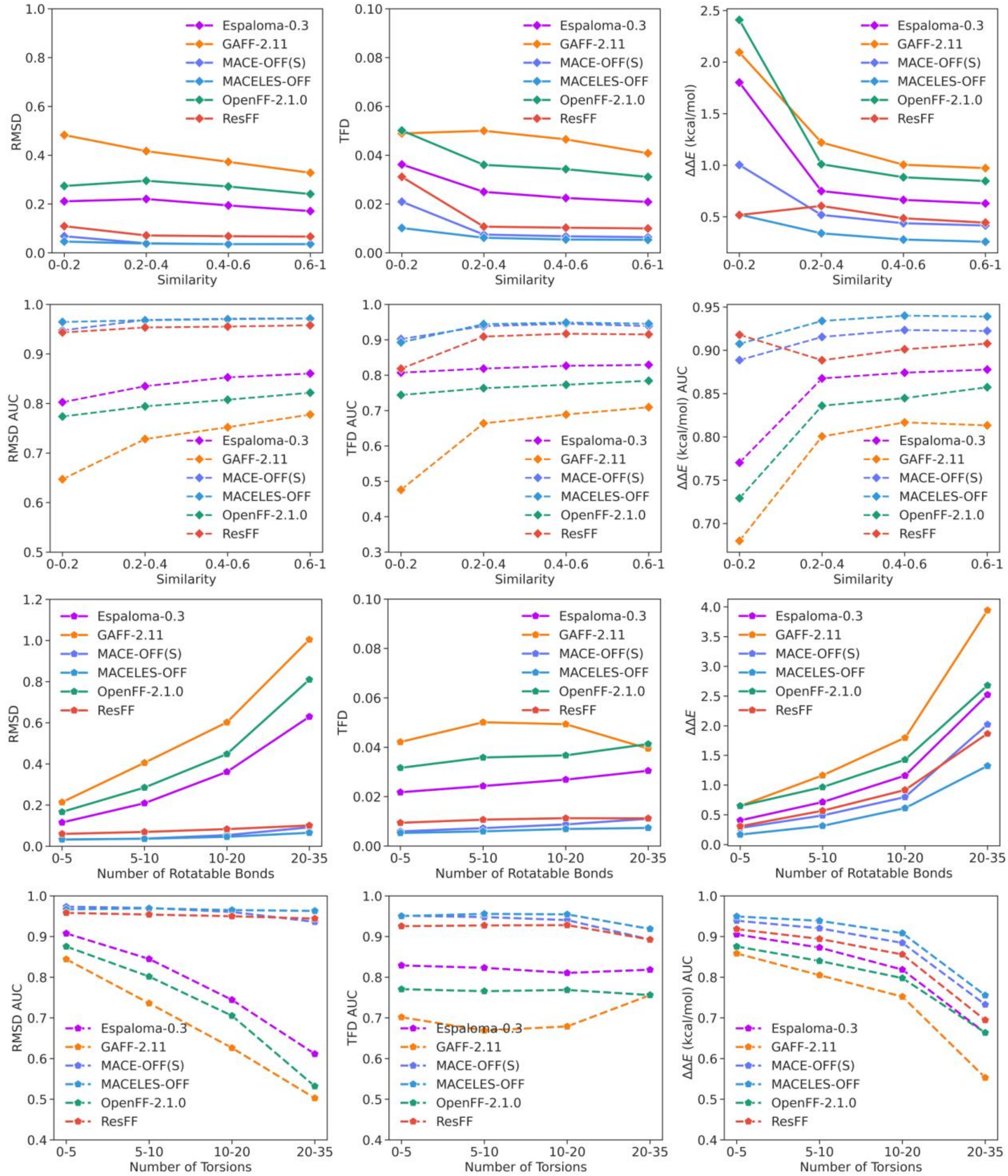

Figure R4. Metrics of different force fields on the OpenFF Industry Benchmark dataset. Median values and corresponding AUCs of root mean square deviation (RMSD, left), torsion fingerprint deviation (TFD, middle), and relative energy difference ($\Delta\Delta E$, right) for neutral and charged molecules of different force fields, evaluated as functions of molecular similarity to the SPICE dataset and the number of rotatable bonds. ResFF, MACE-OFF(S) and MACELES-OFF are conducted using a BFGS optimizer with 0.1 kcal/mol·Å as convergence implemented in ASE. Results for GAFF-2.11, OpenFF-2.1.0, and Espaloma-0.3 are adapted from Takaba et al.[9]

Comments 8:

(1) Biological testing. Why do the authors not use ResFF for the entire system? Is the MACE timing for the tetrapeptide (2.2x10^6 steps/day) measured by the authors or taken from the MACE-OFF paper? If the latter, this seems to be the time for the tetrapeptide plus water box (with everything modeled using MACE-OFF24(M), not the small model that the authors have been comparing to). (2) How does the computation time for ResFF scale with number of atoms in the system?

Response: Thank you for these important questions regarding the simulation protocol and computational performance.

(1) Use of ResFF in the biological system and timing benchmark

To avoids any potential confusion, we would like to clarify that the benchmark protocol reported in the MACE-OFF paper was constructed using AMBER14SB/ TIP3P and MACE-OFF24(M). Specifically, the original text of the MACE-OFF paper[3] states that "Figure 8 shows the free energy surface of Ala_3 in water modeled with AMBER14SB/TIP3P and MACE-OFF24(M)." In the present work, following prior studies[10,11] in the field, ResFF was intentionally applied to the region of primary chemical interest, while the surrounding environment was treated with a standard force field. This hybrid treatment is a widely adopted and methodologically sound strategy when introducing new, high-accuracy force field components, as reported in Ref. [10,11].

ResFF is designed and trained primarily on drug-like chemical space, and its MM component follows the same general philosophy as conventional molecular mechanics force fields, where parameterization is typically tailored to specific classes of systems (e.g., proteins, nucleic acids, solvents). Extending ResFF to fully heterogeneous condensed-phase systems that include periodic boundary conditions, explicit solvent, and large biomolecular environments would benefit from incorporating a broader range of chemical contexts into the training data. We view such extensions as an important and ongoing direction, rather than a limitation of the current framework.

Regarding the computational performance benchmark, the tetrapeptide timing reported in our manuscript was obtained using a consistent OpenMM simulation setup on a single NVIDIA A100 GPU. The tetrapeptide was parameterized with MACE-OFF(S) for both intra- and intermolecular interactions and solvated using TIP3P water. The same OpenMM GPU backend and hardware configuration were used for both

MACE-OFF(S) and ResFF, ensuring a fair and direct comparison. Under these conditions, ResFF achieved a performance of approximately 5×10^6 steps/day, while MACE-OFF(S) reached approximately 2.6×10^6 steps/day, averaged over three independent runs. The observed performance differences relative to previously reported values fall within the expected variability arising from differences in hardware, OpenMM versions, and simulation protocols. To ensure transparency and reproducibility, we will release the benchmark scripts on our GitHub (https://github.com/Ameki0/ResFF).

(2) Computational scaling with system size

As shown in Fig. S10, we evaluated the computational performance of ResFF across system sizes ranging from 42 atoms to 7962 atoms using OpenMM on a single A100 GPU. These results have been updated in line 568-577 of the revised Discussion. The current implementation exhibits scaling a measured slope of 0.024 ms/atom/step over the tested range. For a protein system of roughly 1,000 atoms, ResFF attains a throughput of approximately 2.5×10^6 steps/day. While the present implementation is primarily optimized for small to medium-sized systems, these results provide a clear baseline for future development. We anticipate that substantial performance improvements can be achieved through standard engineering optimizations, such as C++ acceleration and multiple-time-step integration, which are part of our ongoing work.

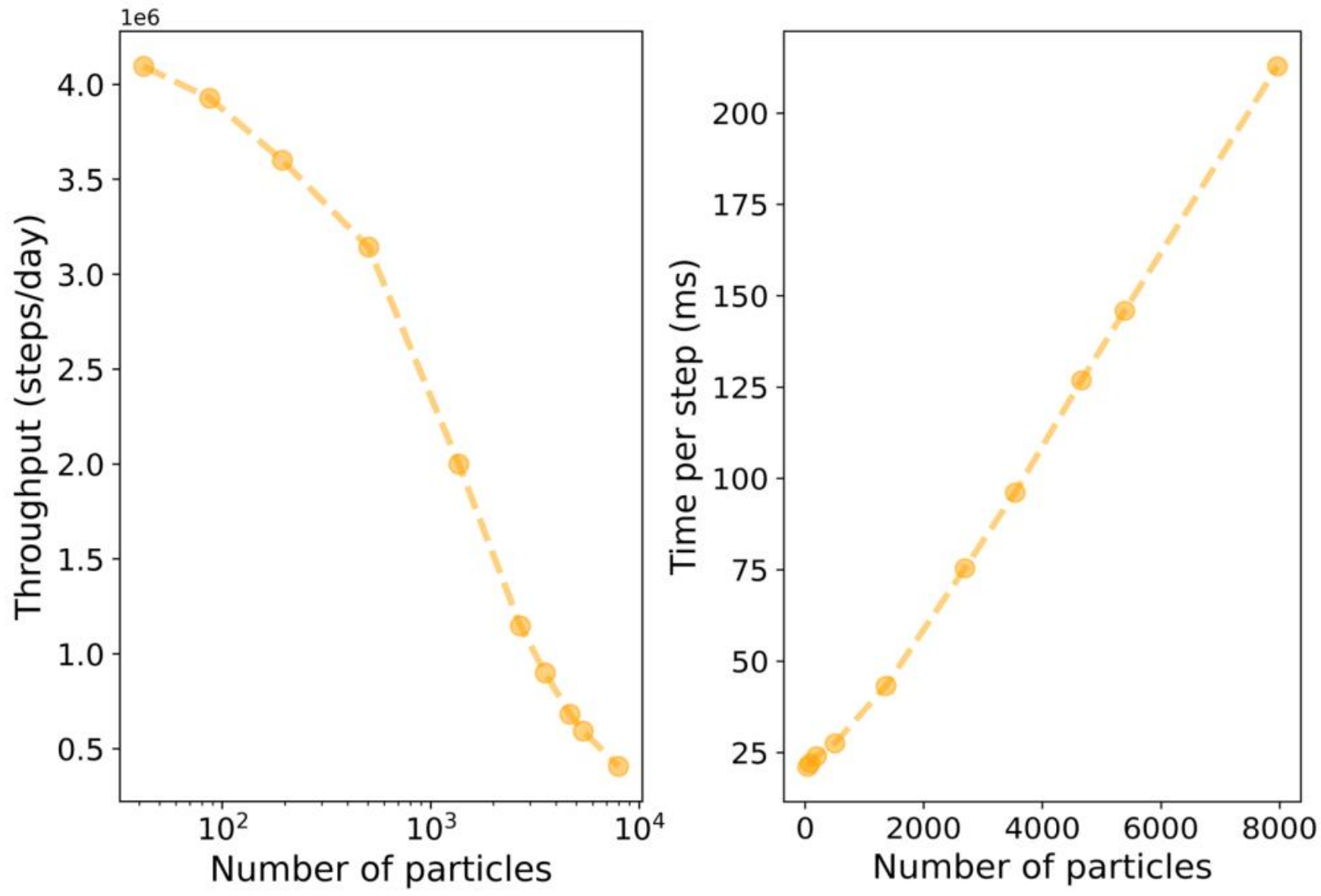

Figure S10. Scaling analysis of ResFF. The plot illustrates simulation performance for molecular systems ranging from small peptides (42 atoms) to large proteins (7962

atoms). Benchmarks were conducted on a single NVIDIA A100 GPU using OpenMM version 8.1.2.

Comments 9:

Fig 6a. The gamma-turn region observed here seems much deeper than usually overserved. How does this compare to eg the free energy surface from the AMBER force field?

Response: We sincerely appreciate you for this insightful question regarding the apparent depth of the γ-turn region in Fig. 6a.

To assess whether this feature is specific to ResFF or reflects broader sampling behavior under the chosen simulation conditions, we performed additional metadynamics simulations of the same tetrapeptide system using the AMBER force field implemented in OpenMM with explicit solvent. Identical metadynamics parameters were employed for both AMBER and ResFF to ensure a controlled comparison. Simulations were conducted at 500 K with a 1 fs timestep. The temperature was selected to match the MD22 reference conditions, which were designed to promote enhanced conformational sampling.

As shown in Fig. S4, the resulting AMBER free energy surface exhibits minima corresponding to polyproline II (PPII), anti-parallel β-sheet, and right-handed α-helical conformations, consistent with conformational preferences commonly reported in experiments and previous studies[12,13]. Although the γ-turn basin is less pronounced in the AMBER free energy surface, such conformations are not unprecedented. Structurally, the γ-turn involves three amino acid residues and corresponding inner hydrogen-bonded motif is formed with the backbone CO (i) and the backbone NH (i+2). This geometry lies near the transition region connecting left-handed and right-handed helixes that can be visited due to freedom of movement[14] in meta dynamics sampling. Such γ-turn conformations have been reported experimentally and theoretically in capped dipeptide systems and are known to be energetically accessible depending on backbone flexibility and sampling conditions[14]. Therefore, the presence of this motif in our simulations is consistent with established structural behavior of small peptide models.

These results suggest that the free energy surfaces observed for ResFF reflects enhanced conformational exploration rather than an unphysical artifact. We have updated these comparative analysis and clarification in the line 459-464 of Section 2.6 of the revised manuscript.

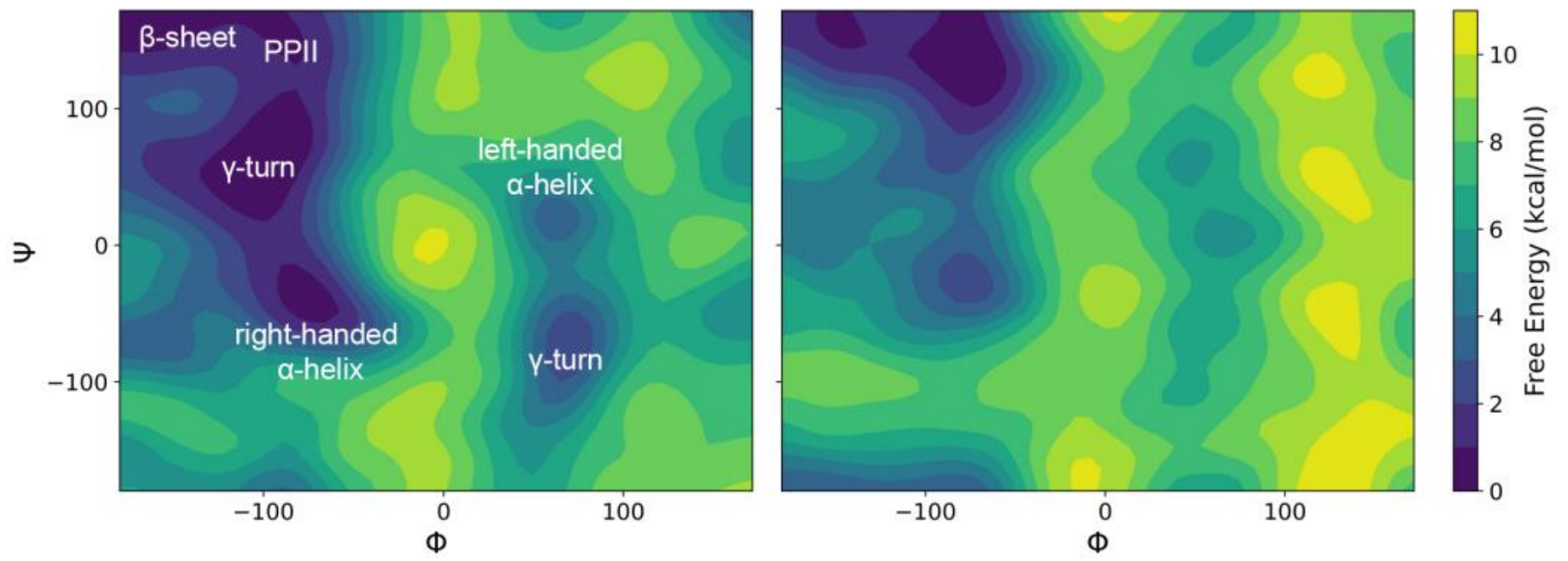

Figure S4. Free energy surface of tetrapeptide. Free energy surface of the tetrapeptide generated by ResFF (left) and AMBER (right) in explicit solvent at 500 K for 1 ns simulation.

Minor comments:

Comments 1:

Note that precision does not mean the same as accuracy. Throughout, the authors tend to use precision when I think they mean accuracy.

Response:

Thank you for this important clarification. We fully agree that precision and accuracy represent distinct statistical concepts and should not be used interchangeably, particularly in the context of force field validation and error analysis. We have carefully reviewed the entire manuscript and revised the terminology accordingly. Specifically, accuracy is now consistently used when referring to the agreement between predicted and reference values.

Comments 2:

In Table 1, why are some R^2 values missing?

Response:

Thank you for pointing this out. The missing R^2 values in Table 1 were not intentionally omitted. In our previous analysis, R^2 is not reported for methods that were not trained on the same benchmarks, as this metric primarily reflects how well a regression model reproduces a linear y=x relationship and can therefore be potentially misleading for such comparisons. In these case, absolute error metrics (MAE and RMSE) are more directly to quantify energetic accuracy in force-field benchmarking.

However, we agree that for completeness and consistency across methods, the R^2 metric should also be reported. We have therefore recalculated and added the missing R^2 values for OpenFF-2.1.0 and Espaloma-0.3 in the revised Table 1. These models,

although not trained on the Gen2-Opt dataset, were evaluated against the same ωB97X/TZVP reference level of theory, ensuring a consistent comparison.

Comments 3:

For polyalanine, please explain what is meant by wavy intermediate here? What feature of the data indicates it is present?

Response:

Thank you for requesting clarification. The term “wavy intermediate” were not introduced in the present work but follow the definition reported by Unke et al.[15]. The polyalanine folding was described as progressing from turn-rich conformations through a transient “wavy” intermediate characterized by backbone dihedral angles near ($\phi \approx 0°$, $\psi \approx 0°$), located in a sparsely populated region of the Ramachandran plot.

In the present simulations, this intermediate is identified according to the same structural criterion. A low but finite population is observed in the central Ramachandran region around ($\phi \approx 0°$, $\psi \approx 0°$) , consistent with the literature definition. This region does not correspond to a stable free-energy minimum in the calculated surface; rather, it appears as a transiently visited area connecting turn-dominated conformations to the emerging α-helical basin. Time-resolved trajectory analysis indicates short residence times in this region prior to conversion into canonical helical conformations.

To avoid ambiguity, the definition has been clarified explicitly in the revised manuscript, and a representative structure together has been added in Fig. S5a (Section 2.6, lines 476–479).

a

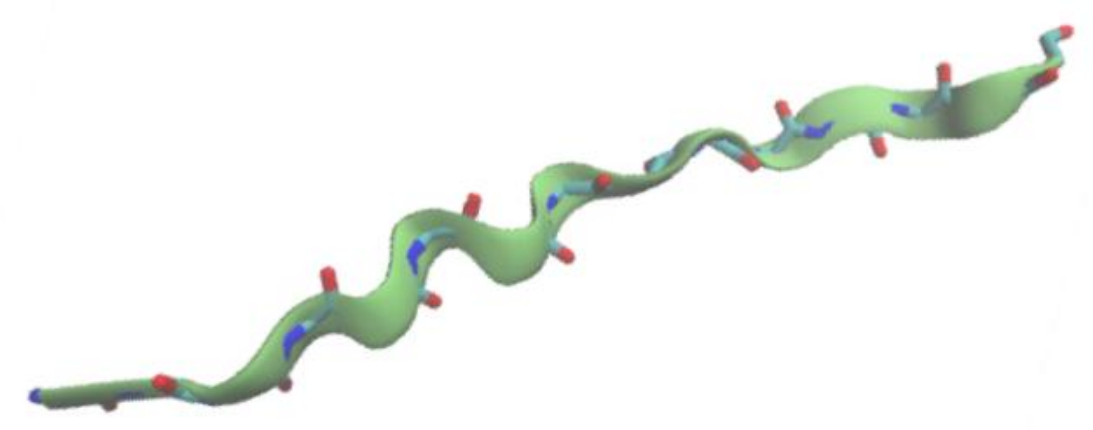

b

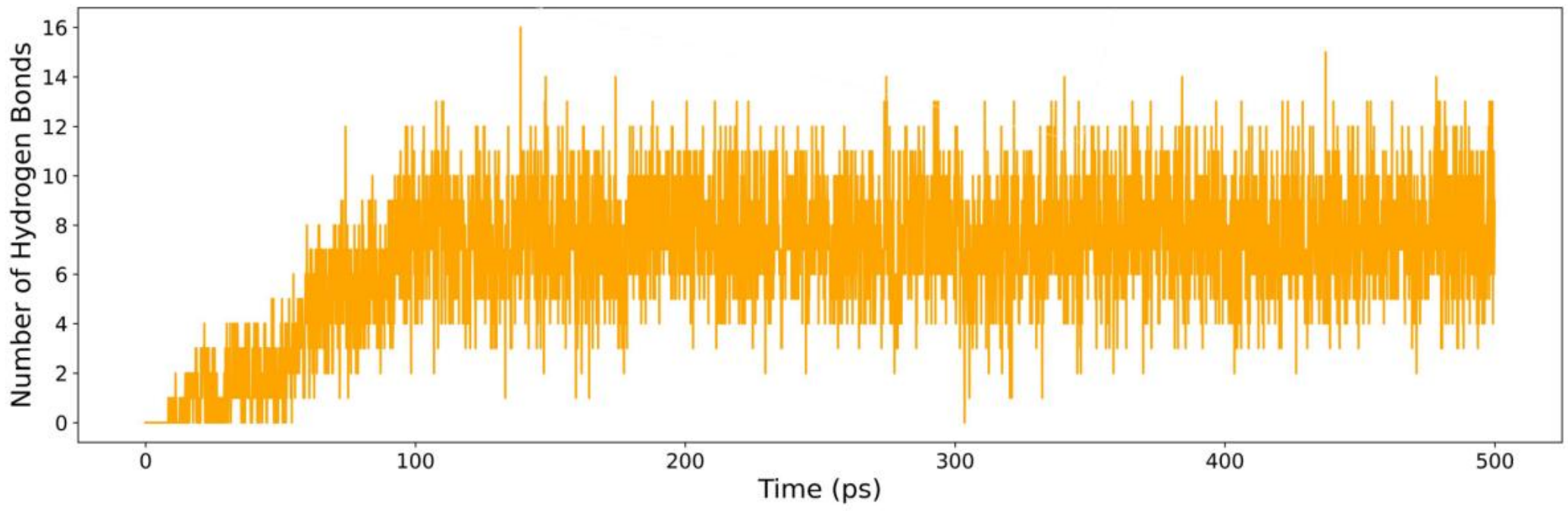

Figure S5. Polyalanines folding dynamics. (a) Illustration of short-time wavy conformation that occurs in polyalanine folding. **(b)** Number of hydrogen bonds formed during the polyalanine folding process.

Comments 4:

eq 10. The units of energy and force losses are important here when setting lambda to 1.

Response:

Thank you for this reminder. We have now added the units of the energy and force loss terms for eq 10. in line 740-741 of Method, which are expressed in kcal/mol and kcal/mol·Å, respectively.

Comments 5: eq 12. I guess there should not be a lambda on the LHS?

Response:

Thank you for pointing this out. We have corrected the eq 12. and removed the unnecessary λ.

Reviewer #2 (Remarks on code availability):

I was able to install and run the code. I could not see any information on how to reproduce the tables/figures in the paper. For example, I could not see a straightforward tutorial demonstrating how to assign force field parameters to a single molecule and e.g run molecular dynamics, nor would I know where to start to reproduce eg Fig 3c.

Response:

Thank you for carefully testing the code and for highlighting the need for clearer reproducibility guidance. Ensuring that all reported results can be independently reproduced is an essential aspect of this work.

In response, the GitHub repository (https://github.com/Ameki0/ResFF) has been substantially updated to improve transparency and usability. The following additions have been implemented:

1. Data access instructions: Direct links and detailed instructions for downloading all raw datasets used in the study, together with information on dataset preprocessing.
2. End-to-end workflow scripts: Scripts covering data preprocessing, model training, evaluation, and fine-tuning, allowing reproduction of the reported benchmarks.
3. Figure and table reproduction scripts: Dedicated example scripts and configuration files to reproduce the key figures and tables in the manuscript, including Fig. 3c, with clearly specified input data and model checkpoints.
4. Single-molecule parameterization tutorial: A step-by-step tutorial demonstrating how to assign ResFF force-field parameters to an individual molecule and perform molecular dynamics simulations using OpenMM, including environment setup and execution commands.
5. ASE interface tutorial: An additional example illustrating how to use ResFF via the ASE interface for structure optimization workflows.

The repository documentation (README and accompanying tutorials) has been reorganized to provide a clearly structured reproduction pathway. These updates are intended to ensure that readers can reproduce the published results and readily apply ResFF to their own systems.

Reference

1 Kim, D. *et al.* A universal augmentation framework for long-range electrostatics in machine learning interatomic potentials. *Journal of chemical theory and computation* (2025).

2 Batatia, I., Kov'acs, D. a. P. e., Simm, G. N. C., Ortner, C. & Csányi, G. MACE: Higher Order Equivariant Message Passing Neural Networks for Fast and Accurate Force Fields. *ArXiv* **abs/2206.07697** (2022).

3 Kovács, D. P. *et al.* MACE-OFF: Short-Range Transferable Machine Learning Force Fields for Organic Molecules. *J. Am. Chem. Soc.* **147**, 17598-17611, doi:10.1021/jacs.4c07099 (2025).

4 Martínez, L., Andrade, R., Birgin, E. G. & Martínez, J. M. PACKMOL: A package for building initial configurations for molecular dynamics simulations. *J. Comput. Chem.* **30**, 2157-2164, doi:https://doi.org/10.1002/jcc.21224 (2009).

5 Lide, D. R. *CRC Handbook of Chemistry and Physics*. (2005).

6 McClain, K. R. *et al.* Linear Inverse Sandwich Complexes of Tetraanionic Benzene Stabilized by Covalent δ-Bonding with Late Lanthanides. *J. Am. Chem. Soc.* **146**, 32708-32716, doi:10.1021/jacs.4c12278 (2024).

7 Chen, G. *et al.* Advancing Force Fields Parameterization: A Directed Graph Attention Networks Approach. *J. Chem. Theory Comput.* **20**, 5558-5569, doi:10.1021/acs.jctc.3c01421 (2024).

8 Thürlemann, M. & Riniker, S. Hybrid classical/machine-learning force fields for the accurate description of molecular condensed-phase systems. *Chemical Science* **14**, 12661-12675, doi:10.1039/D3SC04317G (2023).

9 Takaba, K. *et al.* Machine-learned molecular mechanics force fields from large-scale quantum chemical data. *Chem. Sci.* **15**, 12861-12878, doi:10.1039/D4SC00690A (2024).

10 Galvelis, R. *et al.* NNP/MM: Accelerating Molecular Dynamics Simulations with Machine Learning Potentials and Molecular Mechanics. *J. Chem. Inf. Model.* **63**, 5701-5708, doi:10.1021/acs.jcim.3c00773 (2023).

11 Sabanés Zariquiey, F. *et al.* Enhancing Protein–Ligand Binding Affinity Predictions Using Neural Network Potentials. *J. Chem. Inf. Model.* **64**, 1481-1485, doi:10.1021/acs.jcim.3c02031 (2024).

12 Hagarman, A., Measey, T. J., Mathieu, D., Schwalbe, H. & Schweitzer-Stenner, R. Intrinsic Propensities of Amino Acid Residues in GxG Peptides Inferred from Amide I′ Band Profiles and NMR Scalar Coupling Constants. *J. Am. Chem. Soc.* **132**, 540-551, doi:10.1021/ja9058052 (2010).

13 Rosenberger, D., Smith, J. S. & Garcia, A. E. Modeling of Peptides with Classical and Novel Machine Learning Force Fields: A Comparison. *J. Phys. Chem. B* **125**, 3598-3612, doi:10.1021/acs.jpcb.0c10401 (2021).

14 Sapse, A. M., Mallah-Levy, L., Daniels, S. B. & Erickson, B. W. The .gamma. turn: ab initio calculations on proline and N-acetylproline amide. *J. Am. Chem. Soc.* **109**, 3526-3529, doi:10.1021/ja00246a005 (1987).

15 Unke, O. T. *et al.* Biomolecular dynamics with machine-learned quantum-mechanical force fields trained on diverse chemical fragments. *Sci. Adv.* **10**, eadn4397, doi:10.1126/sciadv.adn4397.

Reviewer #1 (Remarks to the Author):

I appreciate that the authors have made significant improvements in the revision. The new test on the charged molecules and energy decomposition provides a clearer figure to the readers. The test on a large molecular system also demonstrates the good performance of the ResFF model. Although the limitation on the metals, reactive systems and macromolecules in my first comment has not been solved, the authors have narrowed the applicability domain in the revised manuscript. Regarding my Comment 4, the authors also claimed the limitation of current ResFF on the long-range electrostatic and dispersion in the revision, and stated its application domain in drug-like molecular systems where local interactions dominate. I think these clarifications of limitations and application domain of ResFF are good enough although some of my comments have not been addressed. Overall, I recommend this version for publication in NC.

Response: We sincerely thank you for the positive evaluation and recommendation for publication. We appreciate your constructive suggestions, which helped us more clearly define the applicability domain and limitations of ResFF.

Reviewer #1 (Remarks on code availability):

The code information is clear with detailed comments, which is usable resource for the community.

Response: Thank you for the positive feedback and appreciate the constructive suggestions that helped improve the usability and reproducibility of the code.

Reviewer #2 (Remarks to the Author):

The authors have, for the most part, responded sufficiently to the comments raised. As such, the strengths and limitations of the ResFF approach are much clearer.

Response: Thank you for the careful re-evaluation of our manuscript and for the positive assessment of the revisions. We appreciate your constructive feedback, which has helped clarify the strengths, limitations, and applicability domain of ResFF. In response to the remaining comments, we have further rechecked the relevant analyses and revised the manuscript accordingly.

Comments 1:

I am still uncomfortable with the R^2 data in Table 1, particularly the negative values, and I suggest the authors re-check these. I understand this can happen, but not why these models should look so bad. Do the data need to be shifted so that the means align?

Response: Thank you for your insightful comments regarding the R^2 metrics. We agree that negative R^2 values in Table 1 can be counter-intuitive, and we confirm that the reported values are correct and arise from evaluating R^2 on per-molecule mean-centered relative energies rather than raw absolute energies.

Specifically, for each molecule, both the predicted and reference energies were shifted by subtracting their corresponding molecular mean before calculating R^2. This procedure removes arbitrary molecule-dependent energy offsets and evaluates how well each method reproduces conformational energy differences, which are more relevant than absolute total energies for force-field and molecular simulation applications.

Under this definition, negative R^2 values can still occur when the residual error in relative energies is larger than the variance of the reference relative energies. This is particularly possible for models whose absolute total energies are strongly correlated with QM values but whose local conformational energy differences are less accurately reproduced. In contrast, calculating R^2 directly from raw absolute energies can yield misleadingly high values because the total energy scale across different molecules dominates the variance and masks conformer-level errors.

Thus, the negative values in Table 1 do not arise from an uncorrected global mean offset. Rather, they reflect the stricter evaluation of relative conformational energetics after mean-centering. To avoid ambiguity, we have added a clarification in

Section 2.2 and revised the Table 1 caption to state that all metrics including R^2 values are computed from per-molecule mean-centered relative energies.

To ensure reproducibility and transparency, the training parameters, model checkpoints, and source data for all NNFFs evaluated in this study have been made publicly available on our GitHub repository (https://github.com/Ameki0/ResFF).

Comments 2:

The authors have mis-interpreted the statement on speed in the MACE-OFF paper. "Figure 8 shows the free energy surface of Ala3 in water, modeled by AMBER14SB/TIP3P and MACE-OFF24(M)". They mean that the top panel in Fig 8 is modeled by AMBER14SB/TIP3P (ie classical) and the bottom panel with MACE-OFF24(M) (ie MLP). You can also see in Figure 12, a throughput of around 3M steps/day for ~1K atoms. I do not think it is worth discussing at length. The authors can just remove this statement, and mention that the two methods are similar in throughput.

Response: Thank you for this clarification. We have removed the potentially misleading statement and now state only that ResFF and MACE-OFF(S) exhibit comparable throughput under our benchmark settings in Discussion (lines 572-574).

Comments 3:

p32. The authors have provided condensed phase benchmarks in their response, but I do not see them in the revised paper. I suggest to include, at least in SI, as it will stop others from trying to apply the methods outside their intended application area.

Response: Thank you for this helpful suggestion. We have added the condensed-phase benchmarks to the Supporting Information (Fig. S11) and revised Discussion (lines 588–593) to clarify that these results define the current applicability boundary of ResFF, rather than implying general condensed-phase optimization.

Comments 4:

p34. Please also mention how improper torsions are handled.

Response: Thank you for pointing this out. We have clarified the treatment of improper torsions in the revised Methods section (p34, lines 642–654).

In ResFF, improper torsions are included as a distinct class of torsional interactions in the MM module, primarily to describe out-of-plane distortions and

maintain local planarity or stereochemical stability. Proper and improper torsions are identified separately from molecular topology and assigned independent learned force constants, but both are evaluated using the same periodic Fourier functional form commonly used in molecular mechanics force fields:

$$E_{\mathrm{MM}}(R) = E_{\mathrm{bond}} + E_{\mathrm{angle}} + E_{\mathrm{proper\ torsion}} + E_{\mathrm{improper\ torsion}}$$

$$E_{\mathrm{proper\ torsion}} = \sum_{\mathrm{proper\ torsion}} \sum_{n} K_{\varphi,\ \mathrm{proper},\ n}[1 + \cos(n\varphi_{\mathrm{proper},i,j,k,l} - \varphi_0)]$$

$$E_{\mathrm{improper\ torsion}} = \sum_{\mathrm{improper\ torsion}} \sum_{n} K_{\varphi,\ \mathrm{improper},\ n}[1 + \cos(n\varphi_{\mathrm{improper},\ i,j,k,l} - \varphi_0)]$$

The force constants $K_{\varphi,\ \mathrm{proper},\ n}$ and $K_{\varphi,\ \mathrm{improper},\ n}$ for proper and improper torsion terms are parameterized separately, with periodicities n=1 to 6 and the phase angle fixed at φ_0 = 0. We have also updated Fig. 1 and its legend to explicitly indicate that the MM module predicts parameters for both proper and improper torsions.

Comments 5:

p39. The figure of 73301 drug-like molecules does not appear to match the data in Table 4?

Response: Thank you for pointing out this inconsistency. We have rechecked the OpenFF Industry Benchmark statistics and corrected Table 4 accordingly. The number of drug-like molecular conformations is now consistently reported as 73,301 throughout the manuscript.

Reviewer #2 (Remarks on code availability):

I have not re-run the code after revision, but I thank the authors for updating the repository with the requested changes.

Response: Thank you for acknowledging the repository updates. We appreciate your earlier suggestions, which helped us improve the clarity, usability, and reproducibility of the codebase.
